# Chang’e-6 farside anorthosites indicate hemispherically comparable magma ocean solidification

**DOI:** 10.1038/s41467-026-73258-y

**Published:** 2026-05-15

**Authors:** Zeling Wang, Haojie Chen, Yi Chen, Bin Su, Ross N. Mitchell, Qin Zhou, Saihong Yang, Zongyu Yue, Lihui Jia, Di Zhang, Xiaoguang Li, Jiangyan Yuan, Shitou Wu, Lijun Liu, Qiu-Li Li, Chun-Lai Li, Xian-Hua Li, Fu-Yuan Wu

**Affiliations:** 1 State Key Laboratory of Lithospheric and Environmental Coevolution, Institute of Geology and Geophysics, Chinese Academy of Sciences, Beijing, China; 2 College of Earth and Planetary Sciences, University of Chinese Academy of Sciences, Beijing, China; 3 Key Laboratory of Lunar and Deep Space Exploration, National Astronomical Observatories, Chinese Academy of Sciences, Beijing, China; 4 Key Laboratory of Planetary Science and Frontier Technology, Institute of Geology and Geophysics, Chinese Academy of Sciences, Beijing, China

**Keywords:** Petrology, Early solar system, Geochemistry

## Abstract

Ferroan anorthosites (FANs) reflect the nature of the Moon’s crust formed during the late-stage lunar magma ocean (LMO). Remote sensing suggests a hemispheric dichotomy in crustal composition, with the farside anorthositic highlands being more magnesian than the nearside. Lacking direct compositional and chronological constraints from farside anorthosites, whether this crustal dichotomy reflects asynchronous LMO solidification or post-LMO crustal reworking remains uncertain. Here we present an integrated petrological, geochemical, and geochronological study of farside anorthosite clasts returned by the Chang’e-6 mission. These clasts exhibit both mineralogical and compositional similarity with nearside Apollo FANs, supporting a comparable LMO-derived primary crust on both hemispheres. A zircon-bearing anorthosite domain contains recrystallised plagioclase enriched in rare earth elements (REE), thorium, and phosphorus, suggesting thermal reworking and metasomatism by a KREEP (potassium, REE, and phosphorus)-rich magma. High-precision lead-lead dating of zircon constrains this reworking event to 4,410 ± 8 Ma, establishing a local lower bound for farside LMO solidification. These findings establish a critical chronological benchmark for farside LMO solidification and offer direct evidence of a comparable primary crust across the Moon, constraining the origin of the crustal dichotomy to post-LMO reworking.

## Abstract (web summary)

Returned farside lunar anorthosites have compositions similar to nearside counterparts and show a 4.41-Ga zircon age, indicating globally comparable magma ocean solidification and constraining the crustal dichotomy to post-magma-ocean reworking.

## Introduction

The Moon is thought to have differentiated from a global magma ocean, the lunar magma ocean (LMO)^1,2^. LMO differentiation produced a dense cumulate mantle and a buoyant anorthositic crust^1,3^, presumably initially creating a layered, spherically symmetric Moon^4^. However, orbital observations have revealed a striking hemispheric dichotomy in the composition and thickness of the ancient lunar crust, with the farside anorthositic highlands being more magnesian and thicker than their nearside counterpart^5–8^. This crustal dichotomy has been mainly attributed to asymmetric LMO crystallisation^9,10^, post-LMO large impacts^11^, or magma underplating^12^. Despite such hypotheses, whether the observed magnesian enrichment of mafic components on the farside highlands reflects the primary LMO crust remains unknown^12^. Evaluating the crustal asymmetry model involves comparing the compositions and ages of crustal products derived from the LMO from both lunar hemispheres. Ferroan anorthosites (FANs), which dominate the lunar crust and mark the late stage of LMO solidification^13,14^, represent an ideal candidate for such investigations. Studies of Apollo samples have shown that nearside FANs consist of >90% plagioclase (An = molar Ca/(Ca+Na+K) × 100 = 94–98) and minor mafic minerals (Mg# = molar Mg/(Mg+Fe) × 100 = 40–74) (refs. ^14,15^), and yield scattered ages ranging from ca. 4.5–4.3 billion years ago^16–18^. However, the composition and age of farside anorthosites and their role in LMO evolution remain largely unknown, leaving a critical gap in a globally representative understanding of the Moon’s early differentiation history.

China’s Chang’e-6 (CE-6) mission returned farside samples from a south mare unit in the Apollo basin within the northeast of the South Pole-Aitken (SPA) basin^19^. In this study, we analysed ~2000 fragments (>200 μm) from two soil samples (CE6C0100JYFM002, 5 g; CE6C0200YJFM001, 5 g) provided by the China National Space Administration (CNSA). The fragments consist of mare basalt (35%), breccia (45%), glass (15%), and non-mare lithic clasts (5%), with an enrichment in basaltic fragments and glasses relative to those in the regolith from nearside mare sites^20^. The non-mare lithic clasts are mainly composed of norite (>80%), with minor gabbronorite (~10%) and anorthosite (<10%). We focus on the anorthosite clasts here, as they are considered the dominant crustal material^13,14^, offering a unique opportunity to investigate the nature of the farside LMO and test competing models for the origin of the ancient lunar crustal dichotomy.

## Results

### Chang’e-6 anorthosites

**Fig. 1:**
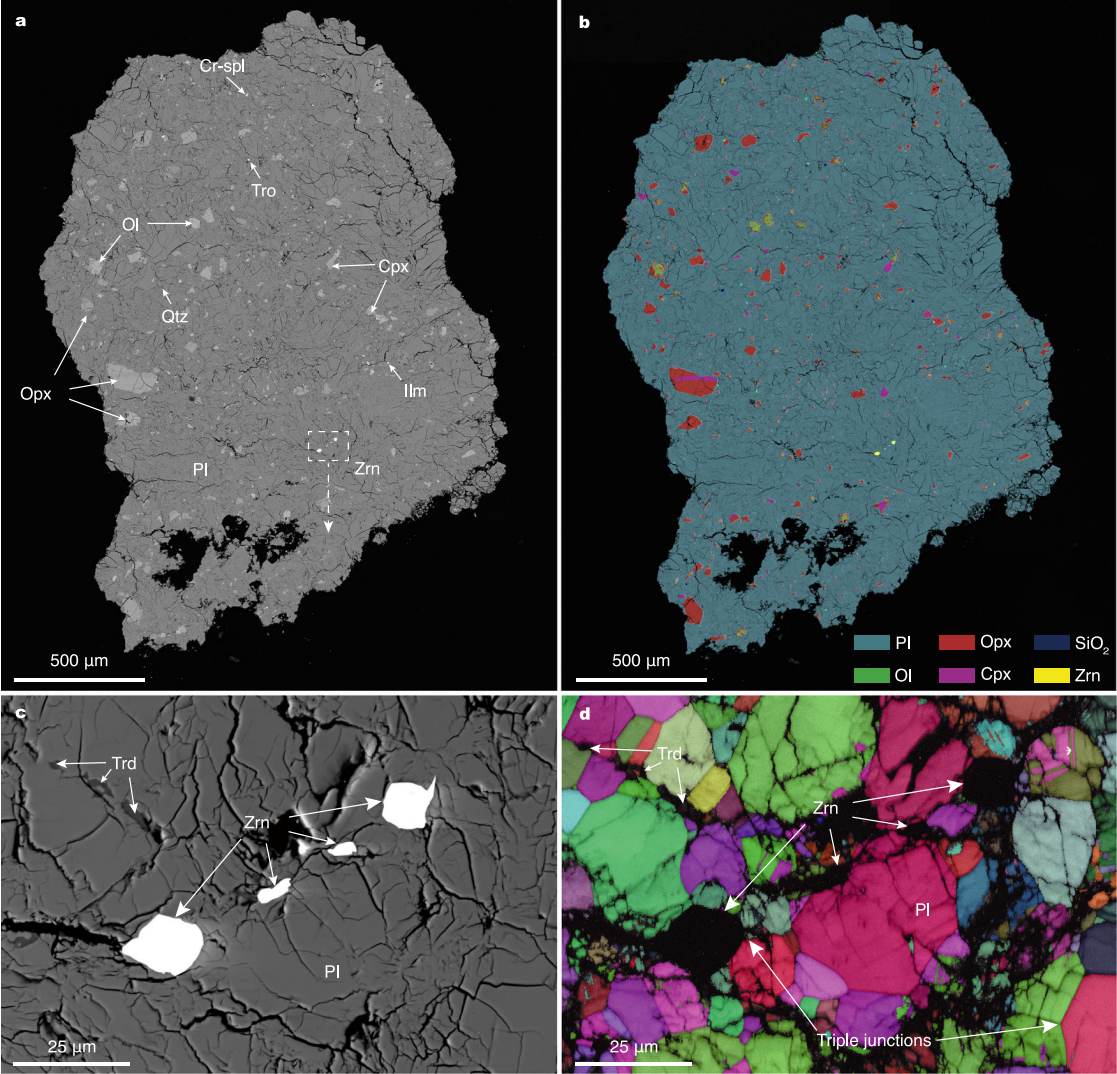
Mineralogy and microtexture of CE-6 anorthosite clast 565GP01. **a** Backscattered electron (BSE) image showing the largest anorthosite clast in the studied samples. **b** Energy-dispersive X-ray spectroscopy (EDS) map highlighting mineral distribution. **c** BSE image of the boxed region in (**a**) (white rectangle), revealing zircon and tridymite grains intergrown with plagioclase. **d** Electron backscatter diffraction (EBSD) image of the area in (**c**), exhibiting an equilibrium granoblastic texture characterised by ~120° triple junctions between fine-grained plagioclase and zircon. Cr-spl chromium-spinel, Cpx clinopyroxene, Ilm ilmenite, Ol olivine, Opx orthopyroxene, Pl plagioclase, Qtz quartz, Trd tridymite, Tro troilite, Zrn zircon.

**Fig. 2:**
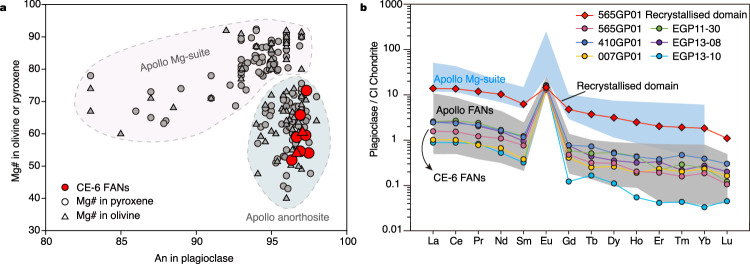
Mineral major element compositions and plagioclase REE systematics of CE-6 anorthosite clasts. **a** Plot of An in plagioclase versus Mg# of mafic minerals in CE-6 FANs. **b** Chondrite-normalised REE patterns of CE-6 plagioclase. The normalised values are from ref. ^87^. Plagioclase compositions of Apollo FANs and Mg-suite rocks were listed in Source data.

This study focuses on seven representative anorthosite clasts for petrological and geochemical analyses. The studied clasts are 0.3–2 mm in size and dominated by plagioclase (90.6–97.5 vol%) and low-calcium (Ca) pyroxene (2.1–8.6 vol%), with minor high-Ca pyroxene, olivine, chromium (Cr)-spinel, ilmenite, troilite, silica phases (quartz and tridymite), and zircon (Fig. 1, Supplementary Fig. 1, and Supplementary Table 1). These clasts were identified as anorthosite according to their mineral abundances and petrological definition^21^ (Supplementary Fig. 2). The mineral abundances and compositions of these clasts align with FAN affinity, as evidenced by An values of plagioclase (95–98) and Mg# values of pyroxene and olivine (33–70), which overlap with the compositional range of Apollo highland FANs (Fig. 2a, Supplementary Figs. 2, 3, and Supplementary Tables 2 and 3). Notably, subhedral low-Ca pyroxene grains exhibit 3–5 μm exsolution lamellae of high-Ca pyroxene (Fig. 1a and Supplementary Fig. 1), a typical plutonic, slow-cooling texture in Apollo highland rocks^22,23^. Minor ilmenite and troilite occur as inclusions in plagioclase or as interstitial phases. Silica phases associated with plagioclase have been identified in clast 565GP01 (Fig. 1 and Supplementary Fig. 4), possibly resulting from late-stage LMO solidification^24^ or subsequent recrystallisation^25^. A distinct 0.3 × 0.4 mm² recrystallisation domain within this clast shows a granoblastic texture, as evident in Cathodoluminescence (CL) and electron backscatter diffraction (EBSD) images (Fig. 1d and Supplementary Figs. 5 and 6). This domain consists of fine-grained plagioclase (10–30 μm) with straight grain boundaries and equilibrated ~120° triple junctions. Four zircon grains within this domain occur as xenomorphic, interstitial crystals in contact with plagioclase (Fig. 1c, d and Supplementary Figs. 5 and 6), indicating that they either formed during recrystallisation or represent impact-derived detrital zircon.

The consistency between the CE-6 anorthosite and Apollo FANs is also evidenced by trace element systematics: olivine Cr concentrations (35–300 μg g^−1^; Supplementary Fig. 3g and Supplementary Table 4), plagioclase cerium (Ce; 0.26–3.23 μg g^−1^) and samarium (Sm; 0.03–0.30 μg g^−1^) concentrations and rare earth element (REE) patterns (Fig. 2b, Supplementary Fig. 7, and Supplementary Tables 5 and 6). However, plagioclase within the recrystallisation domain exhibits marked geochemical deviations, including elevated phosphorus (P; 168 μg g^−1^ on average) and thorium (Th; 0.5 μg g^−1^ on average) concentrations (Supplementary Fig. 7a, b and Supplementary Tables 6 and 7), a reduced europium anomaly (Eu/Eu* = 2.65), and higher REE concentrations relative to pristine plagioclase (Fig. 2b and Supplementary Fig. 7c, d). These anomalies correlate with shifts in Sm/Ce (0.12) and Eu/Sm (0.89) ratios (Supplementary Fig. 7c, d), converging toward values diagnostic of Mg-suite plagioclase.

### Zircon Pb–Pb geochronology

**Fig. 3:**
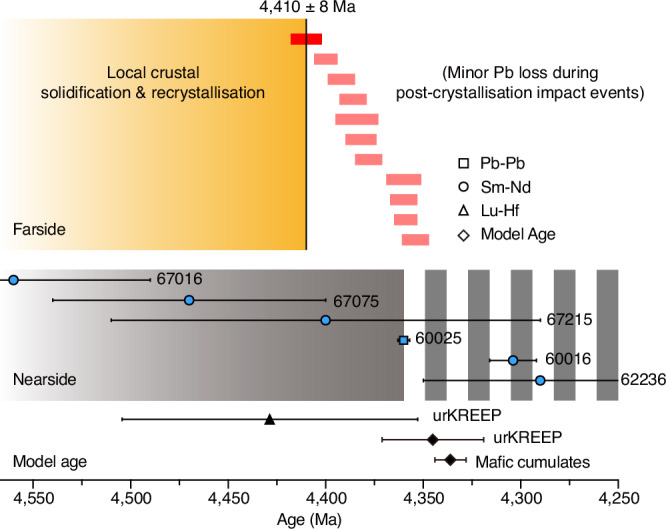
Chronological framework for lunar FANs. Red boxes represent zircon Pb–Pb dates from the anorthosite clast 565GP01, with box widths representing 1σ uncertainties. The oldest date (4410 ± 8 Ma) marks the timing of granulite-facies recrystallisation. The orange-shaded area denotes the age range of farside FANs solidification and recrystallisation. The ages of nearside Apollo FANs and the model ages of mafic mantle cumulate and urKREEP (with 1σ errors) derived from Pb–Pb, Sm–Nd, and Lu–Hf data are shown for comparison. Raw age data were listed in Source data.

The four zircon grains observed within the recrystallised domain of clast 565GP01 represent a documented occurrence of zircon in FANs, offering potential constraints on the timing of farside LMO solidification. The zircons exhibit homogeneous CL structures (Supplementary Fig. 5) and uniform compositions (Supplementary Table 8), indicating that they formed or were reworked during a recrystallisation event. High-precision lead-lead (Pb–Pb) dating via secondary ion mass spectrometer (SIMS) was conducted using a 3 × 3 analytical matrix on a 10 × 10 μm^2^ zircon grain, complemented by single-spot measurements on two smaller zircons (Supplementary Fig. 8). Measured low ^204^Pb count per second (cps) (~0.01–0.04) and ^204^Pb/^206^Pb ratios (on the order of ~10^−5^; Supplementary Table 9) confirm negligible common-Pb contamination. Although the eleven analyses show a small range of dates (from 4410 to 4354 Ma) with a variation of about 1%, the ~50 Ma spread is impossible to represent a prolonged recrystallisation interval, given the textural and compositional uniformity of the analysed zircons. These results likely reflect minor Pb loss during post-crystallisation impact or thermal events, a well-recognised feature observed in ancient lunar zircons^26,27^. Therefore, we interpret the oldest date of 4410 ± 8 Ma (Fig. 3) as representing the zircon formation age.

## Discussion

### Origin of zircon-bearing anorthosite

Multiple petrological lines of evidence indicate high-temperature static recrystallisation within the zircon-bearing anorthosite domain. First, while the host anorthosite clast (565GP01) has coarse-grained anorthitic plagioclase (An_96-98_) typical of FANs (Fig. 2a and Supplementary Fig. 5a, b), the recrystallised domain exhibits fine-grained plagioclase (An_96_) with homogeneous internal structures under CL images (Supplementary Fig. 5b). This microstructural contrast provides evidence for a reworking event that resulted in metamorphic recrystallisation of the smaller plagioclase grains. Second, diagnostic ~120° triple-junction grain boundaries between plagioclase and zircon, as resolved by CL and electron backscatter diffraction (EBSD) images (Fig. 1d and Supplementary Figs. 5b and 6a), imply static recrystallisation caused by a secondary thermal annealing event^1,28^. The weak cumulative misorientation angles (<3°, Supplementary Fig. 6b) and characteristic Raman peaks of the fine plagioclase grains (Supplementary Fig. 4) further indicate that these grains preserve low-strain crystallographic fabrics, consistent with static recrystallisation and minimal post-thermal impact deformation^29^. The presence of tridymite (Fig. 1c), a high-temperature silica polymorph commonly stable above 1000 °C (ref. ^30^), provides a lower bound of temperature attained during the recrystallisation event. This inferred temperature is consistent with the formation conditions of lunar granulites^31^. Although xenomorphic zircon grains may have an impact‑derived origin before recrystallisation, high-temperature metamorphism would be expected to reset their Pb–Pb systematics, causing them to record the timing of the thermal event rather than their original formation age. Therefore, the coexistence of zircon with recrystallised plagioclase, combined with these thermometric constraints, establishes that the zircon-bearing anorthosite likely underwent 4.41 Ga static thermal annealing under granulite-facies conditions.

Two mechanisms have been proposed as potential heat sources for lunar crustal reworking: impact-induced thermal metamorphism^32,33^ and magmatic underplating^31,34^. However, the static recrystallisation texture (Supplementary Figs. 5b and 6a) and low-strain microstructure (<3° misorientation; Supplementary Fig. 6b) analysed in this study are inconsistent with impact-induced deformation. Although the thermal metamorphism of ejecta overlying large impact melt sheets could theoretically produce static recrystallisation^35,36^, the exsolved pyroxenes observed in 565GP01 indicate plutonic, deep-seated crystallisation at ~18 km depth (see burial depth estimation in “Methods”), effectively precluding shallow crustal resetting by impact processes. In addition, the elevated REE concentrations in the recrystallised plagioclase (Fig. 2b and Supplementary Fig. 7) match the chemical signature of anorthositic plagioclase within meteorites metasomatised by KREEP-rich melts^37^. Geochemical modelling further demonstrates that the elevated incompatible element concentrations in the recrystallised plagioclase can be replicated by assimilating ~2.1% KREEP material (see KREEP assimilation modelling in “Methods”; Supplementary Table 10). These observations, coupled with the old recrystallisation age of 4.41 Ga, align with either thermal^31,35^ and chemical^37^ inputs from KREEP-rich Mg-suite magma or with tidal heating^38^ that mobilised KREEP components at depth. The synergistic thermal energy and KREEP signatures delivered by these magmas drove static recrystallisation and facilitated zircon formation within FANs. Thus, the zircon-bearing domain serves as a diagnostic marker of early crustal reworking through KREEP-rich magmatism and metasomatism.

### LMO crystallisation across the Moon

The provenance of individual regolith clasts in the severely gardened lunar surface is inherently complex, especially for anorthosites widely distributed across the lunar highlands. The CE-6 anorthosites could represent materials excavated and redistributed by a wide range of craters, even including nearside large impact basins such as Orientale, which was capable of distributing ancient material globally^39,40^. However, several lines of evidence favour a predominantly local, farside origin for the CE-6 anorthositic clasts. Nearside large impacts would be expected to induce shock and plastic deformation of ballistic ejecta (refs. ^26,41^) and/or reset their crystallisation age^42^, which cannot explain the low-strain microstructure observed in the zircon-bearing anorthosite and the 4.41 Ga age predating the Orientale (∼3.81–3.92 Ga; refs. ^36,43^) and other nearside impacts^44^. Ejecta modelling indicates that at the CE-6 landing site, material from distant ancient basins would be very thin and likely buried by local basalts and ejecta from Copernican craters^45,46^. Remote sensing data reveal abundant pre-Nectarian anorthositic regions within the Apollo basin^47^ and adjacent highland craters such as Vavilov^48^, providing a more probable local source of the anorthosites than nearside ejecta. The location and geological context of the CE-6 landing site, low-strain microtexture, and coherent petrological and geochemical signatures of the clasts consistently suggest a predominantly farside highland origin.

The compositional and petrological similarity of CE-6 farside anorthosites with Apollo nearside FANs indicates the widespread distribution of this lithology across the Moon, challenging models of primordial hemispheric asymmetry arising from LMO differentiation. The near-identical plagioclase anorthite contents (An_95–98_) and REE concentrations and mafic mineral Mg# values (33–70) across both lunar hemispheres (Fig. 2 and Supplementary Figs. 2, 3, and 7) suggest that at least some primary building blocks of the global lunar crust are compositionally comparable. As the mineral chemical compositions in anorthosites are highly sensitive to the specific crystallisation path of the LMO^24,49^, the observed mineralogical uniformity implies that both the nearside and farside experienced a similar late-stage LMO crystallisation process and primary crust flotation. This finding suggests that the crustal geochemical dichotomy observed remotely^5–8^ is unlikely to have resulted from intrinsically different LMO crystallisation paths^9,10^.

A pivotal constraint emerges from clast 565GP01, wherein a recrystallised zircon-bearing domain hosts plagioclase enriched in Th, P, and REEs (Fig. 2b and Supplementary Fig. 7). These metasomatic signatures confirm the presence of KREEP-like material—geochemically linked to the urKREEP layer formed during terminal LMO crystallisation^50^—within the farside crust. This discovery, corroborated by the recent identification of KREEP-component in CE-6 high-Al basalt^51^, regolith^52^, and norite^53^, demonstrates that urKREEP was not restricted to the nearside but was potentially widespread across the Moon. This inference is consistent with the elevated concentrations of thorium within the SPA basin relative to highlands^54^, indicative of the impact of excavation into the urKREEP reservoir^55^. The KREEP signatures in farside samples potentially indicate a laterally continued distribution of final LMO residues from the nearside to the farside of the Moon.

### Implications for the timing of terminal LMO solidification

**Fig. 4:**
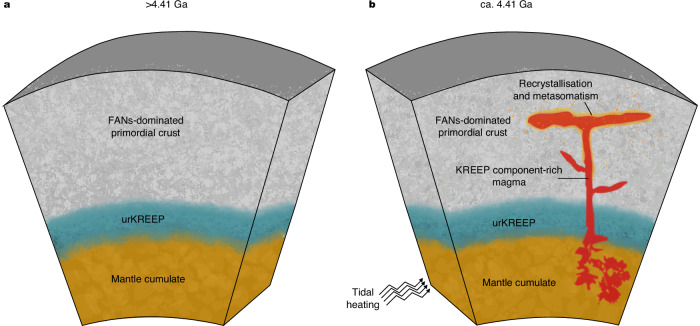
Models of local farside LMO solidification and crustal reworking. **a** Terminal LMO stage (>4.41 Ga): crystallisation of FANs forms the primary farside crust, while residual urKREEP accumulates beneath. **b** Post-LMO stage (ca. 4.41 Ga): regional tidal heating^38^ and/or KREEP-rich magma thermally rework the primary crust, triggering granulite-facies metamorphism and metasomatic enrichment. Not to scale.

Models show that the late-stage LMO crystallisation may have been a prolonged process^56,57^, as also evidenced by the wide range of anorthosite ages (Fig. 3) and its complex reworking history^58,59^. The formation of the urKREEP reservoir marks a specific endpoint of the LMO crystallisation sequence^50^; however, the timing of this terminal solidification has remained a long-standing controversy. Apollo nearside samples yield inconsistent ages for terminal LMO crystallisation, spanning ~160 million years from 4.51 to 4.35 Ga (refs. ^16,26,60–62^). While a recent ^176^Lu–^176^Hf study of Apollo zircons proposed a urKREEP model age of 4429 ± 76 Ma (ref. ^18^), the large uncertainty of this estimate limits its utility. The Pb–Pb zircon date of 4410 ± 8 Ma from CE-6 anorthosites now provides a critical local lower bound for farside LMO solidification. This date, representing the timing of crustal reworking by intrusions enriched in KREEP component, necessitates that both the formation of local farside FANs and the urKREEP reservoir in their source region occurred prior to 4.41 Ga (Fig. 4). Although the LMO solidification may be protracted spatially, this chronology establishes a robust, local snapshot for the cessation of farside LMO solidification and suggests rapid stabilisation of a crustal portion within the earliest 150 million years after Solar System formation.

Our data offer further constraints on the potential synchronicity of late-stage LMO solidification between the two hemispheres. If nearside solidification terminated at ca. 4.35 Ga (refs. ^16,61^), the older age from the farside (>4.41 Ga) could be interpreted as reflecting asymmetric LMO cooling, potentially driven by thermal gradients from Earth’s early radiative heating^9^. Such asymmetric cooling and crystallisation would promote earlier flotation and accumulation of plagioclase on the farside, produce a thicker and more magnesian primary farside crust^17,63^, and leave KREEP-rich residual liquid concentrated under the nearside crust^10,64^. However, the compositional similarity of the CE-6 anorthosites with nearside FANs and the presence of KREEP signatures in farside samples contradict this scenario. The model of LMO termination at ca. 4.35 Ga has also been challenged by a global post-LMO thermal resetting event, such as mantle overturn^65^, the SPA impact^66^, or tidal heating^38^. Alternatively, the comparable primary crust on both lunar hemispheres identified in this study indicates near-synchronous differentiation. The local lower bound for farside LMO solidification constrained here is broadly consistent with the younger limits for nearside terminal LMO crystallisation (4417 ± 6 Ma, ref. ^26^; 4460 ± 31 Ma, ref. ^62^) established from Apollo zircons, further supporting synchronous differentiation. This inference also aligns with the recent Pb–Pb isochron age of 4406.1 ± 3.2 Ma obtained from a lunar meteorite with a high initial Pb composition^67^, indicative of urKREEP formation prior to this time. In addition, the metasomatic assimilation of KREEP-rich magma at 4.41 Ga demonstrates that the early farside mantle retained sufficient thermal energy—potentially supplemented or driven by tidal heating^38^—to sustain post-LMO magmatism, mirroring nearside processes^62,68,69^. Therefore, both the nearside and farside share a similar early differentiation and magmatic history, implying that the crustal dichotomy observed by remote sensing is likely unrelated to primordial asymmetric solidification but instead resulted from post-LMO crustal reworking processes, such as tidal heating or large impacts. Future models for the hemispheric asymmetry need to fit the observed petrological and compositional similarity between nearside and farside primary crusts. The recrystallisation age of 4.41 Ga thus provides a lower bound for the formation of a farside crustal portion, anchoring a key point in the timeline of early lunar evolution.

## Methods

### Sample preparation

The Chang’e-6 samples analysed in this study are lithic clasts separated from two soil samples (CE6C0100JYFM002, 5 g; CE6C0200YJFM001, 5 g). Approximately 2000 clasts were carefully hand-picked using a 200-μm mesh sieve. These clasts were then embedded in epoxy mounts and polished with a grinder. We selected seven representative anorthosite clasts for detailed in situ analyses.

### Scanning electron microscopy analysis and energy-dispersive spectrometry mapping

High-resolution backscattered electron (BSE) imaging and energy-dispersive X-ray spectroscopy (EDS) analyses of the Chang’e-6 anorthosite clasts were conducted using a Zeiss Gemini 450 field-emission scanning electron microscope at the Institute of Geology and Geophysics, Chinese Academy of Sciences (IGGCAS) in Beijing. BSE imaging was carried out at an accelerating voltage of 15 kV, beam current of 2 nA, and working distance of 8.5 mm. The average compositions and modal abundances were derived from quantitative EDS mapping (Oxford Instruments), followed by phase analysis using Aztec 4.3 software. The EDS mapping was conducted under conditions of 20 kV accelerating voltage, 5 nA beam current, 8.5 mm working distance, and 500 μs collection time per pixel. System calibration and analytical validity were verified through repeated measurements of certified reference minerals (Micro-Analysis Consultants Ltd mineral standards), confirming detection limits <0.1 wt% for major elements, with analytical precision (1σ) and accuracy better than 1% and 2%, respectively.

### Cathodoluminescence imaging

Cathodoluminescence (CL) images were obtained using a Gatan Instrument MonoCL3+ system attached to a FEI Nova Nano450 SEM at the Electron Microscopy Laboratory of Peking University. Measurements were performed under an accelerating voltage of 20 kV and a beam current of 120 nA to optimise luminescence signal intensity while minimising sample charging. High-resolution panchromatic CL maps (514 × 514 pixels) were acquired to resolve internal microtextures, including zoning in plagioclase and zircon, as well as grain boundary relationships, which are critical for assessing crystallisation and recrystallisation.

### Electron backscatter diffraction analysis

Crystallographic orientation mapping of plagioclase and zircon in sample 565GP01 was performed using a HITACHI SU5000 scanning electron microscope equipped with an Oxford Instruments Symmetry S3 detector. Prior to analysis, the epoxy-mount sample was polished with 0.05 μm colloidal silica suspension to achieve a strain-free surface. Automated electron backscatter diffraction (EBSD) mapping was acquired in low vacuum mode (30 Pa) across two representative regions (707 × 486 μm^2^ and 1273 × 876 μm^2^) under optimised conditions: 20 kV accelerating voltage, 15 nA beam current, 18.6 mm working distance, 0.15-μm step size, and 2.56 ms exposure time. Raw diffraction patterns were processed using the AZtecCrystal software to generate Euler maps and kernel average misorientation distributions. Noise reduction involved interpolating zero-solution pixels using six-neighbour averaging (within an 8-pixel kernel) and removing wild spikes (>5° deviation from local mean). Grain boundaries were defined as those with misorientations greater than 10°, while subgrain boundaries were characterised by misorientations between 2° and 10°. Grains smaller than 4 pixels were filtered out to enhance detection accuracy. Local misorientation maps were created by setting a lower threshold smooth angle of 0.5° and an upper threshold subgrain angle of 5°.

### Electron probe microanalysis

Mineral major and trace element compositions were conducted using a CAMECA SXFive electron probe microanalyser (EPMA) at IGGCAS. Major element analyses employed five wavelength-dispersive spectrometers under 15 kV accelerating voltage, 20 nA beam current, a focused beam, and 10 s peak counting time per element. Calibration standards used include: albite for Na, MgO for Mg, K-feldspar for Al and K, rhodonite for Si, Ca and Mn, hematite for Fe, Cr_2_O_3_ for Cr, Ni metal for Ni, and rutile for Ti. Detection limits (3σ background variance) range from 0.02 to 0.07 wt%, with accuracy better than 1.5% for major elements (>1.0 wt%) and 10% for minor elements (0.1–1.0 wt%). All data were processed using the phi-rho-Z matrix correction in the CAMECA PeakSight software. The locations of EPMA analysis points are shown in Supplementary Fig. 9.

High-precision analyses of olivine used 20 kV accelerating voltage, 30 nA (major elements) and 900 nA (trace elements) beam currents, and 5 μm beam diameter. Analytical crystals and peak counting time were used as follows: TAP (Mg, 20 s; Si, 20 s; Al, 240 s), LIF (Fe, 20 s; Mn, 120 s), LLIF (Cr, 120 s; Co, 120 s; Ni, 300 s), and LPET (P, 120 s; Ca, 120 s; Ti, 240 s). Detection limits (3σ background variance) are 285 μg g^−^¹ for Mg, 330 μg g^−^¹ for Si, 225 μg g^−^¹ for Fe, 8 μg g^−^¹ for Al, 9 μg g^−^¹ for Ca, 7 μg g^−^¹ for Ti, 18 μg g^−^¹ for Co, 10 μg g^−^¹ for Ni, 26 μg g^−^¹ for Mn, 13 μg g^−^¹ for Cr, and 18 μg g^−^¹ for P. MongOl Sh11-2 olivine was used as monitor standard^70^. Precisions for major elements (Mg, Fe, and Si) and trace elements (Al, Ca, Ti, Co, Ni, Mn, Cr, and P) are better than 1.5% and 10%, respectively.

Major (Na, K, Al, Si, and Ca) and trace element (Mg, Fe, P, and Ti) analyses in plagioclase were performed under 20 kV acceleration voltage, 40 nA (major elements) and 600 nA (trace elements) beam current, and 5 μm spot diameter. Kα characteristic X-rays were selected for all element analyses. Analysing crystals and peak count times were used as follows: Si (TAP, 10 s), Al (TAP, 10 s), Ca (PET, 10 s), Na (TAP, 10 s), K (LPET, 10 s), Mg (TAP, 120 s), Fe (LLIF, 60 s), P (TAP, 60 s), and Ti (LPET, 240 s). Natural and synthetic standards were employed for calibration: albite (Na and Al), K-feldspar (K and Si), periclase (Mg), rhodonite (Ca), apatite (P), rutile (Ti), and hematite (Fe). Detection limits (3σ) are 22 μg g^−^¹ for Mg, 23 μg g^−^¹ for Fe, 25 μg g^−^¹ for P, and 11 μg g^−^¹ for Ti. MGP−1 plagioclase was used as a monitor standard^71^. Trace element measurements show relative deviations within 10% of the reference values.

Zr, Si, Al, Ti, P, and Hf in zircon were analysed at 20 kV using dual-beam currents of 40 nA (Zr, Si, and Hf) and 500 nA (Al, Ti, and P). The following analytical crystals were used: TAP for Si and Al (Kα), PET for Zr (Lα), LLIF for Hf, and LPET for P and Ti (Kα). Calibration standards include zircon (Si and Zr), Hf metal (Hf), rutile (Ti), apatite (P), and Y_3_A_l5_O_12_ (Al). Peak counting times were 10 s for Zr and Si, 30 s for Hf, 240 s for Al, P, and Ti. Detection limits (3σ) are 13 μg g^−^¹ (Al), 12 μg g^−^¹ (Ti), and 26 μg g^−^¹ (P). Cross-validation with standard reference data (GJ−1, Qinghu, Tanz zircons) showed <10% relative deviation for Hf, P, and Ti.

### Laser Raman analysis

Micro-Raman experiments were performed using a WITec alpha 300 R confocal Raman microscope at IGGCAS. A 532 nm diode-pumped solid-state laser was employed for excitation, with beam focusing achieved via a 100× ZEISS EC Epiplan-Neofluar Dic objective (numerical aperture = 0.9). Operational parameters were optimised to detect weak spectral features depending on mineral phase: laser power was adjusted between 3.5 and 15 mW, integration time ranged from 1.5 to 6 s, and spectral accumulations were collected 16–50 times. Spectral acquisition utilised a 300 grooves/mm diffraction grating, providing a resolution of 4.8 cm^−1^. System calibration was verified using the 520.7 cm^−1^ peak of a silicon reference wafer. Raw spectra were processed using WITec Project 5.3 software without baseline correction to preserve low-intensity band signatures. Representative spectra are provided in Supplementary Fig. 4.

### In situ mineral trace-element analysis

Trace element concentrations of plagioclase were determined by laser ablation-inductively coupled plasma-mass spectrometry (LA-ICP-MS) employing an Element XR HR-ICP-MS instrument (Thermo Fisher Scientific, USA) coupled to a 193 nm ArF excimer laser system (Geolas HD, Lambda Physik, Göttingen, Germany) at IGGCAS. Isotopes were measured using a peak-hopping mode and a laser diameter of ca. 44 μm and 5 Hz repetition rate. Detailed instrumental settings followed ref. ^72^. The laser energy density is ~4.0 J cm^−2^. The Element XR is equipped with the so-called “jet-interface” comprising a jet sample cone, an X-version skimmer cone, and a high-capacity vacuum pump (OnTool Booster 150, Asslar, Germany). This leads to a signal enhancement in laser sampling mode by a factor of 3–5, resulting in an improved detection capability. Helium was employed as the ablation gas to improve the transport efficiency of ablated aerosols. ARM-3 reference glass was used for external calibration^73^, NIST SRM 612 and NIST SRM 614^74^, GOR132-G, and GOR128-G^75^ glasses were used for quality control monitoring. Data reduction employed the Iolite software package^76^ with a bulk normalisation algorithm (100 wt% oxide summation) and an in-house data processing scheme. For most trace elements (>10 μg g^−^¹), accuracy is better than ±10% with analytical precision (1σ relative standard deviation) of ±10%. The locations of LA-ICP-MS analysis spots on plagioclase are shown in Supplementary Fig. 10. Measured and recommended reference values for quality control are provided in Supplementary Table 11.

### Pb–Pb isotope analysis of Zr-bearing minerals

The Pb–Pb age of zircons was determined on a CAMECA IMS 1280HR SIMS following the analytical protocol described in ref. ^77^. The mounts were cleaned with a fine (0.25 μm) diamond paste and ethanol to minimise contamination before carbon coating. A primary ^16^O^−^ beam to a size of 3 μm was used at 120 pA and an accelerated potential of −13 kV. Pb isotopes were measured simultaneously in multi-collector mode using four electron multipliers (EMs). A 10 nA^16^ O^−^ primary beam was used for pre-sputtering over 120 seconds before each analysis. Ion images of ^96^Zr_2_^16^O_2_^+^ and Pb isotopes were acquired within a 25 × 25 μm^2^ area to locate the target zircons accurately. NIST SRM610 glass^78^ was used to calibrate the relative yields of different EMs and to assess external reproducibility. Ages were calculated using the Isoplot/Ex programme^79^, with uncertainties for individual analyses reported at the 1σ level, while pooled ^207^Pb/^206^Pb analyses were quoted with 95% confidence interval. Measured compositions were corrected for common Pb using non-radiogenic ^204^Pb. Corrections are sufficiently small to be insensitive to the choice of common Pb composition, and an average of present-day crustal composition^80^ is used for the common Pb, assuming that the common Pb is largely surface contamination introduced during sample preparation. All analysed data are provided in Supplementary Table 9.

### Burial depth estimation

The burial depth (*Z*) of the anorthosite clast 565GP01 was calculated following the framework of ref. ^81^:

1 $$Z={\rm{k}}\times \sqrt{t}$$

where k is a constant (5.2, adopted from ref. ^81^) dependent on thermal diffusivity, *t* is the cooling time that can be solved by the thermal diffusion model. Fe–Mg interdiffusion in pyroxene was leveraged as a cooling time proxy, constrained by compositional gradients within the crystal. One-dimensional Fe–Mg interdiffusion profiles were modelled using Fick’s second law:

2 $$\frac{\partial C}{\partial t}=D\left(\frac{{\partial }^{2}C}{\partial {x}^{2}}\right)$$

where *C* is the element concentration changing with time *t* and distance *x*, and *D* is the temperature-dependent interdiffusion coefficient. The complementary error function (erfc) can be used to get its analytical solution^82^:

3 $$C={C}_{0}+\frac{{C}_{1}-{C}_{0}}{2}\,\left[{\rm{erfc}}\left(\frac{x}{2\sqrt{{Dt}}}\right)\right]$$

where *C*_0_ and *C*_1_ represent the initial and boundary concentrations of the given element, respectively. Monte Carlo simulations were implemented to optimise the diffusion time by minimising the residuals between the modelled and measured concentration profiles. Best-fit solutions were selected when the least misfit values fell within analytical uncertainty bounds.

The Fe–Mg interdiffusion coefficient was adopted from ref. ^83^, incorporating temperature-calibrated parameters. The crystallisation temperatures of the exsolved pyroxene, ranging from 1136 to 1206 °C, were calculated by a pyroxene thermometer^84^ experimentally constructed for mafic magmatic systems. Major element compositions (Si, Ti, Al, Cr, Fe, Mn, Mg, Ca, Na, K) in the pyroxene of 565GP01 were analysed using a standard-based EDS approach with a Zeiss Gemini 450 field-emission scanning electron microscope at IGGCAS. A set of certified reference materials (CRM, Micro-Analysis Consultants Ltd.) was used to calibrate the spectrometer for the elemental analyses: albite for Si, Al, and Na; diopside for Ca; olivine for Mg; almandine for Fe; orthoclase for K; chromite for Cr; rutile for Ti; bustamite for Mn. The detection limit for the major elements was ~0.1 wt%. Pyroxene lamellae with >15 μm width were analysed at 0.5 μm step intervals to obtain detailed compositional variations. 67-point transects across exsolution boundaries ensured robust profile deconvolution (Supplementary Fig. 11). The measured zoning profile indicates a diffusion time of ~12.6 million years with a burial depth of ${18.4\,{\rm{km}}\,}_{-4.8}^{+13.5}$, pointing to a middle level within the lunar crust.

### KREEP assimilation modelling

The proportion of KREEP components assimilated during recrystallisation was quantified using a mass-balance model. Element concentrations were modelled as:

4 $${C}_{i}=\mathop{\sum }\limits_{j\,=\,1}^{n}{f}_{j}\,{c}_{i,\,j}$$

where *C*_*i*_ is the concentration of element *i* in the recrystallised domain, *f*_*j*_ represents the mass fraction of component *j*, and *c*_*i, j*_ denotes the concentration of element *i* in component *j*. La, Ce, Pr, Nd, Sm, and Eu were leveraged as element *i* in the equation to estimate the proportion of assimilated KREEP components due to their diagnostic behaviour. First, light-REEs (LREE) have higher concentrations and precisions than those of heavy-REEs (HREE). Second, the reduced positive Eu anomaly in recrystallised plagioclase contrasts with pristine FANs. For component *j*, two endmembers were defined: (i) the average LREE abundance of plagioclase in clast 565GP01 and (ii) high-K KREEP composition from refs. ^85,86^. A set of values *f*_*j*_ can be obtained using the system of equations for each element *i*. All calculations yield a best-fit solution of *f*_*KREEP*_ = 2.12 ± 0.52% (1σ), reconciling both the LREE enrichment pattern and Eu anomaly attenuation observed in the recrystallised domain. The low LREE abundance of pristine FAN plagioclase renders its compositional influence on the estimated assimilation ratio negligible. All the data involved are shown in Supplementary Table 10.

## Supplementary information

Supplementary Information

Transparent Peer Review file

## Source data

Source Data

## Data Availability

All data generated and analysed in this study are available in Supplementary Tables 1–11. All of the data above for this paper are also deposited in Figshare (10.6084/m9.figshare.31932933). Source data are provided with this paper.
