# Supplementary material for: Chang’e-6 farside anorthosites indicate hemispherically comparable magma ocean solidification
Source: Nat Commun. 2026 May 15;17:6467. doi: 10.1038/s41467-026-73258-y (PMC13376548; doi:10.1038/s41467-026-73258-y)
Supplement: Supplementary file 1 — Supplementary Information [file 41467_2026_73258_MOESM1_ESM.pdf]

Supplementary Information for

Chang’e-6 farside anorthosites indicate hemispherically comparable magma ocean solidification

This file includes:

1. Supplementary Figures 1-11

- ✧ Supplementary Fig. 1 | Backscattered electron images of representative CE-6 anorthosite clasts.
- ✧ Supplementary Fig. 2 | Lithological classification of CE-6 anorthosite clasts compared to Apollo FANs.
- ✧ Supplementary Fig. 3 | Comparative mineral chemistry of CE-6 anorthosites and nearside Apollo FANs.
- ✧ Supplementary Fig. 4 | Representative Raman spectral of plagioclase, tridymite, and zircon in the recrystallised domain of 565GP01.
- ✧ Supplementary Fig. 5 | Backscattered electron (BSE) and Cathodoluminescence (CL) images of the zircon-bearing recrystallisation domain.
- ✧ Supplementary Fig. 6 | Electron backscattered diffraction image of recrystallisation domain in clast 565GP01.
- ✧ Supplementary Fig. 7 | Plagioclase geochemical signatures of CE-6 anorthosites compared to Apollo crustal lithologies.
- ✧ Supplementary Fig. 8 | *In situ* SIMS Pb-Pb analytical positions on zircon grains.
- ✧ Supplementary Fig. 9 | Backscattered electron images of seven representative CE-6 anorthosite clasts with EPMA analytical positions.
- ✧ Supplementary Fig. 10 | Backscattered electron images marked with LA-ICP-MS analytical positions of CE-6 anorthosite clasts.
- ✧ Supplementary Fig. 11 | Diffusion zoning of an exsolved pyroxene in 565GP01.

2. Supplementary Tables 1-11

- ✧ Supplementary Table 1 | Modal mineralogy of CE-6 anorthosite clasts analysed in this study.
- ✧ Supplementary Table 2 | Average mineral major element compositions (wt%) of CE-6 anorthosite clasts.
- ✧ Supplementary Table 3 | All mineral major element compositions (wt%) of CE-6 anorthosite clasts.
- ✧ Supplementary Table 4 | Olivine major and trace element compositions measured by EPMA.
- ✧ Supplementary Table 5 | Average plagioclase trace element compositions ($\mu g\ g^{-1}$) of CE-6 anorthosite clasts.

- ✧ Supplementary Table 6 | Plagioclase trace element compositions (μg g^{-1}) analysed by LA-ICP-MS.
- ✧ Supplementary Table 7 | Plagioclase major and trace element compositions measured by EPMA.
- ✧ Supplementary Table 8 | Zircon compositions measured by EPMA.
- ✧ Supplementary Table 9 | Pb-Pb isotope data of zircon within CE-6 anorthosite clast.
- ✧ Supplementary Table 10 | KREEP assimilation ratio calculated based on plagioclase REE concentrations and high-K KREEP.
- ✧ Supplementary Table 11 | Measured and recommended reference values for *in situ* trace element analyses.

3. Supplementary Reference

1. Supplementary Figures

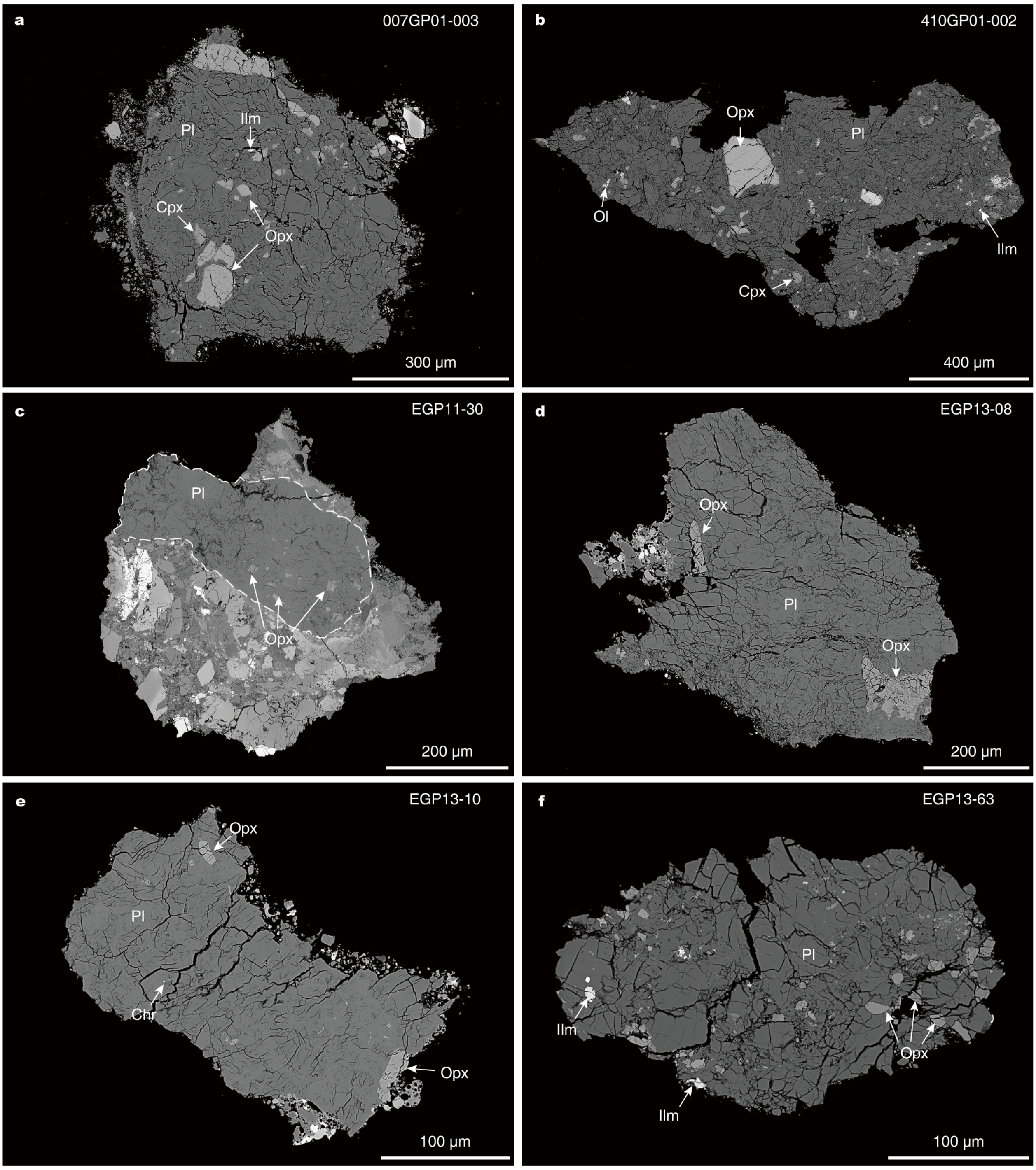

Supplementary Fig. 1 | Backscattered electron images of representative CE-6 anorthosite clasts. **a,** Coarse-grained anorthosite showing poikilitic texture, with ilmenite (Ilm), orthopyroxene (Opx), and clinopyroxene (Cpx) associated with plagioclase (Pl). **b,** Fractured olivine (Ol), pyroxene, and ilmenite intergrown with plagioclase. **c,** Anorthosite clast within breccia containing minor orthopyroxene. **d,** Poikilitic orthopyroxene enclosed in fractured plagioclase. **e,** Plagioclase hosting orthopyroxene and chromium-spinel (Cr-Spl) within an anorthosite clast. **f,** Fine-grained orthopyroxene and ilmenite enclosed in fractured plagioclase.

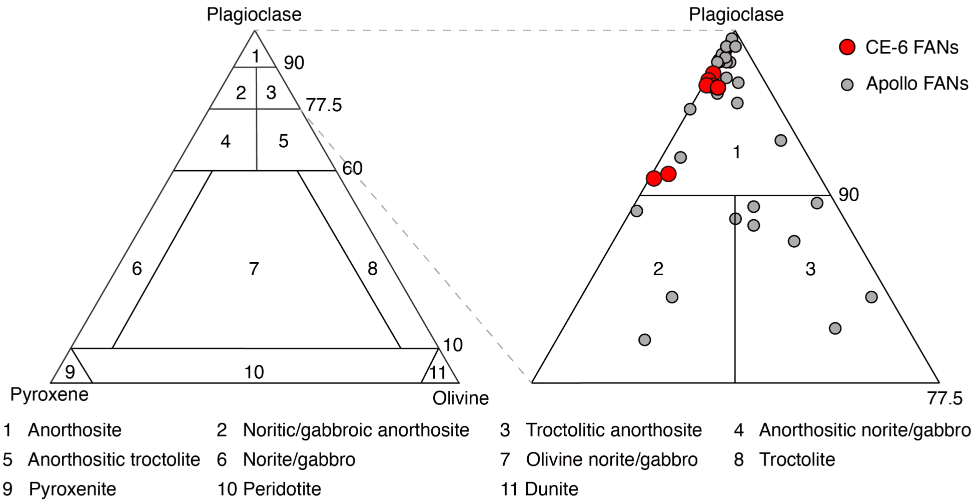

Supplementary Fig. 2 | Lithological classification of CE-6 anorthosite clasts compared to Apollo FANs. Plagioclase-pyroxene-olivine ternary diagram (vol%) illustrating the mineral modal abundances of CE-6 anorthosites (red circles; n = 7) and Apollo FANs (grey circles; data were listed in Source data). All CE-6 data, as presented in Supplementary Table 1, were derived from quantitative EDS mapping.

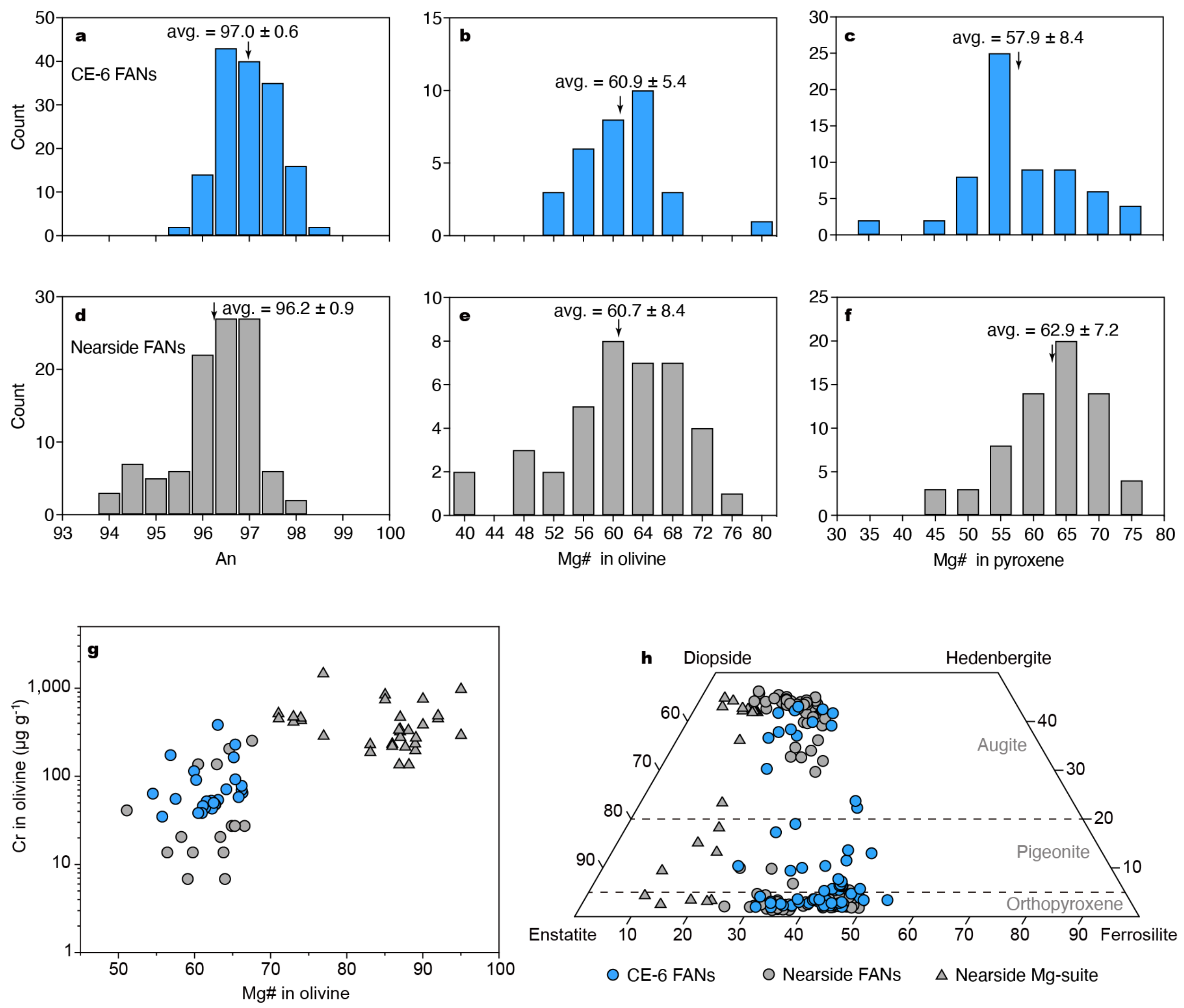

Supplementary Fig. 3 | Comparative mineral chemistry of CE-6 anorthosites and nearside Apollo FANs. a, d, Histograms showing frequency distributions of plagioclase anorthite content (An). **b, e,** Mg# values of olivine. **c, f,** Mg# values of pyroxene. Uncertainties reported as ± values represent 1σ. **g,** Cr vs. Mg# in olivine. **h,** Composition of pyroxene. CE-6 anorthosites and nearside Apollo FANs exhibit similar mineral compositions, including plagioclase An contents, mafic mineral Mg# values, and olivine Cr concentrations. Apollo data were listed in Source Data.

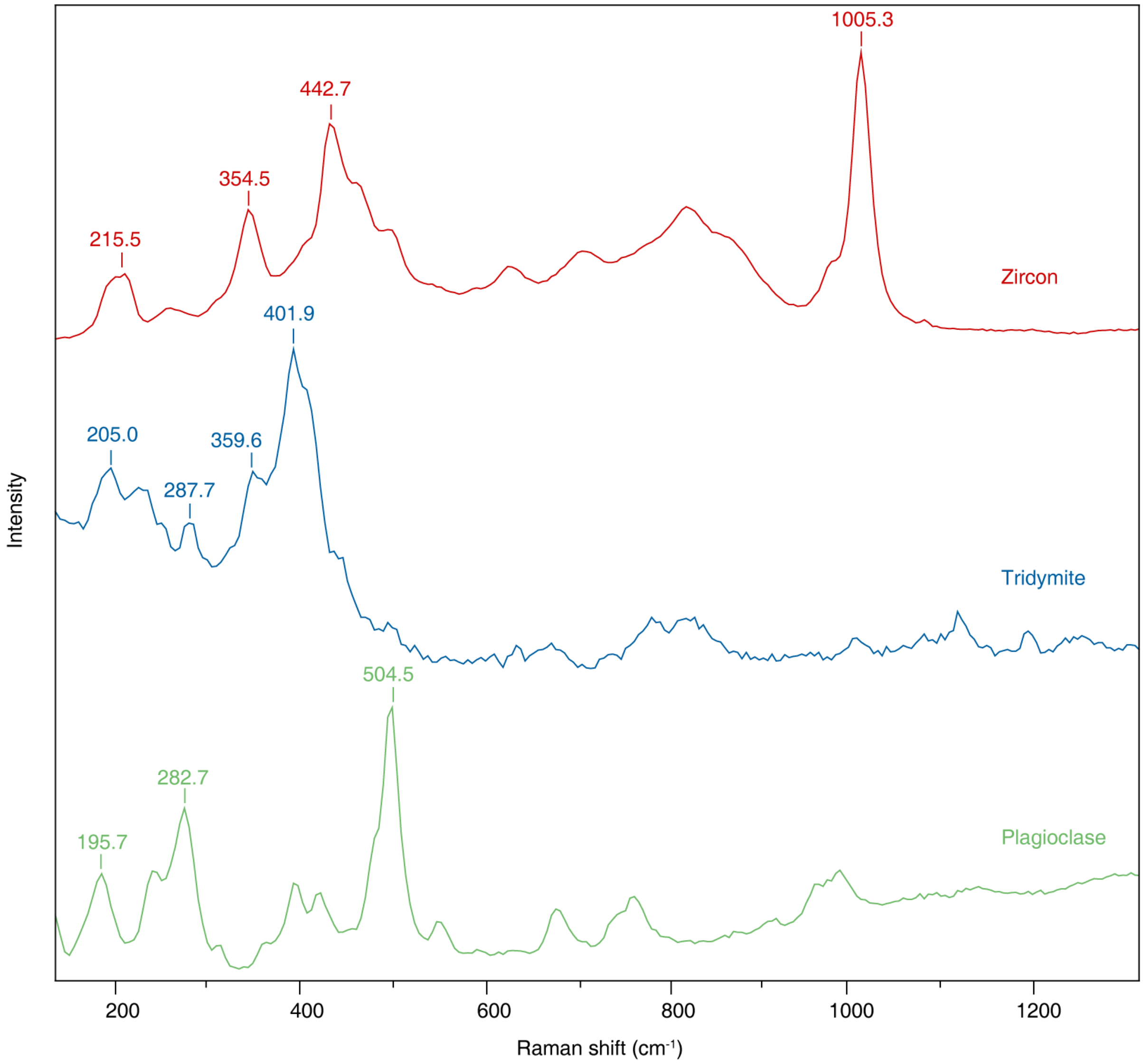

Supplementary Fig. 4 | Representative Raman spectral of plagioclase, tridymite, and zircon in the recrystallised domain of 565GP01. The characteristic Raman peaks of each mineral are labelled. No evidence of shock-induced amorphization (eg. broad features or absent characteristic peaks) is observed, confirming that crystalline structures are preserved.

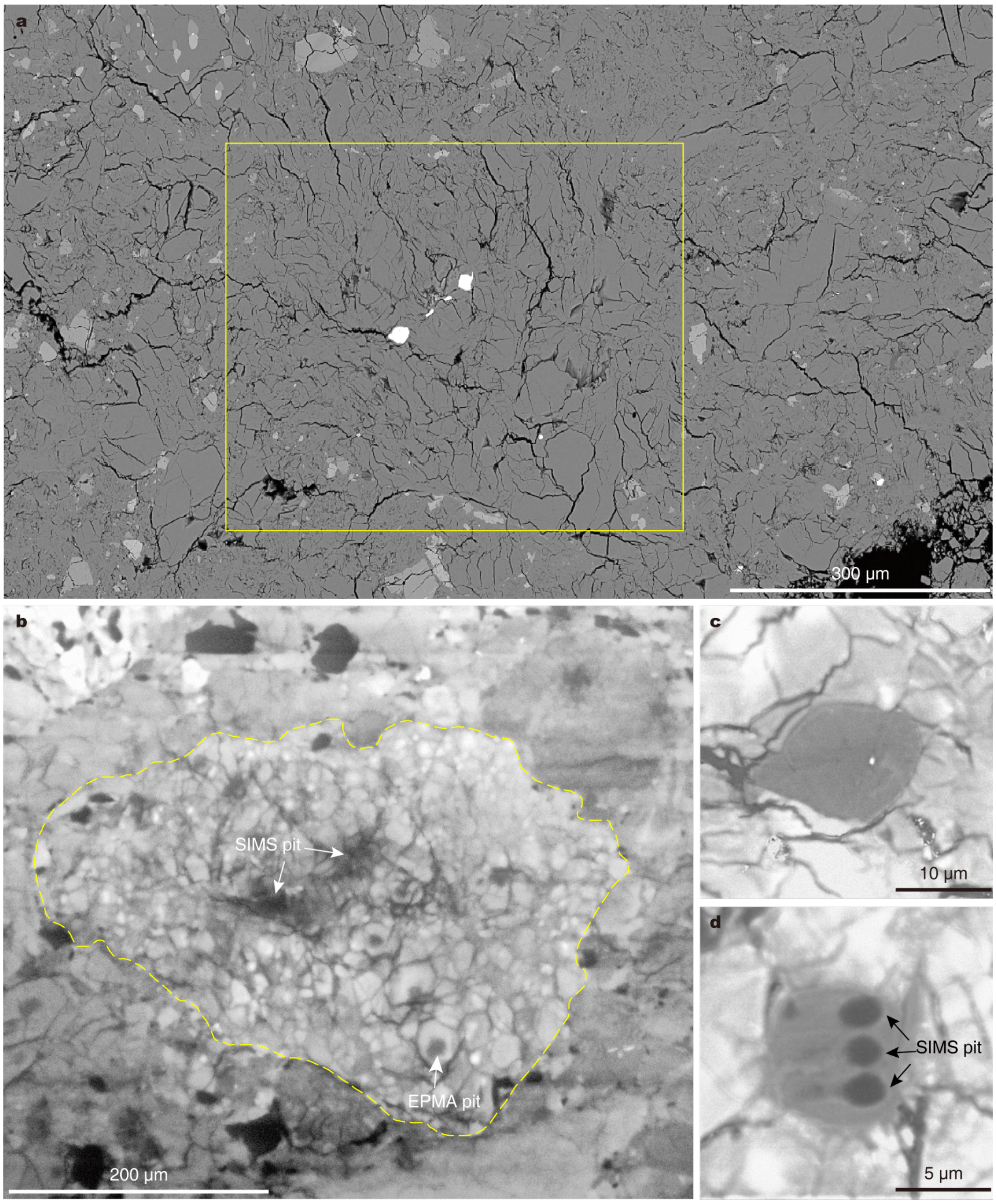

Supplementary Fig. 5 | Backscattered electron (BSE) and Cathodoluminescence (CL) images of the zircon-bearing recrystallisation domain. a, BSE image of a zircon-bearing area within anorthosite clast 565GP01. Plagioclase grains within the area have similar contracts. **b,** CL image of the boxed region in **a** (yellow rectangle), showing the recrystallisation domain within anorthosite. Plagioclase grains display straight crystal boundaries with ~120° triple junctions. **d, e,** CL images of two >10 µm zircons exhibiting homogeneous luminescence characteristics.

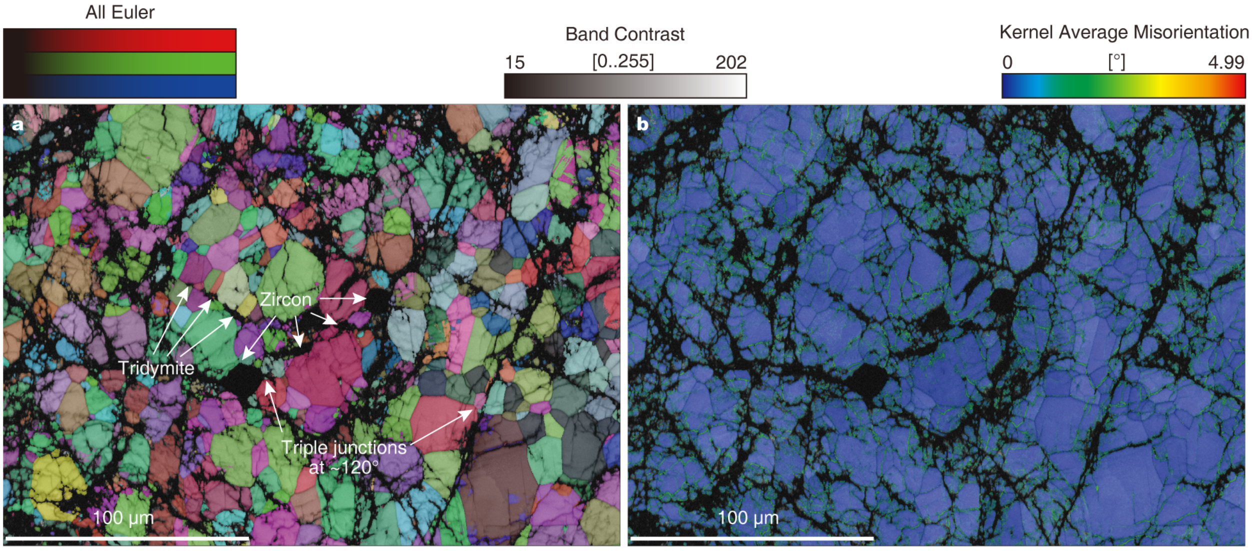

Supplementary Fig. 6 | Electron backscattered diffraction image of recrystallisation domain in clast 565GP01. a, Euler image showing diagnostic ~120° triple-junction grain boundaries between plagioclase and zircon. **b,** Kernel average misorientation image exhibiting low-strain microstructure (<3° misorientation) for plagioclase, indicative of static recrystallisation.

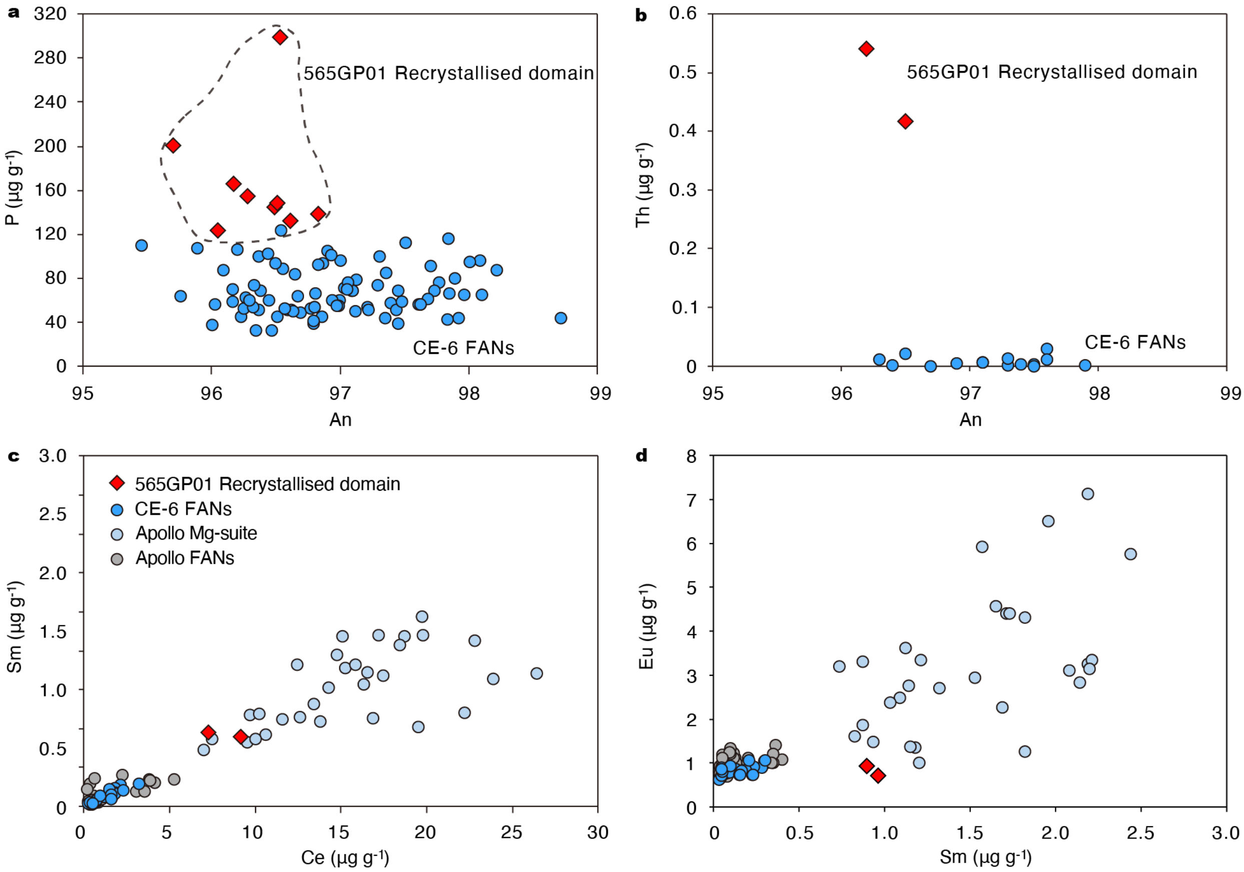

Supplementary Fig. 7 | Plagioclase geochemical signatures of CE-6 anorthosites compared to Apollo crustal lithologies. a, Th vs. An; **b,** P vs. An; **c,** Sm vs. Ce; **d,** Eu vs. Sm. Compared to pristine plagioclase in CE-6 and Apollo FANs, the recrystallised plagioclase in clast 565GP01 exhibits elevated Th, P, and REE concentrations, converging toward plagioclase compositions of Apollo Mg-suite. These anomalies support assimilation of KREEP-bearing magma.

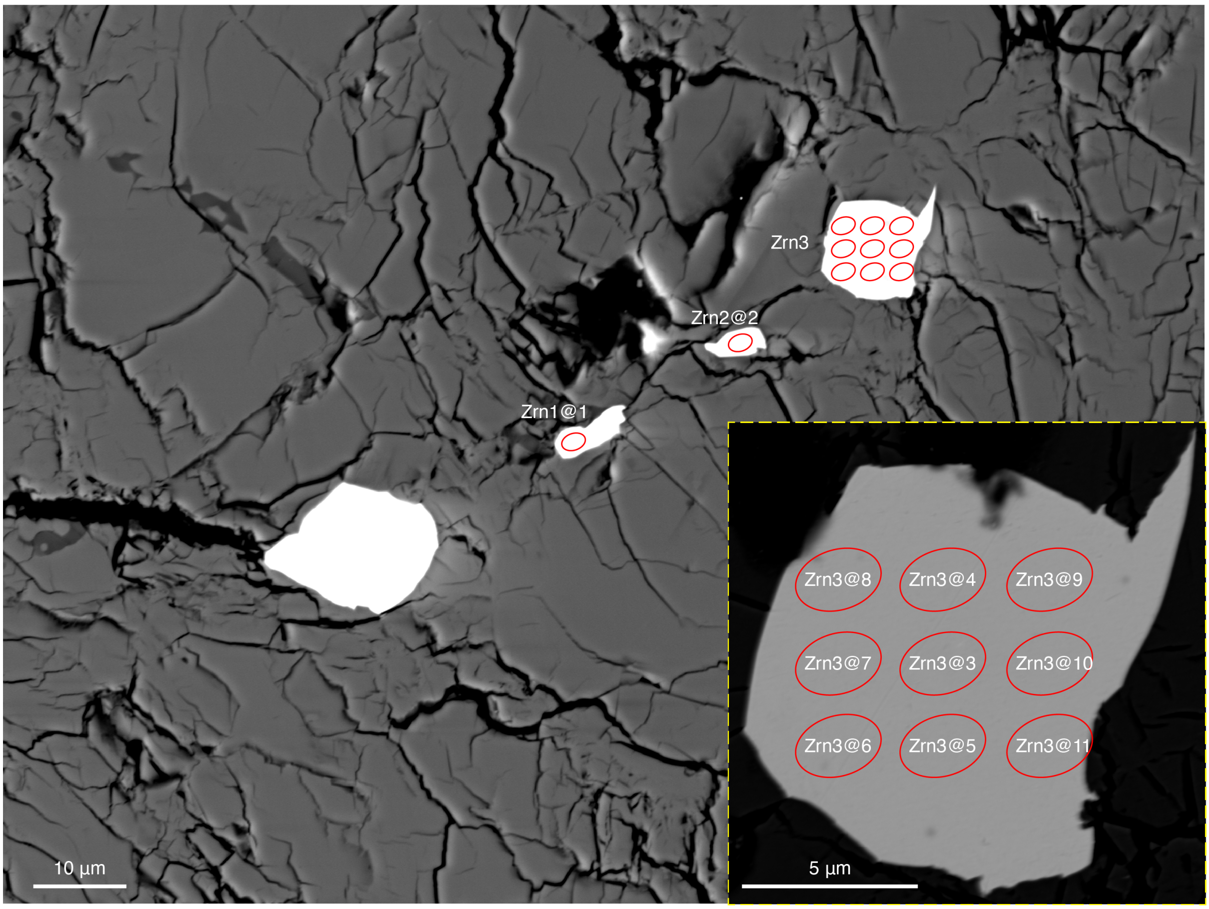

Supplementary Fig. 8 | *In situ* SIMS Pb-Pb analytical positions on zircon grains. A total of eleven SIMS Pb-Pb analyses were conducted on three zircon grains, with a 3 × 3 matrix analysis set up on a 10 × 10 μm^2 zircon grain (labelled as Zrn3). Corresponding data are provided in Supplementary Table 9. Zrn, zircon.

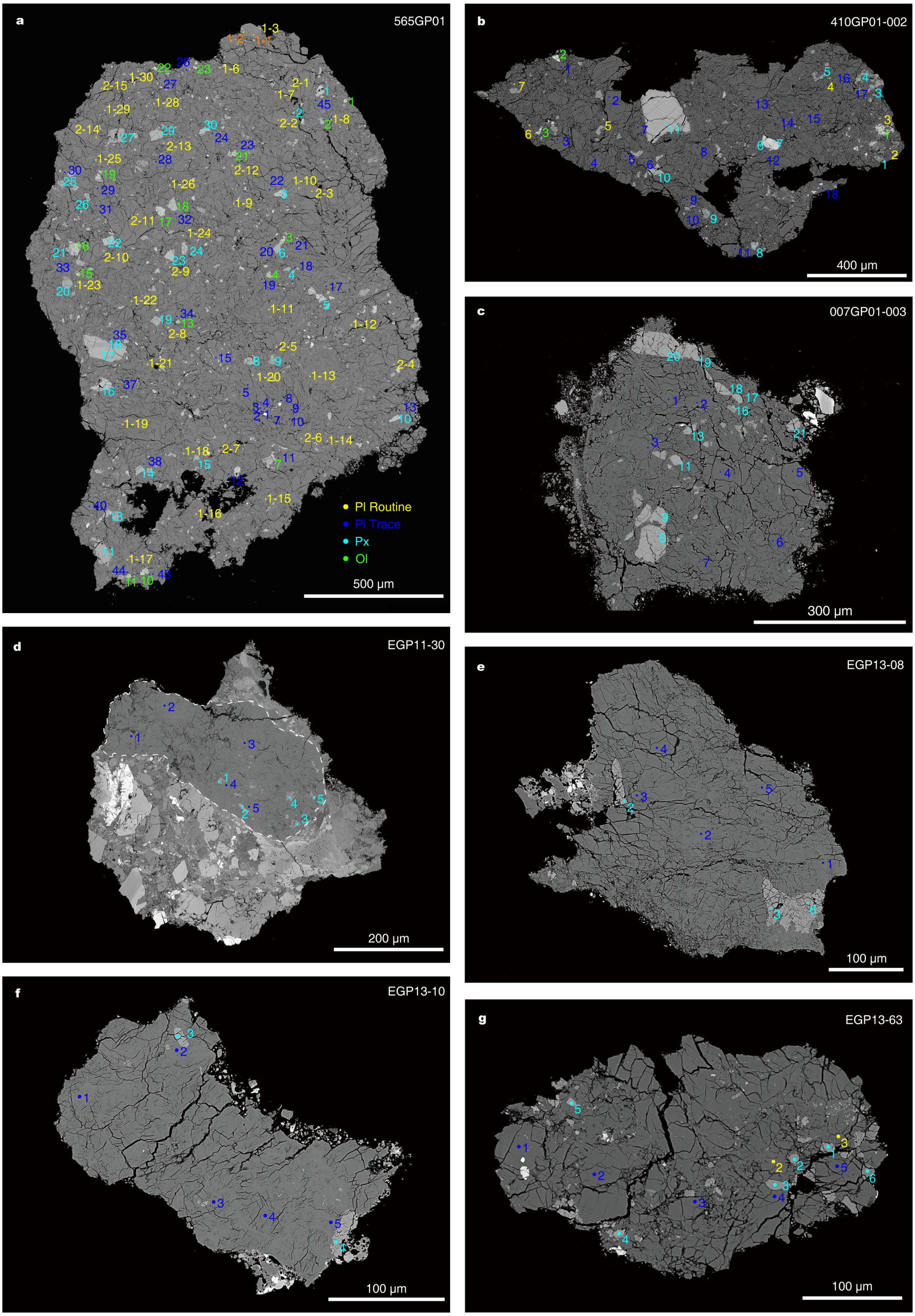

Supplementary Fig. 9 | Backscattered electron images of seven representative CE-6 anorthosite clasts with EPMA analytical positions. The locations of the EPMA points are marked with circles for plagioclase routine in yellow, plagioclase trace in dark blue, pyroxene in light blue, and olivine in green. Corresponding data are provided in Supplementary Tables 2-5, 7. Ol, olivine; Pl, plagioclase; Px, pyroxene.

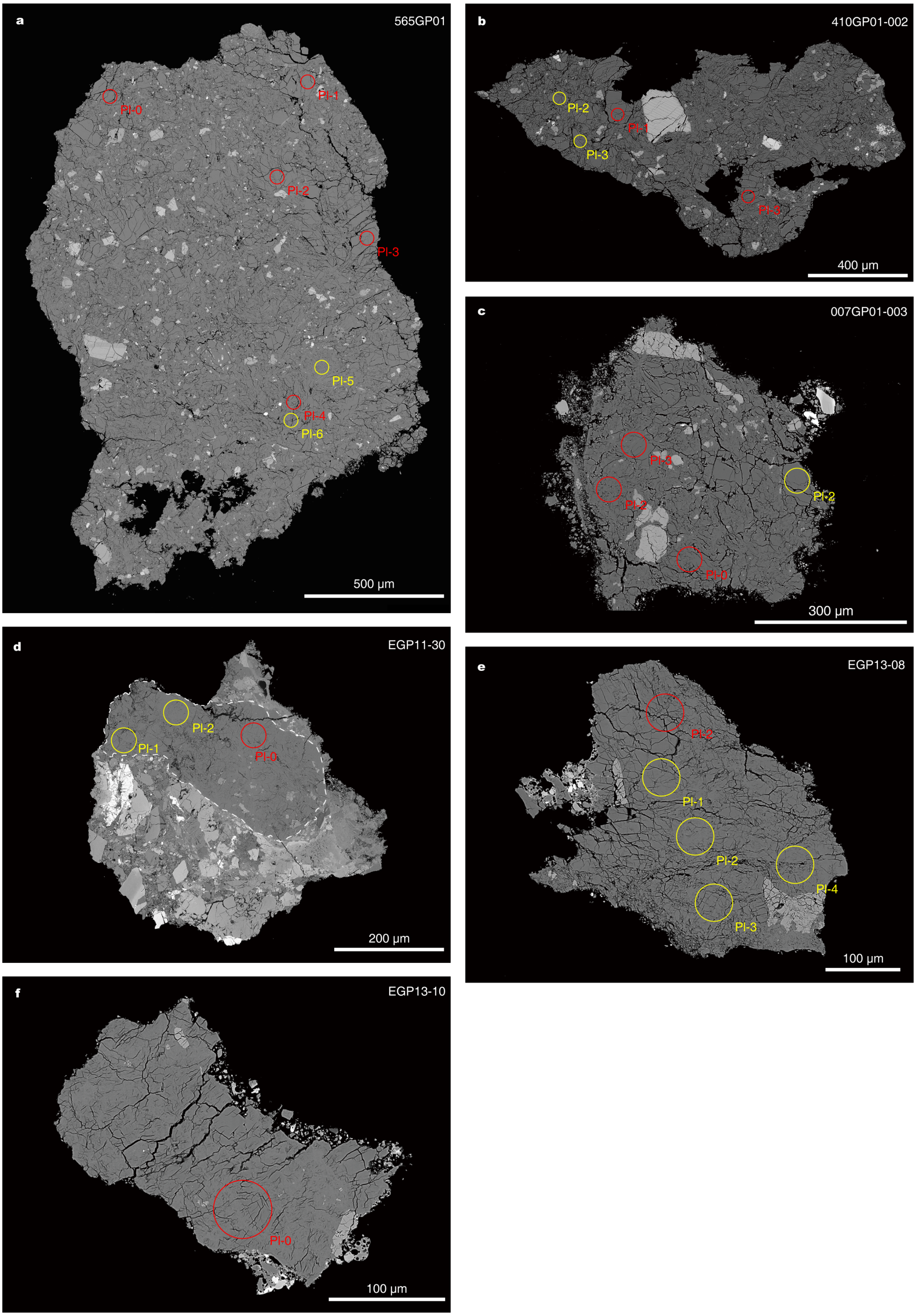

Supplementary Fig. 10 | Backscattered electron images marked with LA-ICP-MS analytical positions of CE-6 anorthosite clasts. Corresponding data are provided in Supplementary Table 6. Pl, plagioclase.

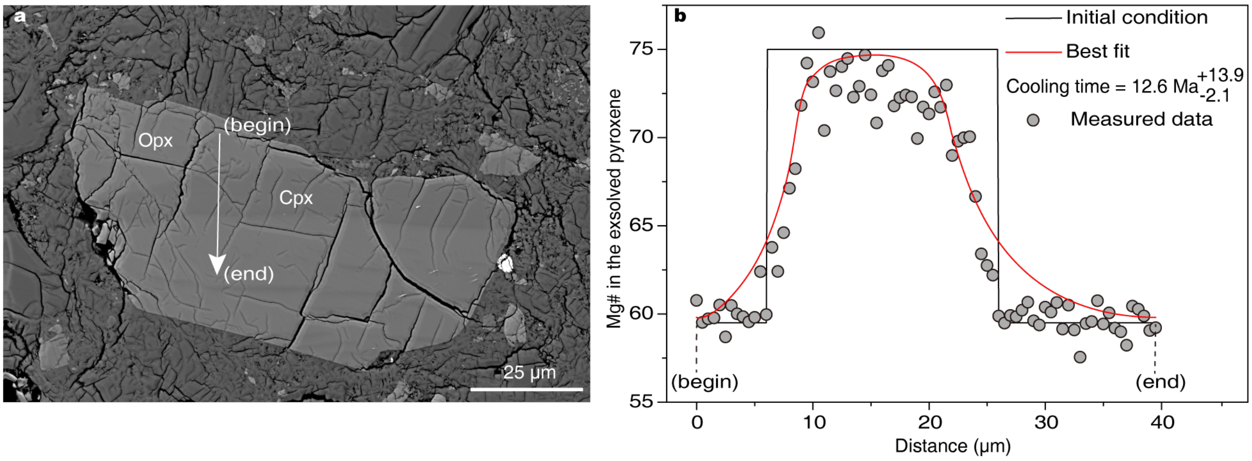

Supplementary Fig. 11 | Diffusion zoning of an exsolved pyroxene in 565GP01. a, Backscattered electron image showing clinopyroxene (Cpx) and orthopyroxene (Opx) exsolution. **b,** Measured Fe-Mg interdiffusion profile (dot circles) fitted to a calculated diffusion curve (red curve). The white arrow in **a**. indicates EDS traverse. The black line in **b** represents the initial profile shape before interdiffusion.

Supplementary Table 1 | Modal mineralogy of CE-6 anorthosite clasts analysed in this study.

Clast No.	Size (mm)	Pl	LCP	HCP	Ol	Ilm	Tro	Cr-Spl	Silica	Other
565GP01	1.5 × 1.1	96.3	2.1	0.7	0.8	trace	trace	trace	trace	trace
007GP01	0.7 × 0.6	90.6	8.6	0.5	0.3	-	-	-	-	-
410GP01	1.1 × 0.6	90.8	7.2	1.0	0.9	trace	-	-	-	trace
EGP11-30	0.4 × 0.2	97.5	2.5	-	-	-	-	-	-	-
EGP13-08	0.6 × 0.4	96.7	3.3	-	-	-	-	-	-	-
EGP13-10	0.2 × 0.1	96.5	3.5	-	-	-	-	-	-	-
EGP13-63	0.3 × 0.2	96.5	3.4	-	-	trace	-	-	-	-

Mineral modal abundance (vol%) was determined by automated image analysis of scanning electron microscope energy-dispersive spectroscopy data, with an estimated uncertainty of ±5%. Modal abundance labelled as 'trace' are less than 0.1%. Pl, plagioclase; LCP, low-Ca pyroxene; HCP, high-Ca pyroxene; Ol, olivine; Ilm, ilmenite; Tro, troilite; Cr-Spl, chromium-spinel.

Supplementary Table 2 | Average mineral major element compositions (wt%) of CE-6 anorthosite clasts.

Clast No.	Mineral	n	SiO_2	TiO_2	Al_2O_3	Cr_2O_3	FeO	MnO	MgO	CaO	Na_2O	K_2O	NiO	Total	Aver. An/Mg#	1SD	Compositional ranges
	Pl	42	43.0	0.01	36.5	0.01	0.19	-	0.08	19.5	0.31	0.01	-	99.6	97.2	0.5	$An_{96.0–98.4}$
565GP01	LCP	23	51.3	0.3	0.58	0.18	25.1	0.44	19.8	1.82	0.01	0	0.01	99.5	58.4	5.7	$En_{47.5–65.1}Wo_{1.9–5.7}9Fs_{29.6–48.5}$
	HCP	4	50.5	0.94	1.65	0.48	12.3	0.26	13.8	19.2	0.05	0	0.01	99.2	66.5	4.0	$En_{34.8–42.5}Wo_{37.1–42.5}Fs_{17.9–22.7}$
	Ol	18	36.0	0.02	0.07	0.01	33.8	0.37	29.0	0.32	0.01	0	0.01	99.6	60.4	3.9	$Fo_{54.5–65.4}$
	Pl	7	43.1	0.01	35.9	0.01	0.14	0.01	0.04	19.6	0.28	0.01	0.01	99.1	97.4	0.2	$An_{97.1–97.6}$
007GP01	LCP	10	51.1	0.31	0.52	0.16	26.7	0.47	17.2	3.03	0.01	bdl	0.01	99.4	53.4	0.6	$En_{49.1–51.5}Wo_{5.4–7.7}Fs_{42.8–44.3}$
	HCP	1	51.2	0.66	0.97	0.38	13.6	0.28	13.0	19.1	0.01	0	0.01	99.2	63.1	0.0	$En_{37.9}Wo_{39.9}Fs_{22.2}$
	Pl	2	43.2	0.02	35.1	0	0.27	-	0.09	19.6	0.4	0.03	-	98.7	96.3	0.5	$An_{95.9–96.6}$
410GP01	LCP	9	50.9	0.56	0.92	0.32	23.6	0.41	16.4	5.93	0.03	bdl	0.01	99.1	55.4	13.5	$En_{27.9–65.9}Wo_{9.4–17.3}Fs_{23.8–56.9}$
	HCP	1	51.6	1.01	1.56	0.45	9.35	0.22	14.9	20.1	0.01	bdl	0	99.2	74.0	0.0	$En_{43.1}Wo_{41.8}Fs_{15.2}$
	Ol	4	34.9	0.06	0.01	0.02	38.1	0.39	25.0	0.23	0.03	-	0.01	98.7	53.9	0.9	$Fo_{52.8–54.9}$
EGP11-30	LCP	1	50.8	0.17	0.28	0.11	28.2	0.52	14.1	6.21	0.02	0	bdl	100.4	47.0	0.0	$En_{40.9}Wo_{13.0}Fs_{46.1}$
	HCP	4	51.7	0.34	0.55	0.17	19.6	0.39	12.6	15.3	0.01	0	0.01	100.6	53.3	4.2	$En_{33.4–38.8}Wo_{22.3–41.7}Fs_{24.9–38.9}$
EGP13-08	HCP	3	52.6	1.3	1.5	0.42	10.8	0.22	16.7	17.0	0.06	0	0.01	100.5	73.3	1.3	$En_{44.9–50.8}Wo_{30.3–37.8}Fs_{17.2–18.9}$
EGP13-10	LCP	2	53.7	0.27	0.45	0.17	20.8	0.39	22.4	1.45	bdl	0	0	99.7	65.8	0.0	$En_{63.7–63.9}Wo_{2.8–3.1}Fs_{33.2–33.3}$
	Pl	2	44.4	0.02	35.9	-	0.33	-	0.08	19.4	0.38	0.01	-	100.5	96.5	0.4	$An_{96.3–96.8}$
EGP13-63	LCP	7	52.6	0.19	0.53	0.06	27.7	0.47	17.8	1.56	0.03	bdl	0.01	100.9	53.4	4.8	$En_{42.9–55}Wo_{2.2–5.3}Fs_{41.6–53.7}$
	HCP	1	52.0	0.66	1.27	0.32	11.2	0.21	13.5	20.8	0.06	0	0	100.1	68.3	0.0	$En_{38.9}Wo_{43.1}Fs_{18.1}$

Completed mineral major compositional data are provided in Supplementary Table 3. Pl, plagioclase; LCP, low-Ca pyroxene; HCP, high-Ca pyroxene; Ol, olivine; An, anorthosite; En, enstatite; Wo, wollastonite; Fs, ferrosilite; Fo, forsterite.

Supplementary Table 3 | All mineral major element compositions (wt%) of CE-6 anorthosite clasts.

Spot No.	SiO_2	TiO_2	Al_2O_3	Cr_2O_3	FeO	MnO	MgO	CaO	Na_2O	K_2O	NiO	Total	An	Mg#	En	Wo	Fs
**007GP01**																	
007GP01-003-px-08	51.37	0.27	0.62	0.12	26.78	0.45	17.07	3.02	bdl	bdl	0.02	99.7		53.2	49.8	6.3	43.9
007GP01-003-px-09	51.41	0.31	0.35	0.15	26.95	0.45	16.92	2.95	0.00	bdl	bdl	99.5		52.8	49.5	6.2	44.3
007GP01-003-px-011	51.22	0.29	1.28	0.15	26.48	0.48	16.70	3.41	0.00	bdl	0.00	100.0		52.9	49.1	7.2	43.7
007GP01-003-px-013	50.94	0.36	0.40	0.18	26.85	0.47	16.89	2.99	bdl	bdl	0.01	99.1		52.9	49.5	6.3	44.2
007GP01-003-px-016	51.18	0.27	0.41	0.13	26.74	0.51	16.98	3.15	0.03	bdl	bdl	99.4		53.1	49.6	6.6	43.8
007GP01-003-px-017	51.23	0.28	0.43	0.14	26.87	0.49	17.24	3.05	bdl	bdl	0.02	99.8		53.4	49.9	6.4	43.7
007GP01-003-px-018	50.64	0.30	0.37	0.16	26.86	0.50	17.23	2.63	0.01	bdl	0.01	98.7		53.4	50.4	5.5	44.1
007GP01-003-px-019	50.72	0.30	0.45	0.19	26.62	0.46	17.63	2.59	0.01	bdl	bdl	99.0		54.1	51.2	5.4	43.4
007GP01-003-px-020	51.14	0.36	0.40	0.17	26.44	0.44	17.84	2.76	0.02	bdl	bdl	99.6		54.6	51.5	5.7	42.8
007GP01-003-px-021	50.71	0.39	0.48	0.18	26.42	0.45	17.12	3.72	bdl	bdl	bdl	99.5		53.6	49.4	7.7	42.8
007GP01-003-pl-01	43.30	0.01	36.12	0.01	0.12	0.00	0.06	19.58	0.26	0.01	bdl	99.5	97.6				
007GP01-003-pl-02	42.79	0.01	35.80	bdl	0.15	0.01	0.03	19.54	0.28	0.01	bdl	98.6	97.5				
007GP01-003-pl-03	43.06	0.02	36.02	0.01	0.14	0.01	0.02	19.62	0.29	0.01	0.02	99.2	97.3				
007GP01-003-pl-04	43.12	0.01	35.91	0.00	0.13	bdl	0.06	19.60	0.26	0.01	bdl	99.1	97.6				
007GP01-003-pl-05	43.42	0.02	35.84	0.01	0.19	0.03	0.03	19.66	0.31	0.01	0.00	99.5	97.1				
007GP01-003-pl-06	42.73	0.01	35.61	0.00	0.11	0.02	0.01	19.52	0.28	0.02	bdl	98.3	97.4				
007GP01-003-pl-07	43.10	0.02	35.94	bdl	0.15	bdl	0.04	19.61	0.27	0.02	bdl	99.1	97.4				
**410GP01**																	
410GP01-002-ol-1	35.02	0.09	bdl	0.02	37.19	0.37	25.37	0.20	0.03	bdl	0.00	98.3		54.9			
410GP01-002-ol-2	34.20	0.04	0.02	0.04	38.40	0.39	24.11	0.20	0.02	bdl	0.02	97.4		52.8			
410GP01-002-ol-3	34.87	0.04	0.05	0.00	38.27	0.39	24.60	0.21	0.03	bdl	0.02	98.5		53.4			
410GP01-002-ol-4	35.48	0.07	0.00	0.01	38.38	0.42	25.73	0.29	0.03	bdl	bdl	100.4		54.4			
410GP01-002-px-01	53.16	0.26	1.56	0.64	14.92	0.27	23.24	5.08	bdl	bdl	0.01	99.2		73.5	65.9	10.4	23.8

Spot No.	SiO_2	TiO_2	Al_2O_3	Cr_2O_3	FeO	MnO	MgO	CaO	Na_2O	K_2O	NiO	Total	An	Mg#	En	Wo	Fs
410GP01-002-px-03	52.36	0.52	0.81	0.29	20.60	0.37	19.67	4.51	0.02	bdl	bdl	99.1		63.0	57.0	9.4	33.5
410GP01-002-px-04	48.23	0.64	0.66	0.11	32.69	0.52	9.00	6.83	bdl	bdl	0.01	98.7		32.9	27.9	15.2	56.9
410GP01-002-px-05	48.54	0.58	0.85	0.16	31.05	0.49	9.86	6.98	0.04	bdl	0.00	98.5		36.1	30.5	15.5	54.0
410GP01-002-px-06	51.03	0.73	0.81	0.35	24.18	0.39	17.45	5.00	0.00	bdl	bdl	99.9		56.3	50.4	10.4	39.2
410GP01-002-px-07	51.59	1.01	1.56	0.45	9.35	0.22	14.92	20.11	0.01	bdl	0.00	99.2		74.0	43.1	41.8	15.2
410GP01-002-px-08	51.00	0.47	0.83	0.37	25.40	0.46	15.49	5.39	0.09	bdl	0.03	99.5		52.1	46.1	11.5	42.4
410GP01-002-px-09	50.51	0.44	0.85	0.31	24.95	0.44	15.07	6.39	0.02	bdl	bdl	99.0		51.8	44.7	13.6	41.6
410GP01-002-px-10	51.41	0.61	0.78	0.27	21.70	0.37	18.85	4.80	0.04	bdl	0.02	98.8		60.8	54.7	10.0	35.3
410GP01-002-px-11	52.07	0.74	1.17	0.36	16.72	0.36	19.36	8.37	0.01	bdl	0.01	99.2		67.4	55.7	17.3	27.0
410GP01-002-pl-2	43.49	0.03	34.64	bdl	0.29		0.10	18.82	0.36	0.04		97.8	96.4				
410GP01-002-pl-3	43.07	0.02	34.42	0.01	0.24		0.04	19.30	0.38	0.03		97.5	96.4				
410GP01-002-pl-4	42.72	0.03	34.28	bdl	0.18		0.09	19.20	0.35	0.04		96.9	96.6				
410GP01-002-pl-5	43.25	0.02	34.95	0.00	0.25		0.13	19.56	0.44	0.03		98.6	95.9				
410GP01-002-pl-6	43.04	0.02	35.27	bdl	0.30		0.06	19.68	0.37	0.02		98.8	96.6				
410GP01-002-pl-7	42.84	0.01	34.67	bdl	0.16		0.05	19.32	0.38	0.04		97.5	96.3				
**565GP01**																	
565GP01-ol-1	35.19	0.07	0.11	0.03	36.31	0.40	26.84	0.44	0.00	0.00	0.01	99.4		56.8			
565GP01-ol-2	36.76	0.07	0.06	0.03	30.27	0.32	32.02	0.33	0.00	0.00	0.01	99.9		65.3			
565GP01-ol-3	36.64	0.01	0.15	0.02	34.25	0.38	28.76	0.39	0.00	0.00	0.01	100.6		59.9			
565GP01-ol-4	36.49	0.01	0.04	0.06	31.70	0.35	30.35	0.41	0.01	0.00	0.01	99.4		63.1			
565GP01-ol-7	36.17	0.02	0.05	0.01	35.93	0.41	27.29	0.33	0.00	0.00	0.01	100.2		57.5			
565GP01-ol-10	35.44	0.02	0.04	0.01	37.08	0.39	26.22	0.33	0.00	0.00	0.00	99.5		55.8			
565GP01-ol-11	35.70	0.03	0.29	0.00	35.32	0.38	27.47	0.44	0.00	0.00	0.00	99.6		58.1			
565GP01-ol-13	35.83	0.00	0.05	0.00	33.74	0.36	28.69	0.34	0.01	0.00	0.00	99.0		60.2			
565GP01-ol-15	35.10	0.02	0.24	0.01	37.83	0.47	25.45	0.44	0.00	0.00	bdl	99.6		54.5			
565GP01-ol-16	36.23	0.00	0.00	0.01	32.31	0.38	30.23	0.24	0.00	0.00	0.03	99.4		62.5			

Spot No.	SiO_2	TiO_2	Al_2O_3	Cr_2O_3	FeO	MnO	MgO	CaO	Na_2O	K_2O	NiO	Total	An	Mg#	En	Wo	Fs
565GP01-ol-17	36.42	0.00	0.02	0.01	33.41	0.37	29.29	0.21	0.00	0.00	0.00	99.7		61.0			
565GP01-ol-18	36.60	0.01	0.03	0.01	30.20	0.34	31.97	0.22	0.00	0.00	bdl	99.4		65.4			
565GP01-ol-19	36.60	0.01	0.04	0.00	30.82	0.35	31.06	0.28	0.00	0.00	0.00	99.2		64.2			
565GP01-ol-21	36.21	0.01	0.06	0.01	31.10	0.36	31.26	0.30	0.00	0.00	0.03	99.3		64.2			
565GP01-ol-22	34.92	0.02	0.06	0.00	39.37	0.40	24.39	0.30	0.06	0.00	0.01	99.5		52.5			
565GP01-ol-23	36.11	0.04	0.05	0.01	33.80	0.33	29.03	0.31	0.00	0.00	0.02	99.7		60.5			
565GP01-px-1	50.86	0.11	0.34	0.09	28.66	0.52	17.93	0.95	0.00	0.00	0.01	99.5		52.7	51.7	2.0	46.4
565GP01-px-2	52.02	0.33	0.66	0.19	22.35	0.41	22.21	1.31	0.00	0.00	bdl	99.5		63.9	62.2	2.6	35.2
565GP01-px-3	50.71	0.34	0.45	0.19	28.23	0.49	17.74	1.42	0.00	0.00	0.03	99.6		52.8	51.2	3.0	45.8
565GP01-px-4	51.50	0.29	0.51	0.16	24.17	0.41	20.86	1.49	0.01	0.00	0.00	99.4		60.6	58.7	3.0	38.2
565GP01-px-5	49.93	0.25	0.40	0.16	29.22	0.57	16.02	2.73	0.02	0.00	0.00	99.3		49.4	46.6	5.7	47.7
565GP01-px-6	50.62	0.57	1.08	0.29	13.83	0.28	11.94	20.24	0.01	0.00	0.03	98.9		60.6	34.8	42.5	22.7
565GP01-px-8	51.15	0.54	0.96	0.33	18.49	0.37	18.03	9.25	0.00	0.00	0.00	99.1		63.5	51.4	19.0	29.6
565GP01-px-9	51.06	0.66	1.39	0.46	11.89	0.25	14.86	18.69	0.10	0.00	0.00	99.4		69.0	42.5	38.4	19.1
565GP01-px-10	50.43	0.23	0.37	0.15	29.89	0.55	16.44	1.89	0.03	0.00	bdl	100.0		49.5	47.5	3.9	48.5
565GP01-px-11	51.78	0.41	0.81	0.23	22.70	0.36	22.09	1.13	0.00	0.00	0.01	99.5		63.4	62.0	2.3	35.8
565GP01-px-13	51.59	0.31	0.54	0.15	23.84	0.41	20.85	1.76	0.01	0.00	0.00	99.5		60.9	58.7	3.6	37.7
565GP01-px-14	51.40	0.24	1.27	0.16	25.16	0.46	19.55	1.65	0.05	0.00	0.00	99.9		58.1	56.1	3.4	40.5
565GP01-px-15	52.24	0.27	0.48	0.15	23.37	0.40	21.31	1.08	0.00	bdl	0.02	99.3		61.9	60.5	2.2	37.3
565GP01-px-16	50.78	0.31	0.51	0.16	28.07	0.46	17.94	1.34	0.00	0.00	0.01	99.6		53.3	51.7	2.8	45.5
565GP01-px-17	50.58	0.62	0.68	0.29	25.33	0.47	19.50	1.75	bdl	0.00	0.02	99.2		57.8	55.7	3.6	40.7
565GP01-px-18	49.69	1.48	2.34	0.70	10.86	0.25	13.61	20.04	0.04	0.00	0.02	99.0		69.1	39.9	42.3	17.9
565GP01-px-19	51.95	0.24	0.55	0.17	23.60	0.42	21.43	1.12	0.00	0.00	bdl	99.5		61.8	60.4	2.3	37.3
565GP01-px-20	51.05	0.25	0.33	0.14	27.56	0.47	18.77	1.13	bdl	0.00	0.00	99.7		54.8	53.5	2.3	44.1
565GP01-px-21	51.87	0.18	0.77	0.24	20.58	0.34	24.91	1.01	0.02	0.00	0.01	99.9		68.3	67.0	2.0	31.1
565GP01-px-22	50.99	0.28	0.38	0.13	27.62	0.43	18.57	1.11	0.00	0.00	0.02	99.5		54.5	53.2	2.3	44.5

Spot No.	SiO_2	TiO_2	Al_2O_3	Cr_2O_3	FeO	MnO	MgO	CaO	Na_2O	K_2O	NiO	Total	An	Mg#	En	Wo	Fs
565GP01-px-23	50.93	0.35	0.49	0.17	26.52	0.45	18.86	1.54	0.01	0.00	0.00	99.3		55.9	54.1	3.2	42.7
565GP01-px-24	50.64	0.31	0.68	0.15	26.62	0.48	18.63	1.59	0.01	0.00	bdl	99.1		55.5	53.7	3.3	43.0
565GP01-px-25	50.66	1.03	1.80	0.47	12.80	0.25	14.57	17.87	0.04	0.00	0.00	99.5		67.0	42.1	37.1	20.8
565GP01-px-26	52.79	0.28	0.58	0.29	19.74	0.34	23.44	2.07	0.05	0.00	0.00	99.6		67.9	65.1	4.1	30.8
565GP01-px-27	52.48	0.30	0.74	0.20	21.37	0.42	22.84	0.94	0.01	0.00	bdl	99.3		65.6	64.3	1.9	33.8
565GP01-px-29	52.00	0.21	0.49	0.13	24.96	0.44	19.94	1.36	0.05	0.00	0.01	99.6		58.7	57.1	2.8	40.1
565GP01-px-30	50.81	0.34	0.31	0.12	28.88	0.47	16.91	2.29	0.03	0.00	bdl	100.2		51.1	48.6	4.7	46.6
565GP01-pl-1-1	42.82	0.02	35.93	0.00	0.18		0.26	19.01	0.36	0.01		98.6	96.6				
565GP01-pl-1-2	42.61	0.03	35.85	0.02	0.30		0.48	19.15	0.31	bdl		98.7	97.1				
565GP01-pl-1-3	42.86	0.02	35.95	0.01	0.31		0.35	19.52	0.29	0.00		99.3	97.4				
565GP01-pl-1-6	43.20	0.00	36.69	bdl	0.33		0.13	19.46	0.29	bdl		100.1	97.4				
565GP01-pl-1-7	42.93	0.00	36.56	0.01	0.15		0.01	19.34	0.38	0.01		99.4	96.5				
565GP01-pl-1-8	43.19	0.01	36.86	0.01	0.13		0.05	19.75	0.21	0.01		100.2	98.0				
565GP01-pl-1-9	42.19	0.05	36.65	0.01	0.21		0.08	19.50	0.26	0.00		98.9	97.7				
565GP01-pl-1-10	42.02	0.00	36.89	0.00	0.20		0.07	19.55	0.33	0.00		99.1	97.0				
565GP01-pl-1-11	43.05	0.02	36.82	bdl	0.15		0.04	19.57	0.29	0.01		99.9	97.3				
565GP01-pl-1-12	43.37	0.01	36.24	0.02	0.18		0.02	19.06	0.42	0.02		99.3	96.1				
565GP01-pl-1-13	43.41	0.02	36.49	0.01	0.30		0.04	19.17	0.26	0.00		99.7	97.6				
565GP01-pl-1-14	43.06	0.02	36.01	0.00	0.48		0.23	19.25	0.25	0.01		99.3	97.7				
565GP01-pl-1-15	42.63	bdl	36.76	0.01	0.14		0.07	19.49	0.23	0.01		99.3	97.9				
565GP01-pl-1-16	43.21	0.01	36.66	0.00	0.27		0.08	19.36	0.34	0.01		100.0	96.8				
565GP01-pl-1-17	42.95	0.02	36.71	bdl	0.21		0.02	19.48	0.37	0.00		99.8	96.7				
565GP01-pl-1-18	43.11	0.01	36.53	bdl	0.25		0.05	19.13	0.26	0.01		99.4	97.5				
565GP01-pl-1-19	43.18	0.02	36.89	0.01	0.13		0.02	19.36	0.33	0.02		100.0	96.9				
565GP01-pl-1-20	43.55	0.02	35.84	bdl	0.16		0.05	19.23	0.44	0.00		99.3	96.0				
565GP01-pl-1-21	42.87	0.01	36.64	bdl	0.20		0.15	19.52	0.27	0.01		99.7	97.5				

Spot No.	SiO_2	TiO_2	Al_2O_3	Cr_2O_3	FeO	MnO	MgO	CaO	Na_2O	K_2O	NiO	Total	An	Mg#	En	Wo	Fs
565GP01-pl-1-22	43.25	0.02	36.43	bdl	0.17		0.08	19.25	0.28	0.00		99.5	97.4				
565GP01-pl-1-23	42.81	0.01	36.97	0.01	0.16		0.07	19.86	0.18	0.01		100.1	98.4				
565GP01-pl-1-24	43.16	0.01	36.33	0.02	0.13		0.04	19.39	0.24	0.00		99.3	97.8				
565GP01-pl-1-25	43.15	0.01	36.88	bdl	0.18		0.03	19.72	0.29	0.01		100.3	97.4				
565GP01-pl-1-26	42.54	0.01	36.48	bdl	0.17		0.03	19.37	0.36	0.01		99.0	96.7				
565GP01-pl-1-28	43.36	bdl	35.89	0.01	0.19		0.03	19.20	0.33	0.01		99.0	96.9				
565GP01-pl-1-29	43.09	0.01	35.93	bdl	0.14		0.05	19.49	0.24	0.00		98.9	97.8				
565GP01-pl-1-30	43.63	0.02	36.76	0.00	0.19		0.04	19.58	0.27	0.00		100.5	97.5				
565GP01-pl-2-1	43.35	0.01	36.50	0.01	0.18		0.10	19.37	0.38	0.01		99.9	96.5				
565GP01-pl-2-2	42.47	0.02	37.12	bdl	0.11		0.04	19.75	0.21	bdl		99.7	98.1				
565GP01-pl-2-3	43.33	0.01	36.63	bdl	0.18		0.05	19.48	0.38	0.00		100.1	96.6				
565GP01-pl-2-4	43.08	0.02	36.59	0.02	0.08		0.01	19.61	0.32	0.00		99.7	97.1				
565GP01-pl-2-5	42.73	0.01	36.60	bdl	0.11		0.01	19.46	0.29	0.00		99.2	97.4				
565GP01-pl-2-6	42.71	0.02	36.32	bdl	0.15		0.05	19.62	0.31	0.00		99.2	97.2				
565GP01-pl-2-7	43.74	0.01	36.46	0.01	0.15		0.09	19.57	0.30	0.02		100.4	97.2				
565GP01-pl-2-8	43.09	0.01	36.44	bdl	0.21		0.03	19.55	0.37	0.01		99.7	96.7				
565GP01-pl-2-9	42.60	0.01	37.02	bdl	0.12		0.01	19.60	0.32	0.00		99.7	97.2				
565GP01-pl-2-10	42.96	0.02	36.69	0.02	0.17		0.09	19.60	0.27	bdl		99.8	97.6				
565GP01-pl-2-11	43.07	0.03	36.27	0.01	0.12		0.02	19.52	0.38	0.01		99.4	96.5				
565GP01-pl-2-12	42.50	bdl	36.80	bdl	0.11		0.04	19.75	0.32	0.01		99.5	97.1				
565GP01-pl-2-13	42.51	0.01	36.63	bdl	0.20		0.06	19.55	0.34	0.01		99.3	96.9				
565GP01-pl-2-14	43.60	bdl	36.35	0.01	0.15		0.02	19.61	0.33	bdl		100.1	97.1				
565GP01-pl-2-15	43.26	0.01	36.55	0.01	0.15		0.06	19.54	0.37	0.01		100.0	96.6				
**EGP11-30**																	
EGP11-30-px-01	50.75	0.22	0.34	0.12	23.64	0.49	13.23	10.56	0.01	bdl	0.01	99.4		49.9	38.8	22.3	38.9
EGP11-30-px-02	52.16	0.39	0.66	0.19	16.17	0.33	12.24	19.05	0.02	0.01	0.00	101.2		57.4	34.9	39.1	25.9
EGP11-30-px-03	52.04	0.51	0.76	0.20	15.41	0.28	11.59	20.15	bdl	bdl	0.03	101.0		57.3	33.4	41.7	24.9

Spot No.	SiO_2	TiO_2	Al_2O_3	Cr_2O_3	FeO	MnO	MgO	CaO	Na_2O	K_2O	NiO	Total	An	Mg#	En	Wo	Fs
EGP11-30-px-04	51.77	0.23	0.44	0.15	23.26	0.46	13.22	11.32	0.00	bdl	0.01	100.9		50.3	38.4	23.7	37.9
EGP11-30-px-05	50.81	0.17	0.28	0.11	28.24	0.52	14.08	6.21	0.02	bdl	bdl	100.4		47.0	40.9	13.0	46.1
**EGP13-08**																	
EGP13-08-px-2	52.28	1.64	1.77	0.44	10.66	0.23	15.60	18.26	0.08	bdl	0.03	101.0		72.3	44.9	37.8	17.2
EGP13-08-px-3	52.50	1.11	1.39	0.41	11.72	0.24	17.73	14.67	0.04	bdl	0.02	99.8		72.9	50.8	30.3	18.9
EGP13-08-px-6	52.98	1.14	1.34	0.41	10.04	0.20	16.65	17.92	0.06	bdl	bdl	100.8		74.7	47.3	36.6	16.0
**EGP13-10**																	
EGP13-10-px-3	54.26	0.28	0.41	0.14	20.98	0.38	22.61	1.55	bdl	bdl	bdl	100.6		65.8	63.7	3.1	33.2
EGP13-10-px-4	53.21	0.26	0.50	0.19	20.60	0.40	22.22	1.35	bdl	bdl	bdl	98.7		65.8	63.9	2.8	33.3
**EGP13-63**																	
EGP13-63-px-1	53.36	0.19	0.27	0.06	26.67	0.46	19.23	1.07	bdl	bdl	0.00	101.3		56.2	55.0	2.2	42.8
EGP13-63-px-2	53.22	0.18	0.40	0.05	26.82	0.49	19.12	1.12	0.01	bdl	bdl	101.4		56.0	54.6	2.3	43.0
EGP13-63-px-3	52.04	0.18	0.33	0.09	26.94	0.43	18.21	1.32	bdl	bdl	bdl	99.5		54.6	53.1	2.8	44.1
EGP13-63-px-4	51.47	0.29	0.40	0.04	32.43	0.53	14.54	1.62	0.05	bdl	0.01	101.4		44.4	42.9	3.4	53.7
EGP13-63-px-5	51.86	0.15	1.31	0.04	29.52	0.45	15.78	1.56	0.07	bdl	0.01	100.7		48.8	47.1	3.4	49.5
EGP13-63-px-6	52.57	0.16	0.49	0.07	25.51	0.47	18.31	2.52	0.01	bdl	0.00	100.1		56.1	53.2	5.3	41.6
EGP13-63-px-7	51.97	0.66	1.27	0.32	11.19	0.21	13.54	20.84	0.06	bdl	0.00	100.0		68.3	38.9	43.1	18.1
EGP13-63-px-8	53.51	0.21	0.48	0.09	25.98	0.43	19.26	1.71	0.03	bdl	0.01	101.7		56.9	54.9	3.5	41.6
EGP13-63-pl-2	44.42	0.02	35.62	bdl	0.40		0.10	19.51	0.41	0.01		100.5	96.3				
EGP13-63-pl-3	44.36	0.01	36.12	bdl	0.27		0.06	19.33	0.34	0.02		100.5	96.8				

ol, olivine; pl, plagioclase; px, pyroxene.

bdl, below the detection limit.

Supplementary Table 4 | Olivine major and trace element compositions measured by EPMA.

Spot No.	SiO_2	MgO	FeO	Ti	Al	Cr	Mn	Ca	Co	Ni	P	Total	Mg#
	wt%			μg g^{-1}									
565GP01-001-ol-16-1	36.69	31.04	32.36	83	515	55	2669	1745	62	36	81	100.8	63.1
565GP01-001-ol-16-2	36.49	30.40	32.90	85	553	53	2667	1773	69	29	66	100.5	62.2
565GP01-001-ol-16-3	36.60	30.83	32.64	94	320	49	2704	1607	75	35	79	100.8	62.7
565GP01-001-ol-16-4	36.55	30.46	32.83	101	178	43	2714	1557	63	27	72	100.5	62.3
565GP01-001-ol-17-1	36.43	30.02	33.61	68	780	44	2646	1961	53	22	88	100.9	61.4
565GP01-001-ol-17-2	36.35	30.23	33.54	82	599	52	2739	1925	67	19	94	100.9	61.6
565GP01-001-ol-17-3	36.25	29.78	33.74	79	1361	46	2809	2288	68	24	103	100.8	61.1
565GP01-001-ol-18-1	37.16	33.14	29.97	75	713	64	2357	1641	69	65	18	101.0	66.3
565GP01-001-ol-18-2	36.79	33.02	30.00	80	423	70	2512	1588	84	72	28	100.5	66.2
565GP01-001-ol-18-3	36.87	33.29	30.25	84	600	79	2550	1688	70	71	14	101.1	66.2

ol, olivine.

Supplementary Table 5 | Average plagioclase trace element compositions (μg g^{-1}) of CE-6 anorthosite clasts.

Clast No.	La	Ce	Pr	Nd	Sm	Eu	Gd	Tb	Dy	Ho	Er	Tm	Yb	Lu
Recrystallised domain	3.26	8.20	1.06	4.66	0.93	0.82	0.94	0.14	0.77	0.14	0.33	0.032	0.38	0.024
007GP01	0.24	0.61	0.070	0.30	0.056	0.73	0.081	0.009	0.064	0.011	0.038	0.005	0.038	0.005
410GP01	0.58	1.43	0.19	0.75	0.17	0.89	0.15	0.027	0.12	0.024	0.060	0.011	0.064	0.007
565GP01	0.37	0.93	0.11	0.50	0.11	0.79	0.094	0.012	0.076	0.011	0.031	0.004	0.030	0.003
EGP11-30	0.61	1.64	0.21	0.76	0.18	0.76	0.116	0.019	0.131	0.025	0.035	0.007	0.040	0.003
EGP13-08	0.59	1.54	0.18	0.71	0.14	0.92	0.151	0.016	0.087	0.018	0.042	0.005	0.045	0.005
EGP13-10	0.21	0.54	0.075	0.24	0.048	0.85	0.024	0.006	0.027	0.003	0.007	0.001	0.005	0.001

Completed plagioclase trace element compositions are provided in Supplementary Table 6. The REE concentrations are reported in μg g^{-1}.

Supplementary Table 6 | Plagioclase trace element compositions (μg g^{-1}) analysed by LA-ICP-MS.

	007G P01-1	007G P01-2	007G P01-3	007G P01-4	410G P01-1	410G P01-2	410G P01-3	410G P01-4	565G P01-1	565G P01-2	565G P01-3	565G P01-4
Sc	0.349	0.232	1.04	1.60	0.321	0.307	1.66	1.28	0.172	0.486	0.282	0.310
V	1.39	1.00	2.41	3.39	1.54	1.47	3.55	2.93	1.63	1.54	0.904	1.32
Cr	0.702	2.49	17.7	52.0	bdl	1.14	47.7	33.0	0.508	10.5	bdl	0.507
Mn	28.2	23.5	52.2	88.1	28.4	26.0	89.1	71.4	27.3	23.9	32.2	32.7
Co	0.164	0.103	0.404	0.830	0.121	0.104	0.450	0.280	0.126	0.274	0.056	0.123
Ni	bdl	0.014	0.132	3.90	bdl	bdl	bdl	bdl	0.311	0.572	0.029	0.098
Cu	0.050	bdl	bdl	0.150	0.161	bdl	0.120	0.330	0.033	0.209	0.077	0.277
Zn	1.64	1.74	1.27	3.40	1.03	1.62	3.00	3.01	bdl	0.629	1.08	0.095
Ga	3.49	3.62	3.61	3.91	3.24	3.55	4.37	4.22	3.11	3.31	3.37	3.07
Rb	0.166	0.422	0.361	bdl	0.115	0.136	1.12	1.15	0.040	0.108	bdl	0.051
Sr	174.4	172.8	170.3	154.2	183.1	190.3	173.4	169.8	165.3	183.6	171.1	175.0
Y	0.244	0.188	0.471	0.370	0.392	0.184	0.850	0.690	0.184	0.419	0.220	0.328
Zr	0.055	0.266	0.738	0.600	0.120	0.129	1.27	0.850	0.048	0.173	0.073	0.013
Ba	9.33	9.14	8.75	7.86	14.2	21.5	50.5	44.0	7.60	14.7	9.33	8.99
La	0.260	0.252	0.243	0.206	0.469	0.278	0.840	0.740	0.186	0.777	0.260	0.265
Ce	0.627	0.700	0.645	0.475	1.17	0.567	2.14	1.82	0.460	1.80	0.719	0.711
Pr	0.072	0.066	0.088	0.053	0.147	0.072	0.308	0.247	0.061	0.213	0.094	0.064
Nd	0.322	0.370	0.347	0.173	0.659	0.287	1.140	0.900	0.208	0.994	0.354	0.396
Sm	0.060	0.061	0.075	0.030	0.131	0.044	0.280	0.237	0.049	0.187	0.088	0.091
Eu	0.765	0.755	0.767	0.620	0.865	0.891	0.900	0.910	0.781	0.839	0.783	0.788
Gd	0.081	0.059	0.080	0.104	0.104	0.057	0.207	0.240	0.040	0.159	0.047	0.084
Tb	0.005	0.004	0.010	0.018	0.019	0.005	0.055	0.028	0.005	0.015	0.008	0.013
Dy	0.044	0.037	0.084	0.091	0.081	0.032	0.210	0.174	0.025	0.118	0.037	0.055
Ho	0.006	0.006	0.016	0.014	0.011	0.007	0.024	0.054	0.006	0.019	0.010	0.007
Er	0.023	0.021	0.045	0.061	0.029	0.008	0.127	0.078	0.036	0.031	0.015	0.020
Tm	0.000	0.003	0.005	0.012	0.004	0.002	0.011	0.028	0.001	0.002	0.002	0.003
Yb	0.019	0.007	0.052	0.074	0.029	0.007	0.084	0.136	0.018	0.015	0.011	0.037
Lu	0.000	bdl	0.005	0.011	0.004	0.001	0.016	0.009	bdl	0.002	0.001	0.000
Hf	0.000	0.000	0.010	0.032	0.000	0.000	0.012	0.040	0.000	0.000	0.000	0.000
Pb	0.034	0.086	0.044	0.047	0.034	0.060	0.035	0.067	0.007	0.041	0.029	0.028
Th	0.003	0.001	0.013	0.006	0.002	0.005	0.006	0.029	bdl	bdl	0.002	bdl
U	0.001	0.003	0.007	0.002	0.001	0.001	0.011	0.016	0.000	0.000	0.003	0.000

Continued

	565G P01-5	565G P01-6	565G P01-7	EGP1 1-30-1	EGP1 1-30-2	EGP1 1-30-3	EGP1 3-08-1	EGP1 3-08-2	EGP1 3-08-3	EGP1 3-08-4	EGP1 3-08-5	EGP1 3-10-1
Sc	0.422	0.550	0.630	0.336	0.370	0.340	0.394	0.930	0.550	0.790	0.730	0.160
V	0.684	1.43	0.820	2.51	0.990	1.20	2.64	1.88	2.34	2.25	2.38	0.853
Cr	0.091	0.350	2.600	1.831	6.300	4.900	1.566	bdl	0.230	bdl	bdl	1.055
Mn	44.2	57.1	42.8	29.3	25.5	26.1	33.4	29.7	33.0	32.1	26.6	23.6
Co	0.169	0.370	0.530	0.221	bdl	bdl	0.048	0.150	0.020	bdl	0.360	0.040
Ni	0.230	bdl	bdl	0.283	bdl	bdl	0.342	bdl	bdl	bdl	bdl	0.037
Cu	0.077	0.860	0.320	0.287	0.820	1.08	0.901	bdl	bdl	bdl	bdl	0.103
Zn	0.968	1.20	1.89	bdl	0.840	bdl	1.74	1.70	1.40	1.20	2.10	0.333
Ga	3.97	3.50	3.75	3.40	4.62	4.15	5.09	5.37	3.06	3.11	4.24	3.94
Rb	0.047	bdl	0.040	0.370	0.540	0.320	0.420	0.390	0.170	bdl	0.040	0.208
Sr	187.9	141.0	156.5	193.3	165.9	167.9	204.6	176.4	157.4	162.6	170.4	185.4
Y	3.949	0.670	3.35	0.588	0.389	0.484	0.611	0.670	0.124	0.198	0.490	0.177
Zr	0.581	0.067	0.770	0.072	0.410	0.400	0.235	0.208	0.034	0.133	0.090	0.237
Ba	23.4	10.2	20.0	13.4	12.4	12.0	32.9	33.0	7.70	8.32	19.9	11.9
La	3.506	0.368	3.02	0.690	0.529	0.606	0.931	1.18	0.115	0.130	0.584	0.208
Ce	9.16	0.970	7.25	1.83	1.49	1.59	2.28	3.23	0.255	0.332	1.61	0.536
Pr	1.08	0.116	1.05	0.220	0.180	0.242	0.280	0.351	0.037	0.053	0.194	0.075
Nd	4.92	0.530	4.40	0.862	0.670	0.750	1.05	1.38	0.112	0.183	0.820	0.239
Sm	0.895	0.148	0.960	0.163	0.230	0.155	0.207	0.300	0.045	0.052	0.098	0.048
Eu	0.926	0.740	0.708	0.823	0.730	0.740	1.06	1.06	0.720	0.800	0.940	0.854
Gd	1.11	0.143	0.770	0.143	0.095	0.111	0.189	0.239	bdl	0.026	0.149	0.024
Tb	0.138	0.017	0.135	0.020	0.015	0.021	0.022	0.030	0.001	0.010	0.017	0.006
Dy	0.810	0.145	0.720	0.157	0.110	0.126	0.119	0.122	bdl	0.027	0.079	0.027
Ho	0.155	0.014	0.124	0.017	0.027	0.030	0.019	0.026	bdl	0.008	0.019	0.003
Er	0.365	0.051	0.285	0.040	0.021	0.044	0.048	0.063	0.051	0.000	0.049	0.007
Tm	0.044	0.012	0.020	0.003	0.005	0.014	0.004	0.011	bdl	0.001	bdl	0.001
Yb	0.315	0.071	0.440	0.043	0.026	0.050	0.043	0.056	0.007	0.024	0.094	0.005
Lu	0.028	0.010	0.020	0.004	0.003	0.002	0.003	0.007	0.005	0.005	0.006	0.001
Hf	0.000	bdl	0.012	0.000	0.045	0.025	0.000	bdl	bdl	0.046	0.030	0.000
Pb	0.178	0.018	0.333	0.066	0.057	0.034	0.101	0.068	0.020	0.031	0.090	0.008
Th	0.417	0.000	0.540	0.001	0.011	0.021	0.011	0.000	0.000	0.000	0.000	0.000
U	0.022	0.000	0.032	0.000	0.016	0.005	0.001	0.000	0.000	0.000	0.000	0.001

bdl, below the detection limit. All data are presented in $\mu g\ g^{-1}$.

Supplementary Table 7 | Plagioclase major and trace element compositions measured by EPMA.

Spot No.	SiO_2	Al_2O_3	CaO	Na_2O	K_2O	Mg	Fe	Ti	P	Total	An
	wt%					μg g^{-1}					
**410GP01**											
410GP01-002-pl trace-01	43.96	36.01	19.75	0.31	0.03	356	1856	133	69	100.4	97.1
410GP01-002-pl trace-02	43.53	35.87	19.89	0.32	0.04	391	1367	107	55	100.0	97.0
410GP01-002-pl trace-03	43.90	36.00	20.06	0.25	0.03	338	1018	99	56	100.5	97.6
410GP01-002-pl trace-04	43.69	35.85	20.59	0.29	0.01	513	1314	107	39	100.8	97.5
410GP01-002-pl trace-05	42.97	35.89	20.65	0.23	0.03	314	1142	116	44	100.0	97.9
410GP01-002-pl trace-06	43.43	35.81	20.50	0.39	0.04	536	2318	73	61	100.6	96.4
410GP01-002-pl trace-07	43.43	35.97	20.57	0.39	0.04	527	2846	90	103	100.9	96.4
410GP01-002-pl trace-08	43.46	35.90	20.64	0.38	0.03	491	2696	70	51	100.9	96.6
410GP01-002-pl trace-09	43.34	36.09	20.52	0.34	0.03	364	1258	150	101	100.6	96.9
410GP01-002-pl trace-10	42.95	36.05	20.57	0.38	0.04	580	2632	177	123	100.5	96.5
410GP01-002-pl trace-11	43.93	35.25	20.18	0.47	0.03	431	1287	139	64	100.2	95.8
410GP01-002-pl trace-12	43.67	35.57	20.37	0.50	0.05	381	1784	135	110	100.5	95.5
410GP01-002-pl trace-13	43.53	36.05	20.44	0.44	0.01	453	1072	128	59	100.8	96.2
410GP01-002-pl trace-14	43.50	36.04	20.73	0.33	0.03	412	1187	99	76	100.9	97.1
410GP01-002-pl trace-15	43.87	35.91	20.42	0.35	0.03	551	1448	95	42	100.9	96.8
410GP01-002-pl trace-16	43.99	35.53	20.39	0.42	0.04	500	1937	157	70	100.8	96.2
410GP01-002-pl trace-17	42.73	36.14	20.55	0.33	0.04	560	2679	132	55	100.3	97.0
410GP01-002-pl trace-18	43.55	35.95	20.27	0.44	0.06	344	1059	98	107	100.5	95.9
**565GP01**											
565GP01-pl trace-1-8	43.44	36.56	20.07	0.24	0.00	248	965	87	80	100.5	97.9
565GP01-pl trace-1-11	43.68	36.54	19.83	0.25	0.01	190	1179	82	69	100.6	97.7
565GP01-pl trace-1-18	44.10	36.34	19.61	0.43	0.00	530	1926	92	106	100.9	96.2
565GP01-pl trace-1-23	43.88	36.29	19.66	0.30	0.01	475	1845	77	99	100.5	97.3
565GP01-pl trace-1-24	43.95	36.43	19.80	0.31	0.00	235	1417	80	54	100.8	97.2

Spot No.	SiO_2	Al_2O_3	CaO	Na_2O	K_2O	Mg	Fe	Ti	P	Total	An
	wt%					μg g^{-1}					
565GP01-pl trace-2-1	43.85	36.39	19.70	0.42	0.01	482	1335	131	45	100.7	96.2
565GP01-pl trace-2-2	43.48	36.50	19.89	0.25	0.00	170	756	81	77	100.3	97.8
565GP01-pl trace-2-3	44.19	36.17	19.85	0.42	0.00	258	1283	79	33	100.9	96.3
565GP01-pl trace-2-4	44.28	36.33	19.92	0.42	0.02	244	776	83	52	101.2	96.3
565GP01-pl trace-2-5	43.93	36.48	19.84	0.36	bdl	230	1024	87	66	100.9	96.8
565GP01-pl trace-2-6	43.90	36.39	20.04	0.28	0.00	278	1055	117	112	100.9	97.5
565GP01-pl trace-2-7	44.05	36.35	19.92	0.36	0.01	297	1258	85	55	101.0	96.8
565GP01-pl trace-2-8	43.79	36.45	19.86	0.37	0.01	286	1569	98	49	100.8	96.7
565GP01-pl trace-2-9	44.17	36.84	19.04	0.28	0.01	200	1501	76	85	100.6	97.4
565GP01-pl trace-2-10	43.48	36.40	19.90	0.34	0.01	515	1757	170	61	100.5	97.0
565GP01-pl trace-2-11	44.39	36.12	19.80	0.36	0.01	275	1220	83	39	100.9	96.8
565GP01-pl trace-2-12	43.86	36.31	19.91	0.29	0.00	204	932	70	58	100.6	97.4
565GP01-pl trace-2-13	43.77	36.40	19.74	0.35	0.01	225	1464	72	45	100.5	96.9
565GP01-pl trace-2-14	43.72	36.73	19.88	0.36	0.01	255	1130	83	52	100.9	96.8
565GP01-pl trace-2-15	44.01	36.42	19.73	0.38	0.01	262	1189	66	51	100.8	96.6
565GP01-pl trace-new-1	43.64	35.72	19.31	0.38	0.01	304	1103	114	298	99.3	96.5
565GP01-pl trace-new-2	44.21	35.97	19.60	0.48	0.01	339	1166	126	201	100.6	95.7
565GP01-pl trace-new-3	44.22	36.20	19.55	0.35	0.01	313	1087	117	139	100.6	96.8
565GP01-pl trace-new-4	43.90	36.35	19.64	0.37	0.01	324	1133	124	132	100.5	96.6
565GP01-pl trace-new-5	43.83	35.94	19.58	0.39	0.01	343	1337	131	144	100.1	96.5
565GP01-pl trace-new-7	44.22	36.33	19.46	0.41	0.00	322	1137	124	155	100.7	96.3
565GP01-pl trace-new-8	44.03	35.96	19.57	0.44	0.01	344	1202	139	123	100.3	96.0
565GP01-pl trace-new-9	44.03	35.80	19.59	0.38	0.01	323	1168	138	148	100.1	96.5
565GP01-pl trace-new-10	43.76	35.94	19.58	0.42	0.01	369	1276	148	166	100.0	96.2
565GP01-pl trace-new-11	43.54	36.83	20.09	0.21	0.01	257	1008	67	96	100.9	98.1
565GP01-pl trace-new-12	44.07	36.40	19.75	0.33	0.01	397	2170	213	72	101.0	97.0

Spot No.	SiO_2	Al_2O_3	CaO	Na_2O	K_2O	Mg	Fe	Ti	P	Total	An
	wt%					$\mu g\ g^{-1}$					
565GP01-pl trace-new-13	43.81	36.58	19.88	0.22	0.00	146	1145	69	95	100.7	98.0
565GP01-pl trace-new-15	43.95	36.54	19.81	0.33	0.01	215	1297	119	96	100.9	97.0
565GP01-pl trace-new-17	44.21	36.26	19.79	0.32	0.01	174	1203	67	79	100.8	97.1
565GP01-pl trace-new-18	43.74	36.51	19.70	0.35	0.01	1331	3399	110	94	101.0	96.9
565GP01-pl trace-new-19	44.01	36.69	19.81	0.34	0.01	235	1403	86	60	101.1	96.9
565GP01-pl trace-new-20	44.16	36.38	19.86	0.37	0.01	259	1475	81	84	101.1	96.6
565GP01-pl trace-new-21	43.99	36.48	19.84	0.27	bdl	263	1010	67	56	100.8	97.6
565GP01-pl trace-new-22	43.93	36.48	19.80	0.28	0.00	222	1152	72	69	100.7	97.5
565GP01-pl trace-new-23	44.01	36.59	19.90	0.30	0.01	207	1024	76	74	101.0	97.3
565GP01-pl trace-new-24	44.29	36.00	19.53	0.40	0.01	710	1798	202	69	100.7	96.4
565GP01-pl trace-new-26	44.08	36.31	19.75	0.43	0.02	361	1705	118	88	100.9	96.1
565GP01-pl trace-new-27	43.21	36.48	20.18	0.23	bdl	224	1158	65	65	100.3	98.0
565GP01-pl trace-new-28	44.03	36.11	19.76	0.41	0.01	382	2127	107	100	100.7	96.4
565GP01-pl trace-new-29	43.42	36.25	19.72	0.34	0.01	361	1707	146	106	100.1	96.9
565GP01-pl trace-new-30	43.73	36.77	19.79	0.36	0.00	341	1589	82	54	101.0	96.8
565GP01-pl trace-new-31	44.28	35.99	19.89	0.24	0.01	522	2588	143	117	100.9	97.8
565GP01-pl trace-new-32	43.76	36.51	20.10	0.21	0.00	223	1124	61	65	100.8	98.1
565GP01-pl trace-new-33	44.05	36.37	19.93	0.28	0.01	264	1334	76	51	100.9	97.4
565GP01-pl trace-new-34	44.29	36.52	19.72	0.32	0.01	268	1272	79	69	101.1	97.1
565GP01-pl trace-new-35	43.51	36.08	19.76	0.25	0.00	532	2773	105	92	100.1	97.7
565GP01-pl trace-new-37	44.42	36.16	19.81	0.29	0.01	359	1876	103	44	101.1	97.4
565GP01-pl trace-new-38	43.47	35.66	19.58	0.35	bdl	308	2017	104	93	99.4	96.8
565GP01-pl trace-new-40	44.52	36.26	19.84	0.45	0.01	289	1435	110	38	101.4	96.0
565GP01-pl trace-new-43	43.91	36.14	19.64	0.38	0.00	519	1792	112	89	100.5	96.6
565GP01-pl trace-new-44	43.51	36.50	19.83	0.20	0.00	811	2608	82	87	100.6	98.2
565GP01-pl trace-new-45	43.34	36.38	20.18	0.14	0.00	351	983	62	44	100.3	98.7

Spot No.	SiO_2	Al_2O_3	CaO	Na_2O	K_2O	Mg	Fe	Ti	P	Total	An
	wt%					μg g^{-1}					
**EGP11-30**											
EGP11-30-pl trace-01	44.25	36.41	19.59	0.41	0.02	147	2099	117	63	101.0	96.3
EGP11-30-pl trace-02	43.58	35.77	19.44	0.38	0.02	110	1275	75	32	99.4	96.5
EGP11-30-pl trace-03	43.94	35.64	19.53	0.40	0.02	257	1674	90	51	99.8	96.4
EGP11-30-pl trace-04	43.38	35.58	19.42	0.40	0.02	811	3514	91	74	99.5	96.3
EGP11-30-pl trace-05	44.30	36.34	19.57	0.43	0.02	565	3503	102	56	101.3	96.0
**EGP13-08**											
EGP13-08-pl trace-01	43.34	35.66	19.52	0.38	0.02	357	922	145	46	99.2	96.5
EGP13-08-pl trace-02	42.72	35.67	19.84	0.25	0.02	270	651	78	62	98.7	97.7
EGP13-08-pl trace-03	42.85	35.66	19.79	0.38	0.01	300	707	148	50	98.9	96.6
EGP13-08-pl trace-04	42.98	36.35	19.90	0.26	0.01	273	606	142	56	99.7	97.6
EGP13-08-pl trace-05	42.67	35.68	19.82	0.24	0.01	275	592	106	43	98.6	97.8
**EGP13-10**											
EGP13-10-pl trace-01	43.10	35.66	19.81	0.38	0.01	110	400	70	53	99.1	96.6
EGP13-10-pl trace-02	43.03	35.59	19.94	0.41	0.01	284	701	75	54	99.2	96.3
EGP13-10-pl trace-03	43.18	35.52	19.76	0.36	0.02	124	375	67	64	99.0	96.7
EGP13-10-pl trace-04	42.87	35.99	19.87	0.28	0.00	134	438	66	59	99.2	97.5
EGP13-10-pl trace-05	43.18	35.72	19.64	0.30	0.01	118	756	72	52	99.0	97.2
**EGP13-63**											
EGP13-63-pl trace-01	43.61	35.61	19.81	0.42	0.00	245	2011	110	60	99.8	96.3
EGP13-63-pl trace-02	43.15	35.55	19.84	0.39	0.01	95	2574	100	94	99.4	96.5
EGP13-63-pl trace-03	42.88	35.28	19.72	0.32	0.01	526	1782	115	50	98.6	97.1
EGP13-63-pl trace-04	43.10	35.62	20.03	0.24	0.01	233	2068	66	66	99.4	97.8
EGP13-63-pl trace-05	42.69	35.43	19.84	0.33	0.01	213	1874	62	70	98.6	97.1

pl, plagioclase.

bdl, below the detection limit.

Supplementary Table 8 | Zircon compositions measured by EPMA.

Spot No.	Zr	Hf	Si	Al	P	Ti
		wt%			μg g^{-1}	
565GP01zrn-1	48.47	1.20	15.30	502	552	75
565GP01zrn-2	47.34	1.17	15.63	295	529	68
565GP01zrn-3	47.94	1.23	15.51	511	520	102
565GP01zrn-4	47.55	1.25	15.54	181	557	64
565GP01zrn-5	48.09	1.21	15.59	467	532	60
565GP01zrn-6	47.98	1.23	15.67	319	525	89
565GP01zrn-7	48.24	1.20	15.90	180	561	61
565GP01zrn-8	47.44	1.24	15.83	485	530	51
565GP01zrn-9	47.94	1.13	15.63	3323	562	76
565GP01zrn-10	47.95	1.27	15.78	215	514	69
565GP01zrn-11	48.35	1.24	15.84	192	533	68
565GP01zrn-12	48.46	1.14	15.17	117	503	92
565GP01zrn-13	49.02	1.12	15.21	562	521	64
565GP01zrn-14	48.10	1.15	15.24	518	524	79

zrn, zircon.

Supplementary Table 9 | Pb-Pb isotope data of zircon within CE-6 anorthosite clast.

Analytical spots	Measured ratios				Radiogenic ratios and ages				^{204}Pb (cps)	%	^{206}Pb (cps)	%
	^{204}Pb/^{206}Pb	%	^{207}Pb/^{206}Pb	%	Radiogenic ^{207}Pb/^{206}Pb	%	^{207}Pb/^{206}Pb Age (Ma)	Err (Ma)				
565GP01-002_Zrn1@1	1.02E-04	22	0.5517	0.37	0.5512	0.37	4,384	11	0.03	22	256	0.22
565GP01-002_Zrn2@2	2.15E-05	35	0.5615	0.27	0.5614	0.27	4,410	8	0.01	35	465	0.16
565GP01-002_Zrn3@1	4.44E-05	22	0.5522	0.25	0.5520	0.25	4,386	7	0.03	22	563	0.15
565GP01-002_Zrn3@2	3.60E-05	22	0.5574	0.22	0.5572	0.22	4,400	6	0.03	22	730	0.13
565GP01-002_Zrn3@3	4.63E-05	22	0.5548	0.25	0.5545	0.25	4,392	7	0.03	22	540	0.15
565GP01-002_Zrn3@4	5.86E-05	19	0.5493	0.24	0.5490	0.24	4,378	7	0.04	19	619	0.14
565GP01-002_Zrn3@5	3.74E-05	21	0.5423	0.21	0.5421	0.21	4,359	6	0.03	21	768	0.13
565GP01-002_Zrn3@6	5.21E-05	21	0.5426	0.25	0.5423	0.25	4,360	7	0.03	21	552	0.15
565GP01-002_Zrn3@7	4.09E-05	22	0.5402	0.24	0.5400	0.24	4,354	7	0.03	22	642	0.14
565GP01-002_Zrn3@8	5.83E-05	22	0.5510	0.29	0.5507	0.29	4,382	8	0.03	22	429	0.17
565GP01-002_Zrn3@9	6.57E-05	21	0.5426	0.29	0.5423	0.29	4,360	9	0.03	21	418	0.17

Zrn, zircon.

Supplementary Table 10 | KREEP assimilation ratio calculated based on plagioclase REE concentrations and high-K KREEP.

Analytical endmembers	La	Ce	Pr	Nd	Sm	Eu	Average
High-K KREEP[1]	110	280	37	178	48	3.30	
Coarse-grained plagioclase in 565GP01	0.29	0.68	0.09	0.36	0.10	0.68	
Measured values of recrystallised plagioclase	3.02	7.25	1.05	4.40	0.96	0.71	
KREEP assimilation ratio (%)	2.49	2.35	2.58	2.27	1.79	1.21	2.12

The REE concentrations are reported in μg g^{-1}.

Supplementary Table 11 | Measured and recommended reference values (μg g^{-1}) for *in situ* trace element analyses.

	ARM-3		GOR128G		GOR132G		NIST612		NIST614	
	Rec.	Measured	Rec.	Measured	Rec.	Measured	Rec.	Measured	Rec.	Measured
P	1178	1178	109	87.3	157	137	48.0	45.2	13.1	8.22
K	26399	26399	299	488	256	287	66.4	95.0	33.2	47.1
Sc	7.00	7.02	32.1	34.0	36.5	34.2	39.9	36.6	0.74	0.91
Ti	6055	6053	1727	1656	1841	1731	42.0	36.6	6.00	1.69
V	12.2	12.2	189	203	214	214	38.8	36.3	1.01	0.96
Cr	9.10	9.07	2272	1913	2528	2518	36.4	29.3	1.19	0.31
Mn	387	387	1363	1314	1193	1057	39.0	35.5	/	1.32
Co	7.50	7.45	92.0	89.0	92.7	98.3	35.5	31.9	0.79	0.64
Ni	12.3	12.3	1074	1046	1187	1265	38.8	35.1	1.10	0.84
Cu	13.6	13.6	63.8	60.9	205	215	37.8	37.0	1.37	4.06
Zn	36.1	36.1	74.7	63.8	76.8	73.3	39.1	43.2	2.79	2.81
Ga	17.7	17.7	8.70	8.72	10.4	11.2	36.9	34.2	1.31	1.07
Rb	7.50	7.54	0.41	0.37	2.10	2.02	31.4	29.3	0.86	0.77
Sr	19.6	19.6	30.0	30.8	15.30	14.2	78.4	69.5	45.8	38.4
Y	7.05	7.05	11.8	12.1	12.90	12.4	38.3	32.2	0.79	0.63
Zr	10.9	10.9	10.0	10.2	9.90	9.65	37.9	32.8	0.85	0.64
Nb	12.2	12.2	0.10	0.09	0.07	0.05	38.9	34.9	0.82	0.69
Cs	7.53	7.53	0.24	0.21	7.45	7.26	42.7	39.4	0.66	0.61
Ba	28.4	28.4	1.06	1.07	0.82	0.74	39.3	34.1	3.20	2.77
La	6.62	6.62	0.12	0.11	0.08	0.09	36.0	31.4	0.72	0.58
Ce	7.97	7.97	0.45	0.39	0.39	0.30	38.4	32.9	0.81	0.64
Pr	5.68	5.68	0.10	0.10	0.09	0.09	37.9	32.5	0.77	0.62
Nd	7.53	7.53	0.78	0.79	0.69	0.69	35.5	30.3	0.75	0.57
Sm	6.00	6.00	0.53	0.53	0.51	0.54	37.7	32.1	0.75	0.63
Eu	5.71	5.71	0.26	0.27	0.26	0.25	35.6	30.6	0.77	0.64
Gd	6.00	6.00	1.17	1.21	1.19	1.20	37.3	32.0	0.76	0.62
Tb	6.62	6.62	0.25	0.24	0.27	0.25	37.6	31.5	0.74	0.59
Dy	6.18	6.18	1.98	1.98	2.15	2.08	35.5	30.3	0.75	0.58
Ho	7.17	7.17	0.44	0.47	0.51	0.49	38.3	32.7	0.75	0.60
Er	6.28	6.28	1.40	1.40	1.56	1.58	38.0	32.3	0.74	0.59
Tm	6.47	6.47	0.20	0.20	0.23	0.23	36.8	31.6	0.73	0.57
Yb	8.88	8.88	1.41	1.33	1.61	1.56	39.2	31.4	0.78	0.61
Lu	6.48	6.48	0.21	0.21	0.24	0.22	37.0	30.7	0.73	0.56
Hf	6.21	6.21	0.35	0.30	0.36	0.36	36.7	32.0	0.71	0.59
Ta	6.16	6.16	0.02	0.02	0.03	0.03	37.6	31.5	0.81	0.60
Pb	12.7	12.7	0.35	0.25	19.5	18.9	38.6	35.0	2.32	1.99
Th	3.28	3.28	0.01	0.01	0.01	0.00	37.8	31.0	0.75	0.57
U	3.75	3.75	0.01	0.01	0.05	0.04	37.4	32.0	0.82	0.67

3. Supplementary Reference

1. Warren, P. H. *KREEP: Major-Element Diversity, Trace-Element Uniformity (Almost)*. 149–153 (1989).
