# Supplementary material for: Chang’e-6 farside anorthosites indicate hemispherically comparable magma ocean solidification
Source: Nat Commun. 2026 May 15;17:6467. doi: 10.1038/s41467-026-73258-y (PMC13376548; doi:10.1038/s41467-026-73258-y)
Supplement: Supplementary file 2 — Transparent Peer Review file [file 41467_2026_73258_MOESM2_ESM.pdf]

nature portfolio

Peer Review File

Chang’e-6 farside anorthosites indicate hemispherically comparable magma ocean solidification

Corresponding Author: Professor Yi Chen

This file contains all reviewer reports in order by version, followed by all author rebuttals in order by version.

Version 0:

Reviewer comments:

Reviewer #1

(Remarks to the Author)
Dear Professor Chen and Co-Authors,

I was delighted to be asked to review the manuscript you submitted to Nature Communications with the title ""Chang’e-6 farside anorthosites reveal globally homogeneous lunar magma ocean solidification".

It represents an extraordinary multi-analytical assessment of the small fragments obtained by the incredible Chang'e-6 mission to the lunar farside. Although, the data presented is compelling, there are a few issues that are of concerned and not mentioned.

1- The crustal thickness dichotomy of the Moon, i.e., the crustal thickness difference between the near and farside, and how this could influence the chemical composition of the crustal material when comparing material from near and farside, and especially the difference in surficial expression of KREEP. Please check the work by Khan et al, such as this 2024 review and references therein. "Composition, Structure and Origin of the Moon": https://arxiv.org/abs/2408.16840

2- Considering that any of the lunar samples collected by missions was obtained from the upper most layer of the lunar surface, they represent material ejected by a wide range of impacts. Especially, events that formed the large basins (the last such event formed Orientale basin) ejected material that travelled around the lunar globe. Please see the paper by Werner et al (2022) "Orientale ejecta at the Apollo 14 landing site implies a 200-million-year stratigraphic time shift on the Moon" https://iopscience.iop.org/article/10.3847/PSJ/ac54a6/meta where estimation of ejecta thickness versus distance from crater are presented. Thus, there is a need to discuss or at least consider that the Chang'e-6 anorthositic clast with the zircon, and that is carefully presented in your manuscript, maybe material ejected from a crater/basin formed on the nearside and not therefore a sample of the farside crust. The is especially important because of many similarities, versus the observable, measured and modelled chemical and structural dichotomies when comparing lunar nearside and farside.

Minor comments:
1- In line 58, where the percentage of each material type the 2000 fragments represent, how similar is the distribution of the different clasts to the Apollo and Luna regolith?

2- In line 154, where reference 49 is referred to, please add reference 40.

Wishing All the best.
Kind Regards,
Vera Assis Fernandes

Reviewer #2

(Remarks to the Author)
This manuscript presents data from seven millimetric lunar far-side soil clasts interpreted as derived from ferroan anorthosites (FAN). The main conclusion of the manuscript, that the far-side crust is comparable to the near-side crust is somewhat circular and drawing global tectonic conclusions from a few soil grains is unjustified.

In detail:
- L.61 ff: How are these clasts, which are 0.5% of the clast-population, identified as “anorthositic” ? As far as I understand, they are picked because they are similar to the near-side anorthosites. This invalidates any conclusion that both sides are similar, completely circular argument.
- L81f, 111, 146: The zircons are described as eu- to subhedral while they are largely xenomorphic (Fig.1c). There is not a single idiomorphic zircon crystal face in this figure, this claim is very misleading. Thus, the argument that the zircons necessarily date the plagioclase crystallization in this grain is erroneous. Exactly contr4ary to what is stated in l.146, the texture does show that the zircons are in textural disequilibrium with the silicate matrix.
- L. 121, 212, Fig. 3: The authors confound “date” and “age”. There is one measurement spot that has 4410 Ma, but the full series ranges to 4360 Ma. The other dates are interpreted away, but this is unreasonable.
- L144: The “recrystallization temperature” is based on rutile-saturation, which does not apply here. Also, the clast with the zircons has no ilmenite documented, hence a(Ti) is undetermined and this thermometer cannot be applied.
Note that this temperature is identical or superior to the crystallization temperature of anorthosites (see review of experimental constrains by Schmidt+Krattli), why would it be a recrystallization T ?
- L. 163: Why is KREEP post LMO ? KREEP is the ultimate residual melt of the LMO, so it is definitely part of the LMO crystallization.
- L. 166: There is confusion here. Now the argument is KREEP-component bearing high Mg-magmatism.
- L. 169ff: These arguments are not acceptable and logically flawed. No matter what the details are, the petrological process of magma ocean crystallization will result in similar rock types formed upon final crystallization. The nature of crystallization process is that they converge and give the same rocks even if primary compositions and volumes might differ. This does not allow to conclude anything on the global scale tectonics, but only confirms petrological principles when approaching a eutectic. Yes, there was a magma ocean on the far-side, but how this magma ocean crystallized, how thick the resulting crust and which detailed composition cannot be extracted from a few millimetric clasts.
- L. 208: why do you expect a precise age for the final crystallization of the lunar magma ocean ? This might be well different in different places, models sowing that the final crystallization is protracted over 200 Ma, meaning that anorthositic cumulates might be forming over 200 Ma. Pernet-Fisher et al showed that anorthosites involve a lot of reworking, consistent with a crust on a giant magma ocean that froms over >100 Ma, this is completely ignored.

In summary, the conclusions are largely based on the choice of initial assumptions and translate into a completely static view of what happened during magma ocean crystallization. On principal, there can’t be a precise age of anorthosite formation, and whatever the details are, the late crystallization products are governed by petrological principles and not by global dynamics or tectonics. The manuscript does hence not plausible constrain processes that led to the dichotomy in the lunar crust.

Version 1:

Reviewer comments:

Reviewer #1

(Remarks to the Author)
Dear Prof Chen and Co-Authors,

Thank you for your revision and consideration to my comments.

An extra comment, overall, we will ever know where the small lunar regolith samples come from and their tumbling around history on the lunar surface since 4.4+ Ga. Additionally, of course the Orientale Basin forming event could have targeted material as old as the zircon dated in the manuscript, since it is younger than the rock the zircon is within. Additionally, the target area of the event that formed Orientale was already covered by older ejecta from the many basin forming events prior to this last basin. These events excavated many old rocks. The stack of ejecta (i.e., the many ejecta stratigraphy) that would be also ejected upon the Orientale Basin event could have all sort of ages prior to the ~3.9 Ga event that formed Orientale Basin. The shock pressure is not all the same across the impacted area, or its effect on the targetted materials, and there is post-shock temperature increase gradient across the newly formed basin.

This is a deep set of caveats that shouldn't be ignored or brushed over, and therefore further consideration taken in the manuscript.

I hope there will be the day astronauts can do field work (ONLY) on the Moon, and really find the proper connection of rocks and their sources.

Kind Regards,
Vera Assis Fernandes

Reviewer #2

(Remarks to the Author)
Overall, the authors have done a great job to improve the manuscript and to much clearer distinguish between observations and interpretations. Some over-interpretations have been removed, but not all.
The ages of the anorthositic nearside crust are widespread. You have one sample, please do not conclude that the entire farside anorthositic crust has been solidified based on one sample, solidification begins with more or less crystallized rafts in a magma ocean and continues with occasional reworking (e.g. Pernet-Fisher). Your data can only show that partial solidification has occurred by 4.41 Ga, but not that the anorthositic crust was completely formed at 4.41 Ga; see Fig. 3). Physically, the end of crystallization will be very protracted as the final solidification within a crystal mush is extremely protracted and will be locally quite different.

Regarding the lunar asymmetry, I realize that reviewer 1 and me have a somewhat disparate opinion here and that the authors may naturally struggle a bit to satisfy both.

Nevertheless, the asymmetry cannot form during a magma ocean stage with a true liquid layer (i.e. not as long as there is a magma layer that has crystals suspended). This is nicely pointed out by Nimmo et al. 2024, Nature (cited as reference #84), who clearly state that the freezing-in of the Moon's bulge requires a rigid elastic layer. The main topic of this paper is that there should be a tidally driven re-melting event at about 4.35 Ga due to the Moon's transition of the Laplace plane (at which point the Moon is not yet tidally locked), driving e.g. the formation of the (KREEP bearing) high-Mg suite. The Nimmo paper remains unclear whether this tidal remelting also effects the anorthositic crust (the carton of their figure 1 would indicate yes), yet points out the resetting of the zircon ages corresponding to this tidal remelting.
The implications I draw from the Nimmo paper (and references contained therein) for this manuscript are: the asymmetry is not directly related to the magma ocean (great support for the hypothesis of the authors), but at the same time, the asymmetry is said to have been there at 4.35 Ga. This is not a contradiction to a 150-200 Ma span for LMO solidification (Maurice et al.), as the waning stage is a (possibly not anymore global) crystal mush that may be quite rigid. Further, the zircon resetting may not need a heat source external to the observed rock fragment(i.e. the Mg-suite), if tidal heating alone can do it. This can't be resolved at this point, but needs to be mentioned/discussed.

In this sense, I do not see the pertinence of the cartoon of Fig.4, in any case, I have serious qualms with this "contact metamorphism" through high-Mg suite magmas. This contact metamorphism will affect an infinitesimal small volume of the anorthositic crust and the likelihood that your sample comes exactly from such a contact aureole ought to be similarly small. A rather widespread tidal remelting seems to be much more plausible.

Regarding the discussion of zircon morphology: Ext.Data Figures 5 and 6 fail to show any subhedral zircon, its simply not there. Yet, I do not understand why this is such an issue, i.e. why insist on eu- or subhedral shapes. If there was a remelting/recrystallization event that went beyond zircon saturation, the zircons could have any shape.

As I checked some references in the manuscript, I have seen that there are 87 of them, while standard Nature policy is ~50. Although this is an editorial concern, I wonder how the main text arguments will look like once 1/3 of the references are cut.

Finally, the last (added statement) of the bold paragraph is only very indirectly true. The arguments of this manuscript place the reasons for the lunar dichotomy elsewhere, how does this "help to better understand lunar dichotomy" ?

Version 2:

Reviewer comments:

Reviewer #2

(Remarks to the Author)
There is still some uneasiness from both reviewers about considering adequate (yet inherent) uncertainties. On the Moon, any interpretation need to be put into context, i.e. other possibilities in genesis need to be acknowledged. This is my third reading of the manuscript and I believe that we arrived more or less where one can arrive. I would ask the authors, to make another effort to claim a little bit less certainty, where certainty does not exist (the arguments have all been made in the previous reviews), as there is really nothing to be gained but much credibility to be lost by affirming too much. There are large uncertainties in any of the lunar geology/selenology and I believe the text could still be improved with respect to these, i.e. some wording is still too affirmative.

I have only two minor comments: (1) The revisions now state that "The recrystallisation age of 4.41 Ga serves as a pivotal snapshot of the termination of farside LMO solidification, anchoring the timeline of early lunar evolution.". THhs is simply incorrect, the 4.41 dates the latest beginning or in other words that some of lunar farside anorthosite has formed, but certainly not all of the lunar farside. You simply don't know when termination/full crystallization of the lunar magma ocean occurred.
(2) The zircons are xenomorph and their grain shape cannot be taken as indicating any equilibrium or paragenetic relation, from their shape, they could be e.g. be impact detrital. Unless the authors provide other BSE images, this has be taken into account.

Open Access This Peer Review File is licensed under a Creative Commons Attribution 4.0 International License, which permits use, sharing, adaptation, distribution and reproduction in any medium or format, as long as you give appropriate credit to the original author(s) and the source, provide a link to the Creative Commons license, and indicate if changes were made.
In cases where reviewers are anonymous, credit should be given to 'Anonymous Referee' and the source.
The images or other third party material in this Peer Review File are included in the article's Creative Commons license, unless indicated otherwise in a credit line to the material. If material is not included in the article's Creative Commons license and your intended use is not permitted by statutory regulation or exceeds the permitted use, you will need to obtain permission directly from the copyright holder.
To view a copy of this license, visit https://creativecommons.org/licenses/by/4.0/

REVIEWER COMMENTS

Reviewer #1 (Remarks to the Author):

Dear Professor Chen and Co-Authors,

I was delighted to be asked to review the manuscript you submitted to Nature Communications with the title ""Chang'e-6 farside anorthosites reveal globally homogeneous lunar magma ocean solidification".

It represents an extraordinary multi-analytical assessment of the small fragments obtained by the incredible Chang'e-6 mission to the lunar farside. Although, the data presented is compelling, there are a few issues that are of concerned and not mentioned.

We are grateful for the reviewer's positive evaluation of our scientific research. We take into full account these suggestions/comments that greatly improve the presentation of our manuscript.

The Line numbers mentioned below refer to those in the revised manuscript with track changes.

Responses are in blue. **Revisions** are in red.

1- The crustal thickness dichotomy of the Moon, i.e., the crustal thickness difference between the near and farside, and how this could influence the chemical composition of the crustal material when comparing material from near and farside, and especially the difference in surficial expression of KREEP. Please check the work by Khan et al, such as this 2024 review and references therein. "Composition, Structure and Origin of the Moon": https://arxiv.org/abs/2408.16840

Reply: We sincerely appreciate the reviewer's insightful comment regarding the potential influence of the lunar crustal thickness dichotomy on the chemical composition of crustal materials, particularly in relation to the surficial distribution of KREEP. Thank you for directing our attention to the comprehensive review recommended and other relevant references. We

have carefully examined these works and reconsidered the possible genetic connections between the nearside–farside crustal thickness asymmetry and crustal composition.

GRAIL gravity observations indicate that the nearside crust is approximately 13-17 km thinner than the farside crust. This asymmetry appears to be linked to the distribution of the KREEP-rich component and mare basalts. The thicker farside crust, if acting as the primary LMO crust, reflects earlier plagioclase flotation and accumulation on the farside (Gaffney et al., 2023; Sossi et al., 2024) and may have facilitated the concentration of incompatible elements on the nearside (Wasson & Warren, 1980; Andrews-Hanna et al., 2025). This could explain the remarkable concentration of incompatible elements within the Procellarum KREEP Terrane (PKT) on the nearside and the relative scarcity of surface KREEP signatures on the farside (e.g., Sossi et al., 2024). A key unresolved question is whether the thicker farside crust represents the primary LMO crust.

If the asymmetry were primarily due to LMO crystallization, one might expect earlier plagioclase flotation and accumulation on the farside, potentially resulting in a more magnesian crustal composition there. However, our geochemical data show that the farside anorthosites sampled by Chang'e-6 exhibit major and trace element compositions similar to those of nearside FANs. This finding suggests that the observed crustal dichotomy is more likely the result of post-LMO events rather than primordial asymmetric solidification.

We have incorporated a discussion of this point, based on the recommended references, into the revised manuscript.

Revision: **Lines 37-39**: However, orbital observations have revealed a striking hemispheric dichotomy in the composition and thickness of the ancient lunar crust, with the farside anorthositic highlands being more magnesian and thicker than its nearside counterpart[5–8].

Lines 235-240: More rapid cooling and crystallisation of farside LMO would promote earlier flotation and accumulation of plagioclase, produce a thicker and more magnesian primary farside crust[80,81], and leave KREEP-rich residual liquid concentrated under the nearside crust[10,82]. However, the compositional similarity of the CE-6 anorthosites with nearside FANs and the presence of KREEP signatures in farside samples contradict this scenario.

2- Considering that any of the lunar samples collected by missions was obtained from the upper most layer of the lunar surface, they represent material ejected by a wide range of impacts. Especially, events that formed the large basins (the last such event formed Orientale basin) ejected material that travelled around the lunar globe. Please see the paper by Werner et al (2022) "Orientale ejecta at the Apollo 14 landing site implies a 200-million-year stratigraphic time shift on the Moon" https://iopscience.iop.org/article/10.3847/PSJ/ac54a6/meta where estimation of ejecta thickness versus distance from crater are presented. Thus, there is a need to discuss or at least consider that the Chang'e-6 anorthositic clast with the zircon, and that is carefully presented in your manuscript, maybe material ejected from a crater/basin formed on the nearside and not therefore a sample of the farside crust. This is especially important because of many similarities, versus the observable, measured and modelled chemical and structural dichotomies when comparing lunar nearside and farside.

Reply: We sincerely thank the reviewer for bringing this crucial point to our attention. We fully agree that the potential transport of material across the lunar surface, particularly from large nearside basins such as Orientale. The Orientale basin (~930 km in diameter) is one of the most potential craters to eject materials to considerable distances (Meyer et al., 2020; Gao et al., 2025). We have therefore carefully considered this possibility. However, several lines of evidence derived from our sample analysis and regional geological context suggest a local (farside) origin is more probable.

First, electron backscatter diffraction (EBSD) analysis of the plagioclase grains reveals low cumulative misorientation angles (<3°), indicative of low-strain deformation. This feature is inconsistent with the severe shock and plastic deformation typically associated with high-velocity ejecta transported over long distances. Instead, the microstructure was likely excavated and redeposited by a relatively local impact event.

Second, the U–Pb zircon age of 4410 ± 8 Ma obtained from the anorthosite clast predates the generally accepted formation age of the Orientale basin (~3.81–3.92 Ga; Whitten et al., 2011; Werner et al., 2022). Long-range ejecta would be expected to reflect the age of the basin-

forming event, either as a primary crystallization age or as a resetting age (Joy et al., 2024). The antiquity of the zircon in our sample makes it unlikely to have been delivered by Orientale.

Third, the Chang'e-6 landing site is located approximately 1700km from the Orientale basin. Models of ejecta distribution suggest that ejecta thickness decreases rapidly with distance (Melosh, 1989). At such a range, ejecta from Orientale would be expected to be thin and likely buried by subsequent ejecta from local craters (e.g., Chaffee S and White) and basaltic volcanism (e.g., ~2.8 Ga mare basalts in the landing region). Indeed, recent modeling work suggests that ejecta from more recent, smaller craters (e.g., Vavilov, with ejecta ~1.7-1.8 cm thick) dominate the anorthositic materials in the Chang'e-6 regolith (Yue et al., 2024; Gou et al., 2024).

Lastly, remote sensing data reveal the presence of pre-Nectarian anorthositic terrains in the northeastern Apollo basin and adjacent regions (Guo et al., 2024, 2025). These ancient crustal units are a more probable source of the anorthositic clasts recovered by Chang'e-6 than long-distance ejecta.

In summary, while we acknowledge the possibility of exotic nearside ejecta, the combined microstructural, geochronological, and regional geologic evidence supports a local, farside origin for the anorthositic clasts. We have expanded the discussion in the revised manuscript to include this important consideration and cite the recommended literature.

Revision (**Lines 176-187**): The CE-6 anorthosites represent exotic materials ejected from a wide range of craters, even including nearside large impact basins such as Orientale, whose formation was capable of distributing material across the global lunar surface[54,55]. However, several lines of evidence favour a local, farside origin for the CE-6 anorthositic clasts. Nearside large impacts would be expected to induce severe shock and plastic deformation of ballistic ejecta[40,56,57] and reset their crystallisation age[58], which cannot explain the low-strain microstructure observed in the zircon-bearing anorthosite and the 4.41 Ga age predating the Orientale (~3.81–3.92 Ga; ref.[59,60]) and other nearside impacts[61]. Ejecta modeling indicates that at the CE-6 landing site, material from distant ancient basins would be very thin and likely buried by local basalts and ejecta from Copernican craters[62,63]. Remote sensing data reveals abundant pre-Nectarian anorthositic regions within the Apollo basin and adjacent highland

craters such as Vavilov[64–66], providing a more probable local source than nearside ejecta.

Minor comments:

1- In line 58, where the percentage of each material type the 2000 fragments represent, how similar is the distribution of the different clasts to the Apollo and Luna regolith?

Reply: Thank you for this valuable comment. We have revised the manuscript to include a systematic comparison with Apollo and Luna samples.

The Chang'e-6 soil (comprising ~35% mare basalt, ~45% breccia, ~15% glass, and non-mare lithic clasts) differs notably from that of nearside mare sites (e.g., Apollo 11, 12; Luna 16, 24), where basaltic fragments typically range from 7–24% and breccias and glasses are significantly less abundant (Simon et al., 1981). Highland sites (Apollo 14, 16; Luna 20) are dominated by highland lithologies and breccias, while boundary sites (Apollo 15, 17) contain mixed components with generally <10% mare basalts. The Chang'e-6 samples, in contrast, shows a distinct enrichment in impact-derived materials (breccias and glasses) relative to all these nearside regions. This compositional signature is consistent with the intense and prolonged impact history within the South Pole–Aitken basin, suggesting that the farside regolith at the landing site has undergone more extensive impact reworking than typical nearside localities.

We have incorporated this comparative analysis into the revised manuscript to better contextualize the provenance and formation history of the CE-6 samples.

Revision (**Lines 57-59**): The fragments consist of mare basalt (35%), breccia (45%), glass (15%), and non-mare lithic clasts (5%), with an enrichment in basaltic fragments and glasses relative to those in the regolith from nearside mare sites[26].

2- In line 154, where reference 49 is referred to, please add reference 40.

Revision (**Line 161**): The relevant reference has been added to the revised manuscript.

Wishing All the best.

Kind Regards,

Vera Assis Fernandes

REFERENCES in REPLY:

Andrews-Hanna, J. C. *et al.* Southward impact excavated magma ocean at the lunar South Pole–Aitken basin. *Nature* **646**, 297–302 (2025).

Gaffney, A. M. et al. Magmatic Evolution I: Initial Differentiation of the Moon. Rev. *Mineral. Geochem.* 89, 103–145 (2023).

Gao, A. *et al.* Geological Background of the Chang'e 6 Landing Site and the Provenance of Returned Samples. *J. Geophys. Res. Planets* **130**, e2024JE008658 (2025).

Gou, S. *et al.* Complex basalt evolution in the Chang'e-6 landing area. *Earth Planet. Sci. Lett.* **648**, 119091 (2024).

Guo, D. *et al.* A lunar time scale from the perspective of the Moon's dynamic evolution. *Sci. China Earth Sci.* **67**, 234–251 (2024).

Guo, D. et al. Crystalline Ferroan Anorthosite Identified in the Lunar Apollo Basin. *J. Geophys. Res.* Planets 130, (2025).

Joy, K. H. et al. Evidence of a 4.33 billion year age for the Moon's South Pole–Aitken basin. *Nat. Astron.* https://doi.org/10.1038/s41550-024-02380-y (2024) doi:10.1038/s41550-024-02380-y.

Melosh, H. J. Impact Cratering: A Geologic Process. Oxford University Press. (1989).

Meyer, H. M., Denevi, B. W., Robinson, M. S. & Boyd, A. K. The Global Distribution of Lunar Light Plains From the Lunar Reconnaissance Orbiter Camera. *J. Geophys. Res. Planets* **125**, e2019JE006073 (2020).

Simon, S. B. & Papike, J. J. The Lunar Regolith: Comparative Petrology of the Apollo and Luna Soils. in 984–986 (1981).

Sossi, P. A., Nakajima, M. & Khan, A. Composition, Structure and Origin of the Moon. Preprint at https://doi.org/10.48550/arXiv.2408.16840 (2024).

Wasson, J. T. & Warren, P. H. Contribution of the mantle to the lunar asymmetry. *Icarus* **44**, 752–771 (1980).

Werner, S. C., Bultel, B., Rolf, T. & Assis Fernandes, V. Orientale Ejecta at the Apollo 14 Landing Site Implies a 200-million-year Stratigraphic Time Shift on the Moon. *Planet. Sci. J.* **3**, 65 (2022).

Whitten, J. *et al.* Lunar mare deposits associated with the Orientale impact basin: New insights into mineralogy, history, mode of emplacement, and relation to Orientale Basin evolution from Moon Mineralogy Mapper (M^3) data from Chandrayaan-1. *J. Geophys. Res.* **116**, E00G09 (2011).

Yue, Z. et al. Geological context of the Chang'e-6 landing area and implications for sample analysis. *The Innovation* 5, 100663 (2024).

Reviewer #2 (Remarks to the Author):

This manuscript presents data from seven millimetric lunar far-side soil clasts interpreted as derived from ferroan anorthosites (FAN). The main conclusion of the manuscript, that the far-side crust is comparable to the near-side crust is somewhat circular and drawing global tectonic conclusions from a few soil grains is unjustified.

We appreciate the reviewer's constructive and critical comments. These comments and suggestions are all valuable for revising our paper, as well as for providing important guidance for our future work. We have addressed all comments and made the necessary changes in the revision.

In detail:

- L.61 ff: How are these clasts, which are 0.5% of the clast-population, identified as "anorthositic" ? As far as I understand, they are picked because they are similar to the near-side anorthosites. This invalidates any conclusion that both sides are similar, completely circular argument.

Reply: We thank the reviewer for raising this important point regarding the petrological classification of the clasts and the potential for circular reasoning. We appreciate the opportunity to clarify our methodology and the basis for identifying these particles as anorthositic. The identification was based strictly on the established petrological definition of anorthosite, which requires a modal plagioclase abundance exceeding 90% (Buddington, 1939; Streckeisen, 1976). This standard defines both the anorthosites on Earth and the Moon. The clasts selected for this study contain 90.8–97.5 vol% plagioclase, unambiguously meeting this criterion. This initial selection was made solely on the basis of mineral abundance, prior to any comparative geochemical analysis with nearside samples.

We have clarified this definition in the revised manuscript to avoid ambiguity and any perception of circular logic.

Revision (**Lines 66-71**): The studied clasts are 0.3–2 mm in size and dominated by plagioclase (90.8–97.5 vol%) and low-calcium (Ca) pyroxene (2.1–8.6 vol%), with minor high-Ca pyroxene, olivine, chromium (Cr)-spinel, ilmenite, troilite, silica phases (quartz and tridymite), and zircon (Fig. 1, Extended Data Fig. 1, Supplementary Table 1). These clasts were identified as anorthosite according to their mineral abundances and petrological definition (ref.[27]; Extended Data Fig. 2).

- L81f, 111, 146: The zircons are described as eu- to subhedral while they are largely xenomorphic (Fig.1c). There is not a single idiomorphic zircon crystal face in this figure, this claim is very misleading. Thus, the argument that the zircons necessarily date the plagioclase crystallization in this grain is erroneous. Exactly contrary to what is stated in l.146, the texture does show that the zircons are in textural disequilibrium with the silicate matrix.

Reply: We thank the reviewer for this careful observation and for providing an alternative interpretation for the zircon morphology and their textural relationship with the matrix plagioclase. We acknowledge that the term "euhedral" may be too strong for the zircons in Fig.1c, and we have revised the description in the manuscript to "subhedral" to more accurately reflect their morphology.

Apollo sample studies indicate that idiomorphic zircons typically exhibit hexagonal outlines with straight crystal boundaries, while the xenomorphic grains are rounded and irregular granular-textured (Nemchin et al., 2008; Grange et al., 2011; Zhang et al., 2021). Our cathodoluminescence (CL) and electron backscatter diffraction (EBSD) analyses reveal straight grain boundaries and ~120° triple junctions between the zircon and surrounding plagioclase (Fig. 1d, Extended Data Figs. 5, 6). Such interfacial angles are a well-established criterion for textural equilibrium in high-temperature metamorphic systems, indicating coeval and static recrystallization rather than mechanical incorporation of xenocrysts.

We acknowledge that the polishing plane may not intersect all zircons in an orientation that perfectly preserves their original shape. Nevertheless, the combined microstructural evidence supports our interpretation that the zircons grew *in situ* during thermal metamorphism. To avoid any misunderstanding, we have added a more detailed description of the zircon morphology and the criteria for assessing texture equilibrium in the revised manuscript.

Revision **Lines 79-86**: A distinct 0.3 × 0.4 mm^2 recrystallisation domain within this clast shows a granoblastic texture, as evident in Cathodoluminescence (CL) and electron backscatter diffraction (EBSD) images (Fig. 1d, Extended Data Figs. 5, 6). This domain consists of fine-grained plagioclase (10–30 μm) with straight grain boundaries and equilibrated ~120° triple junctions. Four zircon grains within this domain exhibit similar equilibrium boundaries with plagioclase (Fig. 1d, Extended Data Figs. 5, 6), indicative of coeval, static recrystallisation of these two phases rather than mechanical incorporation of xenocrysts.

Lines 115-117: All of zircons occur as subhedral grains in contact with the fine-grained plagioclase and exhibit homogeneous CL structures (Extended Data Fig. 5) and uniform compositions (Supplementary Table 6), supporting a co-genetic origin.

- L. 121, 212, Fig. 3: The authors confound “date” and “age”. There is one measurement spot that has 4410 Ma, but the full series ranges to 4360 Ma. The other dates are interpreted away, but this is unreasonable.

Reply: We sincerely thank the reviewer for this insightful comment. We agree entirely that the terms "date" and "age" must be distinguished with care. In the revised manuscript, we have

systematically used "date" to refer to individual isotopic results and "age" for the interpreted geological event, in line with standard geochronological practice.

Regarding the spread in dates from 4410 Ma to 4360 Ma, we acknowledge that this is a critical point of interpretation. The four analyzed zircon grains are texturally and compositionally uniform, supporting a co-genetic origin. In Apollo breccia samples, zircon grains within the same lithic clast are typically interpreted as originating from the same crystallization event (Quick et al., 1981). The spread of U-Pb dates is commonly attributed to partial Pb loss due to subsequent thermal or impact events, a well-documented phenomenon in lunar zircons (e.g., Zhang et al., 2012). This is consistent with the complex impact history of the sample.

Therefore, in accordance with established protocols for interpreting discordant datasets from thermally disturbed terrains, the oldest date within a coherent cluster is often considered the most reliable estimate of the primary crystallization age, as it is least affected by later disturbance. The alternative that the ~50 Ma spread represents a prolonged crystallization interval is considered less likely, given the textural evidence for a single recrystallization event.

We have clarified this reasoning in the revised text, emphasizing that the 4410 ± 8 Ma represents our interpretation of the most robust estimate for the timing of zircon crystallization.

Revision (**Lines 122-128**): Although the eleven analyses show a small range of dates (from 4,410 to 4,354 Ma) with a variation of about 1%, the ~50 Ma spread impossibly represents a prolonged recrystallisation interval due to the textural and compositional uniformity of the analysed zircons. These results likely reflect minor Pb loss during post-crystallisation impact or thermal events, a well-recognised feature observed in ancient lunar zircons[40,41]. Therefore, we interpret the oldest date of 4,410 ± 8 Ma (Fig. 3) as representing the zircon formation age.

- L144: The “recrystallization temperature” is based on rutile-saturation, which does not apply here. Also, the clast with the zircons has no ilmenite documented, hence a(Ti) is undetermined and this thermometer cannot be applied.

Note that this temperature is identical or superior to the crystallization temperature of anorthosites (see review of experimental constrains by Schmidt+Krattli), why would it be a recrystallization T?

Reply: Thanks for this critical comment regarding the application of the Ti-in-zircon thermometer. We fully agree that the calibration based on rutile saturation is inappropriate for our sample, as the absence of a co-existing Ti-buffering phase (rutile or ilmenite) means the activity of TiO_2 ($aTiO_2$) is unconstrained. Thus, we have removed this temperature calculation in the revised manuscript. Instead, we now rely on robust mineralogical evidence to constrain the thermal conditions. The presence of tridymite—a high-temperature silica polymorph stable above ~1000 °C—within the recrystallized domain provides a firm lower bound of temperature constraint. This rough temperature estimate would not affect the major conclusions of the study.

The reviewer rightly notes that this estimated temperature is comparable to, or even exceeds, the crystallization temperature of primary anorthosites. However, studies of Apollo granulitic fragments have documented that recrystallization temperatures can indeed exceed those of initial magmatic crystallization, particularly when driven by transient thermal events such as impacts (Çushing et al., 1999; Pernet-Fisher et al., 2022, 2023). This is consistent with the granoblastic textures and ~120° triple junctions observed in EBSD and CL images, which collectively support a high-temperature recrystallization origin rather than primary crystallization.

Revision (**Lines 149-152**): The presence of tridymite (Fig. 1c), a high-temperature silica polymorph commonly stable above 1,000 °C (ref. [46]), provides a lower bound of temperature attained during the recrystallisation event. This inferred temperature is consistent with the formation conditions of lunar granulites[47].

- L. 163: Why is KREEP post LMO? KREEP is the ultimate residual melt of the LMO, so it is definitely part of the LMO crystallization.

Reply: We appreciate this valuable clarification. We agree that KREEP represents the residual melt of the LMO and should be regarded as syn-LMO rather than post-LMO. Our original phrasing was imprecise. We intended to convey that the intrusion of the KREEP-

component bearing Mg-suite magmas into the primary anorthositic crust occurred after the main phase of LMO solidification. This is a distinctly post-LMO event, not that KREEP itself formed after the LMO. We recognize that the wording post-LMO KREEP was misleading and have revised in the manuscript to make this distinction clearer and to avoid any ambiguity.

Revision (**Lines 168-171**): These observations, coupled with the old recrystallisation age of 4.41 Ga, align with thermal[47,51] and chemical[52] inputs from KREEP-rich Mg-suite magmas—the earliest post-LMO intrusions permeating the primary crust[53].

- L. 166: There is confusion here. Now the argument is KREEP-component bearing high Mg-magmatism.

Reply: We thank the reviewer for highlighting this lack of clarity again in our manuscript. We have modified the manuscript to clearly separate the syn-LMO urKREEP and post-LMO Mg-suite, thereby avoiding conceptual ambiguity. The text now explicitly states that the Mg-suite magmas acquired their KREEP signature by assimilating syn-LMO urKREEP during their post-LMO intrusion.

Revision (**Line 172-174**): Thus, the zircon-bearing domain serves as a diagnostic marker of early crustal reworking through KREEP-component bearing Mg-suite magmatism.

- L. 169ff: These arguments are not acceptable and logically flawed. No matter what the details are, the petrological process of magma ocean crystallization will result in similar rock types formed upon final crystallization. The nature of crystallization process is that they converge and give the same rocks even if primary compositions and volumes might differ. This does not allow to conclude anything on the global scale tectonics, but only confirms petrological principles when approaching a eutectic. Yes, there was a magma ocean on the far-side, but how this magma ocean crystallized, how thick the resulting crust and which detailed composition cannot be extracted from a few millimetric clasts.

Reply: We thank the reviewer for this insightful comment, which helps us refine the scope and interpretation of our findings. We fully agree with the fundamental petrological principle

that the crystallization of a magma ocean will converge towards similar residual rock types, such as ferroan anorthosites, regardless of potential initial volume or compositional asymmetries. The mineralogical similarities we observe are consistent with this predictable late-stage LMO process.

However, while the final *rock types* may be similar, the detailed *chemical composition* of these rocks (e.g., Mg# of mafic minerals, trace element patterns) is highly sensitive to the specific crystallization path of the LMO, which can be influenced by factors such as cooling rate and mantle overturn (Wasson & Warren, 1980; Loper & Werner, 2002; Sio et al., 2020). The key point from our study is that the farside anorthosites are not only mineralogically similar to their nearside counterparts but also exhibit closely comparable major and trace element compositions. Our intention was not to reconstruct the precise details of the farside LMO crystallization (e.g., crustal thickness, minor compositional heterogeneities) from a limited number of clasts, which would indeed be unjustified. Rather, the key implication of our data is that some primary *building blocks* of the crust on both hemispheres are petrologically similar. This finding suggests that the pronounced geochemical and structural dichotomy observed remotely is unlikely to be a direct consequence of a fundamentally different LMO crystallization path.

Therefore, while our sample set is indeed limited for reconstructing global-scale tectonic scenarios, the chemical consistency between hemisphere anorthosites allows us to propose that the observed remote-sensing dichotomy is more likely a product of post-LMO modification than a reflection of a primordially asymmetric LMO. We have significantly revised the manuscript to tone down any overreaching tectonic interpretations and to generate and to frame our conclusions more cautiously, focusing on the petrological significance of the sample similarities within the established framework of LMO crystallization.

Revision (**Lines 188-198**): The compositional and petrological similarity of CE-6 farside anorthosites with Apollo nearside FANs indicates the widespread distribution of this lithology across the Moon, challenging models of primordial hemispheric asymmetry arising from LMO differentiation. The near-identical plagioclase anorthite contents ($An_{95–98}$) and REE concentrations and mafic mineral Mg# values (33–70) across both lunar hemispheres (Fig. 2,

Extended Data Figs. 2–4) suggest that at least some primary building blocks of the global lunar crust are compositionally comparable. As the mineral chemical compositions in anorthosites are highly sensitive to the specific crystallisation path of the LMO[30,67], the observed mineralogical uniformity implies that both the nearside and farside experienced similar late-stage LMO crystallisation process and primary crust flotation. This finding suggests that the crustal geochemical dichotomy observed remotely[5–8] unlikely resulted from intrinsically different LMO crystallisation paths[9,10].

- L. 208: Why do you expect a precise age for the final crystallization of the lunar magma ocean? This might be well different in different places, models showing that the final crystallization is protracted over 200 Ma, meaning that anorthositic cumulates might be forming over 200 Ma. Pernet-Fisher et al. showed that anorthosites involve a lot of reworking, consistent with a crust on a giant magma ocean that forms over >100 Ma, this is completely ignored.

Reply: We sincerely thank the reviewer for raising this crucial point regarding the timescale of LMO crystallization and the interpretation of anorthosite ages. We fully agree that the late-stage crystallization of a global magma ocean may have been a prolonged process, as evidenced by the wide range of anorthosite ages and their complex reworking history highlighted in studies such as Pernet-Fisher et al. (2016, 2019).

Our intention is not to propose a single, precise age for the *global* termination of the LMO. Instead, we argue that the formation of urKREEP marks a specific endpoint of the crystallization sequence. The ~4.41 Ga zircon age obtained from our farside sample does not represent a crystallization age of the anorthosite but dates a thermal, assimilation event involving KREEP-component bearing melts. Since these melts could only exist after the extraction of urKREEP from the crystallizing LMO, this age provides a robust *local lower limit* for the cessation of LMO differentiation in the farside crustal source region. It does not preclude a prolonged (>100 Ma) global solidification process but establishes a key chronological benchmark for the endpoint of LMO solidification on one hemisphere.

We have added the above discussion and revised the manuscript to clarify that this age signifies the timing of post-LMO crustal reworking and provides a constraint on the *minimum* age for local LMO solidification in the farside.

Revision **Lines 217-221**: Models show that the late-stage LMO crystallisation may have been a prolonged process [74,75], as also evidenced by the wide range of anorthosite ages[17–20,22,42] and its complex reworking history[38,76]. The formation of the urKREEP reservoir marks a specific endpoint of the LMO crystallisation sequence[68]; however, the timing of this terminal solidification has remained a long-standing controversy.

Lines 224-231: The Pb-Pb zircon date of 4,410 ± 8 Ma from CE-6 anorthosites now provides a critical chronological benchmark for the termination of farside LMO solidification. This date, representing the timing of crustal reworking by intrusions enriched in KREEP component, necessitates that both formation of farside FANs and the urKREEP reservoir occurred prior to 4.41 Ga (Fig. 4). This chronology establishes the first robust, local lower limit for the cessation of farside LMO solidification and suggests rapid crustal stabilisation within the first 150 million years after Solar System formation.

In summary, the conclusions are largely based on the choice of initial assumptions and translate into a completely static view of what happened during magma ocean crystallization. On principal, there can't be a precise age of anorthosite formation, and whatever the details are, the late crystallization products are governed by petrological principles and not by global dynamics or tectonics. The manuscript does hence not plausible constrain processes that led to the dichotomy in the lunar crust.

Reply: We sincerely thank the reviewer for this thoughtful summary, which helps us better to position the scope and implications of our study. We fully agree that the crystallization of a global magma ocean is fundamentally governed by petrological principles, and that late-stage products are expected to converge towards similar lithologies, irrespective of potential initial hemispheric asymmetries. We also concur that the formation of anorthosites is a protracted process, and that seeking a single, precise age for their formation is not geologically meaningful.

In light of these principles, we have reframed the conclusions of our manuscript to avoid any overinterpretation. Our primary goal is not to present a deterministic, static model of LMO evolution, nor to directly resolve the origin of the lunar crustal dichotomy. Instead, we aim to report the first petrological and geochemical evidence from farside anorthositic clasts. The key

observation is that these farside anorthosites are compositionally comparable to nearside FANs. This similarity suggests that the primary crust-building materials on both hemispheres shared a common petrogenetic origin, providing a critical, direct petrological constraint on the lunar crustal asymmetry debate. Furthermore, the ~4.41 Ga zircon date provides a crucial local lower limit for the solidification of the farside LMO source region. While not defining a global timeline, this datum places an important constraint on the early thermal evolution of the farside. We have carefully revised the manuscript to ensure our interpretations remain within the bounds supported by the data, focusing on how these new findings provide tangible constraints for future models of lunar crustal formation and asymmetry (**Lines 251-258**).

We believe that the revisions made in response to your comments have significantly strengthened the manuscript, and we thank you again for your rigorous and constructive review.

REFERENCES in REPLY:

Buddington A F. Adirondack igneous rocks and their metamorphism[J]. (1939).

Çushing, J. A., Taylor, G. J., Norman, M. D. & Keil, K. The granulitic impactite suite: Impact melts and metamorphic breccias of the early lunar crust. *Meteorit. Planet. Sci.* 34, 185–195 (1999).

Grange, M. L., Nemchin, A. A., Timms, N., Pidgeon, R. T., & Meyer, C. Complex magmatic and impact history prior to 4.1 Ga recorded in zircon from Apollo 17 South Massif aphanitic breccia 73235. Geochim. Cosmochim. Acta 75(8), 2213-2232 (2011).

Loper, D. E. & Werner, C. L. On lunar asymmetries 1. Tilted convection and crustal asymmetry. *J. Geophys. Res. Planets* 107, (2002).

Nemchin, A. A., Pidgeon, R. T., Whitehouse, M. J., Vaughan, J. P., & Meyer, C. SIMS U–Pb study of zircon from Apollo 14 and 17 breccias: implications for the evolution of lunar KREEP. *Geochim. Cosmochim. Acta* 72(2), 668-689 (2008).

Pernet-Fisher, J. F. & Joy, K. H. The lunar highlands: old crust, new ideas. *Astron. Geophys*. 57, 1.26-1.30 (2016).

Pernet-Fisher, J. F., Deloule, E. & Joy, K. H. Evidence of chemical heterogeneity within lunar anorthosite parental magmas. *Geochim. Cosmochim. Acta* 266, 109–130 (2019).

Pernet-Fisher, J. F. & Joy, K. H. Thermal metamorphism on the Moon as recorded by the granulite suite. *J. Geol. Soc.* 179, jgs2021-044 (2022).

Pernet-Fisher, J. F., Joy, K. H. & Hartley, M. E. Using lunar granulites to constrain re-equilibration timescales of contact thermal metamorphism on the Moon. J. *Geophys. Res. Planets* 128, e2022JE007570 (2023).

Quick, J. E., James, O. B., & Albee, A. L. Petrology and petrogenesis of lunar breccia 12013. *Lunar Planet. Sci. Conf. Proc.* (1982).

Sio, C. K., Borg, L. E. & Cassata, W. S. The timing of lunar solidification and mantle overturn recorded in ferroan anorthosite 62237. *Earth Planet. Sci. Lett.* 538, 116219 (2020).

Streckeisen, A. To each plutonic rock its proper name. *Earth-Sci. Rev.* 12, 1–33 (1976).

Wasson, J. T. & Warren, P. H. Contribution of the mantle to the lunar asymmetry. *Icarus* 44, 752–771 (1980).

Zhang, B., Lin, Y., Moser, D. E., Warren, P. H., Hao, J., Barker, I. R., ... & Bouvier, A. Timing of lunar Mg-suite magmatism constrained by SIMS U-Pb dating of Apollo norite 78238. *Earth Planet. Sci. Lett.* 569, 117046 (2021).

REVIEWER COMMENTS

Reviewer #1 (Remarks to the Author):

Dear Prof Chen and Co-Authors,

Thank you for your revision and consideration to my comments.

An extra comment, overall, we will ever know where the small lunar regolith samples come from and their tumbling around history on the lunar surface since 4.4+ Ga. Additionally, of course the Orientale Basin forming event could have targeted material as old as the zircon dated in the manuscript, since it is younger than the rock the zircon is within. Additionally, the target area of the event that formed Orientale was already covered by older ejecta from the many basin forming events prior to this last basin. These events excavated many old rocks. The stack of ejecta (i.e., the many ejecta stratigraphy) that would be also ejected upon the Orientale Basin event could have all sort of ages prior to the ~3.9 Ga event that formed Orientale Basin. The shock pressure is not all the same across the impacted area, or its effect on the targetted materials, and there is post-shock temperature increase gradient across the newly formed basin.

This is a deep set of caveats that shouldn't be ignored or brushed over, and therefore further consideration taken in the manuscript.

I hope there will be the day astronauts can do field work (ONLY) on the Moon, and really find the proper connection of rocks and their sources.

Kind Regards,
Vera Assis Fernandes

Reply: We thank the reviewer for this thoughtful and nuanced consideration regarding the complex provenance history of lunar regolith samples. We fully agree that the long-term surface reworking and the potential contribution of distant impact basins, such as Orientale, constitute fundamental caveats that must be thoroughly addressed in any study of returned lunar soils. The CE-6 landing site regolith is a mixture of materials derived from multiple sources, including both local and potentially distant impacts.

Given the extensive gardening and mixing processes on the lunar surface over billions of years, it is inherently difficult to trace the exact origin of individual regolith grains, especially for anorthosites widely distributed in the lunar highlands. While it is true that the ~3.9 Ga Orientale impact could have excavated and redistributed ancient crustal materials (including rocks as old as our zircon), the following independent lines of evidence point to a more likely farside source for our samples.

First, although the shock pressure is not uniform across the impacted area, long-distance ejecta is unlikely to avoid the shock effect completely. The zircon-bearing recrystallized domain holding low-strain, static recrystallization microtexture is inconsistent with shock

deformation.

Second, the ejecta from Orientale impact in the CE-6 landing site was estimated to constitute Imbrian plains (Jia et al., 2024; Qian et al., 2024; Gao et al., 2025) and covered by mare basalt (Meyer et al., 2020; Jia et al., 2025). The probability of being derived from a series of small impacts is also exceedingly low. Geological surveys suggested that the Vavilov basin within the farside highlands (Gou et al., 2024) and anorthositic regions within the Apollo basin (Guo et al., 2024, 2025) were the major possible sources of the CE-6 anorthosite material.

Third, our study does not attempt to establish a unique or deterministic provenance for each anorthositic clast. Instead, our objective is to assess whether the petrological, geochemical, and chronological characteristics preserved in the CE-6 anorthosites are consistent with those of the nearside counterpart, and whether these characteristics place meaningful constraints on the timing and nature of crust formation and reworking. While direct provenance for every analyzed clast cannot be established, the location and geological context of the Chang'e-6 landing site, combined with the microstructural characteristics of the anorthosite clasts and their coherent petrological and geochemical signatures, suggest that the majority—if not all—of the anorthositic clasts are derived from the farside highlands. Even though a nearside origin cannot be entirely excluded for all studied anorthosites, we can also conclude that the two hemispheres share consistent petrological and geochemical characteristics, and that the LMO evolved consistently.

The possibility of ancient material excavated and redistributed by Orientale or later impacts is additionally acknowledged within the revised manuscript. We fully agree with the reviewer's anticipation that future in situ geological field investigations on the Moon will ultimately be required to resolve the detailed context for sample sources (especially for anorthosites). Considering the current situation, the present study is a necessary step toward building the framework by using the first farside returned samples.

Revision: **Lines 176-183**: The provenance of individual regolith clasts in the severely gardened lunar surface is inherently complex, especially for anorthosites widely distributed across the lunar highlands. The CE-6 anorthosites could represent materials excavated and redistributed by a wide range of craters, even including nearside large impact basins such as Orientale, which was capable of distributing ancient material globally. However, several lines of evidence favour a predominantly local, farside origin for the CE-6 anorthositic clasts. Nearside large impacts would be expected to induce shock and plastic deformation of ballistic ejecta and/or reset their crystallisation age

Lines 189-192: The location and geological context of the CE-6 landing site, low-strain microtexture, and coherent petrological and geochemical signatures of the clasts consistently suggest a predominantly farside highland origin.

REFERENCES in REPLY:

Gao, A. *et al.* Geological Background of the Chang'e 6 Landing Site and the Provenance of Returned Samples. *J. Geophys. Res. Planets* **130**, e2024JE008658 (2025).

Gou, S. *et al.* Complex basalt evolution in the Chang'e-6 landing area. *Earth Planet. Sci. Lett.* **648**, 119091 (2024).

Guo, D. *et al.* Geological investigation of the lunar Apollo basin: From surface composition to interior structure. *Earth Planet. Sci. Lett.* **646**, 118986 (2024).

Guo, D. *et al.* Crystalline Ferroan Anorthosite Identified in the Lunar Apollo Basin. *J. Geophys. Res. Planets* **130**, (2025).

Jia, Z. *et al.* Geologic context of Chang'e-6 candidate landing regions and potential non-mare materials in the returned samples. *Icarus* **416**, 116107 (2024).

Jia, B., Fa, W. & Zhang, M. The Chang'E-6 Landing Region: Geologic Context, Mare Basalt, and Regolith Properties. *J. Geophys. Res. Planets* **130**, e2025JE009018 (2025).

Meyer, H. M., Denevi, B. W., Robinson, M. S. & Boyd, A. K. The Global Distribution of Lunar Light Plains From the Lunar Reconnaissance Orbiter Camera. *J. Geophys. Res. Planets* **125**, e2019JE006073 (2020).

Qian, Y. *et al.* Extensive Intrusive Magmatism in the Lunar Farside Apollo and South Pole–Aitken Basins, Chang'e-6 Landing Site. *Astrophys. J. Lett.* **971**, L39 (2024).

Reviewer #2 (Remarks to the Author):

Overall, the authors have done a great job to improve the manuscript and to much clearer distinguish between observations and interpretations. Some over-interpretations have been removed, but not all.

Reply: We thank the reviewer for acknowledging the improvements made in our previous revision and for the rigorous, constructive feedback, which is invaluable for further refining the precision and scope of our interpretations. We have addressed each point below and carefully re-examined the manuscript to identify and remove any remaining statements that could be perceived as over-interpretation. We hope that this revision, guided by the reviewers' comments, will further enhance the precision and comprehensiveness of the discussion in our manuscript.

The ages of the anorthositic nearside crust are widespread. You have one sample, please do not conclude that the entire farside anorthositic crust has been solidified based on one sample, solidification begins with more or less crystallized rafts in a magma ocean and continues with occasional reworking (e.g. Pernet-Fisher). Your data can only show that partial solidification

has occurred by 4.41 Ga, but not that the anorthositic crust was completely formed at 4.41 Ga; see Fig. 3). Physically, the end of crystallization will be very protracted as the final solidification within a crystal mush is extremely protracted and will be locally quite different.

Reply: We thank the reviewer for this important clarification regarding the temporal interpretation of our Pb-Pb date, and entirely agree that our single zircon date cannot conclude complete solidification of the farside anorthositic crust by 4.41 Ga. The protracted nature of final-stage LMO evolution is well illustrated by the crystal mush model and is consistent with the broad age distribution recorded by FANs and KREEP. In light of this, we have refined our interpretation. The 4.41 Ga date marks a local thermal reworking event, providing a robust lower bound on the timing of partial solidification and crustal stabilization in the specific region, rather than the final cessation of the global anorthositic crust. Accordingly, the manuscript now emphasizes the local, minimum-age nature of this constraint. Correspondingly, Fig. 3 has been updated to illustrate a local age constraint inferred from our data, rather than the completion of crustal formation. These adjustments ensure that our conclusions are precisely aligned with the petrological framework highlighted by the reviewer. Importantly, even within the more constrained interpretation, our sample provides the first local snapshot for the farside LMO solidification and a meaningful comparison with the pace and timescale of LMO evolution inferred from nearside samples.

Revision: **Lines 28-30**: High-precision lead-lead dating of zircon constrains this reworking event to 4,410 ± 8 Ma, establishing the first local lower bound for farside LMO solidification.

Line 128: Fig. 3, with an addition of the word 'local'.

Line 216: Models of local farside LMO solidification and crustal reworking.

Lines 229-236: The Pb-Pb zircon date of 4,410 ± 8 Ma from CE-6 anorthosites now provides a critical local lower bound for farside LMO solidification. This date, representing the timing of crustal reworking by intrusions enriched in KREEP component, necessitates that both the formation of local farside FANs and the urKREEP reservoir in their source region occurred prior to 4.41 Ga (Fig. 4). Although the LMO solidification may be protracted spatially, this chronology establishes the first robust, local snapshot for the cessation of farside LMO solidification and suggests rapid stabilisation of a crustal portion within the first 150 million years after Solar System formation.

Lines 249-250: The local lower bound for farside LMO solidification constrained here is broadly consistent with the younger limits for nearside terminal LMO crystallization

Lines 263-264: The recrystallisation age of 4.41 Ga serves as a pivotal snapshot of the termination of farside LMO solidification, anchoring the timeline of early lunar evolution.

Regarding the lunar asymmetry, I realize that reviewer 1 and me have a somewhat disparate opinion here and that the authors may naturally struggle a bit to satisfy both.

Reply: We appreciate the reviewer's understanding. Both reviewers' insights have significantly strengthened the clarity and robustness of our manuscript. Despite the

complexities involved in sample provenance and thermal history, the data point to a uniform conclusion: they confirm the consistent petrological and geochemical signatures between the anorthosites on both lunar hemispheres and provide a direct, local, early, lower time-bound for the farside LMO evolution.

Nevertheless, the asymmetry cannot form during a magma ocean stage with a true liquid layer (i.e. not as long as there is a magma layer that has crystals suspended). This is nicely pointed out by Nimmo et al. 2024, Nature (cited as reference #84), who clearly state that the freezing-in of the Moon's bulge requires a rigid elastic layer. The main topic of this paper is that there should be a tidally driven re-melting event at about 4.35 Ga due to the Moon's transition of the Laplace plane (at which point the Moon is not yet tidally locked), driving e.g. the formation of the (KREEP bearing) high-Mg suite. The Nimmo paper remains unclear whether this tidal remelting also effects the anorthositic crust (the carton of their figure 1 would indicate yes), yet points out the resetting of the zircon ages corresponding to this tidal remelting.

The implications I draw from the Nimmo paper (and references contained therein) for this manuscript are: the asymmetry is not directly related to the magma ocean (great support for the hypothesis of the authors), but at the same time, the asymmetry is said to have been there at 4.35 Ga. This is not a contradiction to a 150-200 Ma span for LMO solidification (Maurice et al.), as the waning stage is a (possibly not anymore global) crystal mush that may be quite rigid. Further, the zircon resetting may not need a heat source external to the observed rock fragment (i.e. the Mg-suite), if tidal heating alone can do it. This can't be resolved at this point, but needs to be mentioned/discussed.

In this sense, I do not see the pertinence of the cartoon of Fig.4, in any case, I have serious qualms with this "contact metamorphism" through high-Mg suite magmas. This contact metamorphism will affect an infinitesimal small volume of the anorthositic crust and the likelihood that your sample comes exactly from such a contact aureole ought to be similarly small. A rather widespread tidal remelting seems to be much more plausible.

Reply: We thank the reviewer for bringing the important work of Nimmo et al. (2024) to our attention and for the constructive suggestion to consider tidal heating as a potential alternative or complementary heat source for the observed recrystallization and zircon resetting. We fully agree that the lunar crustal asymmetry cannot originate from a true liquid magma ocean stage, as pointed out by Nimmo et al., and that the freezing-in of a rigid elastic layer is required. Their proposal of a tidally driven remelting event at ca. 4.35 Ga, associated with the Moon's transition of the Laplace plane, provides an elegant explanation for the widespread ca. 4.35 Ga ages among lunar samples, including the formation of Mg-suite rocks enriched KREEP components. Our 4.41 Ga local farside age provides direct evidence that such rigidity existed prior to 4.35 Ga, strongly supporting the proposed tidal remelting event at this time.

We also agree that tidal remelting represents one of the most plausible and energetically

favorable explanations for the large-scale thermal event at ~4.35 Ga. We consider the contribution of KREEP-rich Mg-suite magma because the zircon-bearing anorthosite clast records not only *high-temperature metamorphism* but also *the addition of KREEP components* (Fig. 2b). Importantly, this contact metasomatism by Mg-suite magma is not mutually exclusive with the tidal heating model; rather, the two processes may be causally linked. Before this major thermal episode at ~4.35 Ga, earlier tidal heating could have promoted local Mg-suite magmatism or KREEP-rich magmatism at depth. This magma, underplating the crust, would have induced contact metamorphism, resulting in the recrystallized domain observed in our sample. Thus, tidal remelting and Mg-suite underplating are not competing but complementary mechanisms, with tidal heating as the potential driving force.

Although our zircon age of 4.41 Ga predates the peak of this proposed tidal event, it may record an early phase of thermal disturbance that ultimately led to the ca. 4.35 Ga resetting peak. The generation of KREEP-bearing magma and the local thermal-chemical reworking recorded in our sample can thus be interpreted as likely outcomes of such tidal heating event. Accordingly, we have expanded the discussion to acknowledge that while KREEP-rich magmatism offers a direct, self-consistent explanation for both the thermal and geochemical signatures, tidal remelting may represent the underlying driving mechanism. Figure 4 has also been revised to reflect the potential contribution of tidal heating. The model highlighted by the reviewer substantially strengthens our discussion and aligns our interpretation with a broader, current mechanism consistent with recent models.

Revision: **Lines 168-171**: These observations, coupled with the old recrystallisation age of 4.41 Ga, align with either thermal and chemical inputs from KREEP-rich Mg-suite magma or with tidal heating that mobilised KREEP components at depth.

Lines 173-174: Thus, the zircon-bearing domain serves as a diagnostic marker of early crustal reworking through KREEP-rich magmatism and metasomatism.

Lines 217-219: crystallisation of FANs forms the primary farside crust, while residual urKREEP accumulates beneath. b, Post-LMO stage (ca. 4.41 Ga): regional tidal heating and/or KREEP-rich magma thermally rework the primary crust, triggering granulite-facies metamorphism and metasomatic enrichment.

Lines 254-257: In addition, the metasomatic assimilation of KREEP-rich magma at 4.41 Ga demonstrates that the early farside mantle retained sufficient thermal energy—potentially supplemented or driven by tidal heating—to sustain post-LMO magmatism, mirroring nearside processes.

Lines 260-261: but instead resulted from post-LMO crustal reworking processes, such as tidal heating or large impacts.

Regarding the discussion of zircon morphology: Ext.Data Figures 5 and 6 fail to show any subhedral zircon, its simply not there. Yet, I do not understand why this is such an issue, i.e. why insist on eu- or subhedral shapes. If there was a remelting/recrystallization event that went beyond zircon saturation, the zircons could have any shape.

Reply: We thank the reviewer for this clarification. We agree that if a remelting or recrystallization event exceeded zircon saturation, the resulting zircons could exhibit a range of morphologies, and insisting on euhedral or subhedral shapes is unnecessary. In the revised manuscript, we have therefore de-emphasized the description of zircon morphology and instead focus on the key observation that the zircons are texturally intergrown with recrystallized plagioclase and record a recrystallization date of 4,410 ± 8 Ma, which we interpret as the timing of high-temperature static recrystallization. The morphological description has been simplified to avoid any overstatement, and the discussion now centers on the chronological significance of the zircon age rather than its crystal shape. We believe this approach addresses the reviewer's concern while maintaining the scientific integrity of our interpretation.

Revision: **Lines 144-146**: The zircons are in textural equilibrium with the fine grained plagioclase and exhibit homogeneous CL structures (Extended Data Fig. 5) and uniform compositions (Supplementary Table 5), supporting a co-genetic origin.

As I checked some references in the manuscript, I have seen that there are 87 of them, while standard Nature policy is ~50. Although this is an editorial concern, I wonder how the main text arguments will look like once 1/3 of the references are cut.

Reply: We thank the reviewer for noting this issue. We recognize that the current reference count in the previous version exceeds the standard guidelines for *Nature Communications* (no more than 70). We have carefully reviewed and consolidated the reference list, removing redundant or non-essential citations while preserving those critical to the main arguments. The current manuscript contains 70 references.

Finally, the last (added statement) of the bold paragraph is only very indirectly true. The arguments of this manuscript place the reasons for the lunar dichotomy elsewhere, how does this "help to better understand lunar dichotomy"?

Reply: We thank the reviewer for this constructive suggestion to clarify the contribution of our study to understanding the lunar crustal dichotomy. To address this point, we have revised the final sentence of the abstract to articulate the implications of our findings clearly.

Revision (**Lines 30-32**): These findings establish a critical chronological benchmark for farside LMO solidification and offer direct evidence of a comparable primary crust across the Moon, constraining the origin of the crustal dichotomy to post-LMO reworking.

REFERENCES in REPLY:

Nimmo, F., Kleine, T. & Morbidelli, A. Tidally driven remelting around 4.35 billion years ago indicates the Moon is old. *Nature* **636**, 598–602 (2024).

REVIEWER COMMENTS

Reviewer #2 (Remarks to the Author):

There is still some uneasiness from both reviewers about considering adequate (yet inherent) uncertainties. On the Moon, any interpretation need to be put into context, i.e. other possibilities in genesis need to be acknowledged. This is my third reading of the manuscript and I believe that we arrived more or less where one can arrive. I would ask the authors, to make another effort to claim a little bit less certainty, where certainty does not exist (the arguments have all been made in the previous reviews), as there is really nothing to be gained but much credibility to be lost by affirming too much. There are large uncertainties in any of the lunar geology/selenology and I believe the text could still be improved with respect to these, i.e. some wording is still too affirmative.

I have only two minor comments: (1) The revisions now state that "The recrystallisation age of 4.41 Ga serves as a pivotal snapshot of the termination of farside LMO solidification, anchoring the timeline of early lunar evolution.". This is simply incorrect, the 4.41 dates the latest beginning or in other words that some of lunar farside anorthosite has formed, but certainly not all of the lunar farside. You simply don't know when termination/full crystallization of the lunar magma ocean occurred.

Reply: We thank the reviewer once again for the careful reading and constructive feedback. We fully recognise the importance of acknowledging uncertainties in lunar geological interpretations. Accordingly, we have further revised the manuscript to temper overly affirmative statements. We agree that the 4.41 Ga age documents a local thermal reworking event and provides a lower bound on the timing of farside crustal regional stabilization, but it does not constrain the global completion of LMO solidification. The statement in question has been revised to remove any implication of global LMO completion, and similar instances of overinterpretation have been carefully re-evaluated throughout the manuscript.

Revision: **Lines 1-2 (title):** Chang'e-6 farside anorthosites indicate hemispherically comparable magma ocean solidification

Lines 25-26: These clasts exhibit both mineralogical and compositional similarity with nearside Apollo FANs, supporting a comparable LMO-derived primary crust on both hemispheres.

Line 242: Our data offer new constraints on the potential synchronicity of late-stage LMO solidification between the two hemispheres.

Lines 268-270: The recrystallisation age of 4.41 Ga thus provides a lower bound for the formation of a farside crustal portion, anchoring a key point in the timeline of early lunar evolution.

(2) The zircons are xenomorph and their grain shape cannot be taken as indicating any equilibrium or paragenetic relation, from their shape, they could be e.g. be impact detrital. Unless the authors provide other BSE images, this has be taken into account.

Reply: We thank the reviewer for this important clarification. We fully acknowledge that the xenomorphic shape of the zircon grains alone does not demonstrate equilibrium or paragenetic

relationships, and that an impact-detrital origin is a valid alternative interpretation. Accordingly, we have revised the manuscript to explicitly consider this possibility.

Specifically, we now state that the zircons "either formed during recrystallisation or represent impact-derived detrital zircon." The low-strain microstructure (<3° misorientation) and ~120° triple-junction grain boundaries documented in plagioclase (Extended Data Fig. 6b) is consistent with static recrystallization and minimal post-thermal impact-deformation, as impact processes typically produce high dislocation densities and plastic deformation. In this regard, if the zircons have an impact-detrital origin, the impact most likely happened prior to the static, high-temperature thermal recrystallisation of the anorthosite. Therefore, although xenomorphic zircon grains may have an impact-derived origin before recrystallisation, high-temperature metamorphism would be expected to reset their Pb-Pb systematics, consistent with homogeneous CL structures and uniform compositions in the zircons. As a result, the zircons would record the timing of the thermal event rather than their original formation. The impact-detrital possibility does not alter our conclusion that the 4.41 Ga age dates the high-temperature recrystallization event, but it adds an alternative explanation for the presence of zircon in the anorthosite. The manuscript has been revised accordingly to clarify this case and to avoid any implication that zircon morphology alone supports the interpretation.

We believe that this revision addresses the reviewer's concern by making our logic transparent and by avoiding any ambiguous description that might have been misinterpreted as depending on zircon morphology. We are grateful to the reviewer for helping us improve the clarity of our presentation.

Revision: **Lines 86-88:** Four zircon grains within this domain occur as xenomorphic, interstitial crystals in contact with plagioclase (Figs. 1c, d, Extended Data Figs. 5, 6), indicating that they either formed during recrystallisation or represent impact-derived detrital zircon.

Lines 116-118: The zircons exhibit homogeneous CL structures (Extended Data Fig. 5) and uniform compositions (Supplementary Table 5), indicating that they formed or were reworked during a recrystallisation event.

Lines 147-150: The weak cumulative misorientation angles (<3°, Extended Data Fig. 6b) and characteristic Raman peaks of the fine plagioclase grains (Supplementary Fig. 4) further indicate that these grains preserve low-strain crystallographic fabrics, consistent with static recrystallisation and minimal post-thermal impact deformation[30].

Lines 153-156: Although xenomorphic zircon grains may have an impact-derived origin before recrystallisation, high-temperature metamorphism would be expected to reset their Pb-Pb systematics, causing them to record the timing of the thermal event rather than their original formation age.
